# Association of ambulance and helicopter response times with patient survival: A systematic literature review and meta-analysis

**DOI:** 10.1371/journal.pone.0335665

**Published:** 2025-11-17

**Authors:** Peter Martin Hansen, Martine Siw Nielsen, Marius Rehn, Annmarie Lassen, Anders Perner, Søren Mikkelsen, Anne Craveiro Brøchner

**Affiliations:** 1 Prehospital Research Unit, Region of Southern Denmark, Odense, Denmark; 2 Department of Anesthesiology and Intensive Care Medicine, Odense University Hospital Svendborg, Svendborg, Denmark; 3 Department of Regional Health Research, University of Southern Denmark, Odense, Denmark; 4 Department of Anesthesiology and Intensive Care Medicine Kolding, Sygehus Lillebælt, Kolding, Denmark; 5 Air Ambulance Department, Division of Prehospital Services, Oslo University Hospital, Oslo, Norway; 6 Department of Research and Development, Norwegian Air Ambulance Foundation, Oslo, Norway; 7 Institute of Clinical Medicine, University of Oslo, Oslo, Norway; 8 Department of Emergency Medicine, Odense University Hospital, Odense, Denmark; 9 Department of Clinical Research, University of Southern Denmark, Odense, Denmark; 10 Department of Anesthesiology and Intensive Care Medicine, Rigshospitalet, Copenhagen, Denmark; 11 Department of Anesthesiology and Intensive Care Medicine, Odense University Hospital Odense, Odense, Denmark; Jazan University College of Applied Medical Science, SAUDI ARABIA

## Abstract

**Background:**

Only sparse scientific evidence supports the notion that the shortest possible response time relates to improved patient outcomes in acute conditions, other than out-of-hospital cardiac arrest and trauma. Confounders such as bidirectional causality and confounding by indication may influence patient-centered outcomes, which may prevent actionable conclusions from literature reviews. The purpose of the systematic literature review was to assess current evidence on association, if any, between ambulance and helicopter response times and survival in all patient categories treated by ambulance or helicopter services.

**Methods:**

The systematic search was conducted in MEDLINE, Cochrane Library, EMBASE, CINAHL, Scopus, and Clinical Trial Registries. All study designs and settings identified as relevant to the topic were eligible. The investigators retrieved data from a predefined template and extracted data from a predefined template. Two reviewers worked independently, and conflicts were resolved by a third reviewer. The researchers used PRISMA guidelines for abstracting data and GRADE methodology for assessing data quality and validity. As per study protocol, the primary study outcome was patient survival, and the main measurement was response time for emergency medical services vehicles.

**Results:**

The investigators included 115 studies that in total included 691,056 patients, comprising patients with out-of-hospital cardiac arrest, trauma, drownings, and including both adults and children in various settings. The overall median survival rate was 11.5% (IQR 5.2; 25.8). Response time varied between 1.10 and 48.62 minutes. The predefined domains and items of interest were accounted for in 46.7% of the included literature. In a meta-analysis of sub-groups, there was a positive correlation in selected populations. Certainty of evidence was very low as per GRADE methodology.

**Conclusions:**

This systematic review and meta-analysis found lack of evidence to infer an association between the EMS response time and patient survival, with very low certainty of evidence. The investigators found substantive research and knowledge gaps. Therefore, no actionable conclusions can be made from this systematic review.

## Introduction

### Background

In emergency medical services (EMS) context, response time is defined [1,2] as the time measured from the time a call is received by the dispatch centre to when the response vehicle arrives on scene. Based on tradition and best practice, response time for units such as ambulances, rapid response cars and helicopters is a key performance indicator and a controversial topic in debate on EMS coverage and performance. For critical conditions such as major trauma, short response time has shown to be pivotal [3]. Previous studies have, however, been ambiguous regarding the overall association of response time with patient outcome [4,5] and a recent statement paper [6] suggests the use of a set of clinical, safety, experiential and financial measures for evaluation of EMS effectiveness aside from response time.

### Importance

Limited scientific evidence supports the notion that the shortest possible response time relates to improved patient outcomes in acute conditions [7] other than out-of-hospital cardiac arrest and trauma [1,3]. Several confounders may influence patient-centered outcomes, including confounding by indication [8], which represent major limitations to the currently available, predominantly observational studies. Similarly, EMS-centered measures other than response time, such as prolonged on-scene times as the result of time-consuming complex medical procedures, and external factors including traffic congestion and bad weather, may affect the time to treatment in the hospital.

### Goals of the study

The aim of the systematic literature review was to provide an overview of the current body of evidence regarding the association between response times and survival in patients treated by ambulance and/or helicopter services. Thereby the investigators aimed to determine the association between overall emergency medical services unit response times and patient survival to enhance the knowledge in the field of dispatch acuity in EMS.

## Materials and methods

### Study design

This systematic literature review was conducted in accordance with the Cochrane Handbook [9] and The Joanna Briggs Institute Manual for Systematic Reviews [10]. The search strategy was developed in collaboration with a research librarian. The systematic review was registered in the International Prospective Register of Systematic Reviews, PROSPERO [11] on 10 September 2023, registration #CRD42023462339 [12] to comply with Preferred Reporting Items for Systematic Reviews and Meta-analyses (PRISMA) [13] guidelines for reporting systematic reviews. The protocol article for this review was published on 23 October 2023 [14].

### Data sources, search strategy and study selection

Two reviewers (PMH and MSN) independently screened all the titles and abstracts of the included studies for eligibility. A third reviewer (ACB) resolved conflicts. The researchers used Covidence® (Veritas Health Innovation, Melbourne, Australia) [15] and EndNote20® (Alfasoft AB, Gothenburg, Sweden) [16] software. The systematic search was conducted in MEDLINE (Ovid), Cochrane Library (Wiley), EMBASE (Ovid), CINAHL (EBSCO), Scopus (Elsevier), and Clinical Trial Registries. No time limits were imposed to avoid missing relevant literature. Furthermore, the investigators conducted forward and backward citation searches and the investigators report the search in accordance with the PRISMA extension for searching [17]. To avoid missing relevant literature, reference lists of the included literature were scanned for additional publications relevant to the review. The investigators searched databases 5–16 October 2023 and repeated the search on 28 September 2024.

The investigators also included non-indexed/grey literature and searched Web of Science, Google Scholar, www.ndltd.org, www.dart-europe.org, www.opengrey.eu, and www.oatd.org. The investigators performed those searches on 11–14 November 2023 and repeated the search on 24–25 September 2024.

Authors of all the included conference abstracts and thirteen of the published papers were contacted for clarification and provided additional data (S1 File). Two amendments were added and timely registered in the protocol [12] (S1 File).

Additional information such as meta-data for literature search, data extraction details and author contacts is available in an open-access repository (https://zenodo.org/records/15076410).

### Eligibility criteria

All study designs were eligible, apart from reviews and meta-analyses, including qualitative, quantitative, and mixed methods designs. The investigators included all articles in English, Scandinavian, Icelandic, German, French, and Portuguese in this systematic review. For potentially eligible articles in other languages with an English abstract, the investigators used Google Translate.

The investigators formulated the review questions using a Population, Exposure, and Outcome – *PEO* – framework [18].

#### Population.

Studies examining ambulance and helicopter response times in EMS were included. Studies describing all patient categories with the need for ambulance or helicopter delivered care in the prehospital environment were included.

#### Exposure.

Defined as treatment by ambulance and/or helicopter with recorded response time.

#### Outcomes.

***Primary outcomes*:** Patient survival defined as a composite outcome of 30-day survival and survival to discharge.

***Secondary outcomes:*** Impaired functional and neurological outcome as per validated measures.

### Data extraction

Data were collected from a pre-defined data extraction template by two reviewers (MSN and PMH), working independently. A third reviewer (ACB) resolved conflicts during that process. The data extraction template, validated from known references [19,20], comprised five domains: geo-political setting, EMS characteristics, EMS response characteristics, vehicle activation characteristics and response time characteristics (S1 File).

The patient-centered composite outcome comprising 30-day survival and survival to hospital discharge and secondary outcome measures such as cerebral performance category were extracted for each included study. Similarly, EMS-centered measures were recorded.

### Methodological quality assessment

To assess the risk of bias on the study level within the individual included studies, the researchers developed and applied a quality appraisal template, validated from known references [19–20]. Two reviewers (MSN and PMH) independently assessed the quality of the included literature. Conflicts were resolved by a third reviewer (ACB). The template domains were internal and external validity, displayed in S1 File.

For data synthesis, the biases in the quality assessment criteria were incorporated using the Grading of Recommendations Assessment, Development, and Evaluation (GRADE) [21] methodology. The investigators preferably used 30-day survival, and survival to hospital discharge when applicable, depending on what the study reported.

### Analysis

The primary composite outcome was patient survival as per 30-day survival/survival to hospital discharge. The investigators identified the patient-centered outcomes and EMS-centered measures in a tabular format, and used the tools provided by the GRADE [21] concept, including evaluation of risk of bias, inconsistency, indirectness, imprecision, and publication bias to assess the overall quality of evidence in a summary of evidence table and an overall quality of evidence (GRADE) [21] table. This review consistently reported the statistical significance as the strength of evidence against the model hypothesis, i.e., the individual studies’ null hypotheses. Therefore, the assessment is specific for sample size applied in the relevant study.

As per protocol, forest plots were produced to display effect estimates and confidence intervals for both individual studies and meta-analyses, using STATA 18.0 software (Stata Corp, College station, TX, USA). Each study is represented by a block at the point estimate of intervention effect with a horizontal line extending either side of the block. The size of the block represents the weight assigned to that study from inverse-variance method in the meta-analysis, whereas the horizontal line depicts the 95% confidence interval.

Accordingly, the confidence interval displays the range of intervention effects compatible with study results. The meta-analysis was conducted using a random effects model [22] to incorporate the assumption that the different studies are estimating different intervention effects that are related, as per the Der Simonian and Laird method [23]. To minimize the imprecision or uncertainty of the pooled effect estimate, the inverse-variance method was applied, allowing for larger studies, which have smaller standard errors, to be given more weight than smaller studies, which have larger standard errors. Subgroup meta-analyses for out-of-hospital cardiac arrest, traumatic out-of-hospital cardiac arrest, drowning, trauma, and miscellaneous were performed, respectively.

Forest plots for sub-group meta-analyses were produced to assess the individual study estimates of effect, using STATA 18.0 software (Stata Corp, College station, TX, USA). The meta-analyses were conducted to analyze adjusted odds ratios for the association between response time and patient survival.

## Results

### Identification of studies

**Fig 1:**
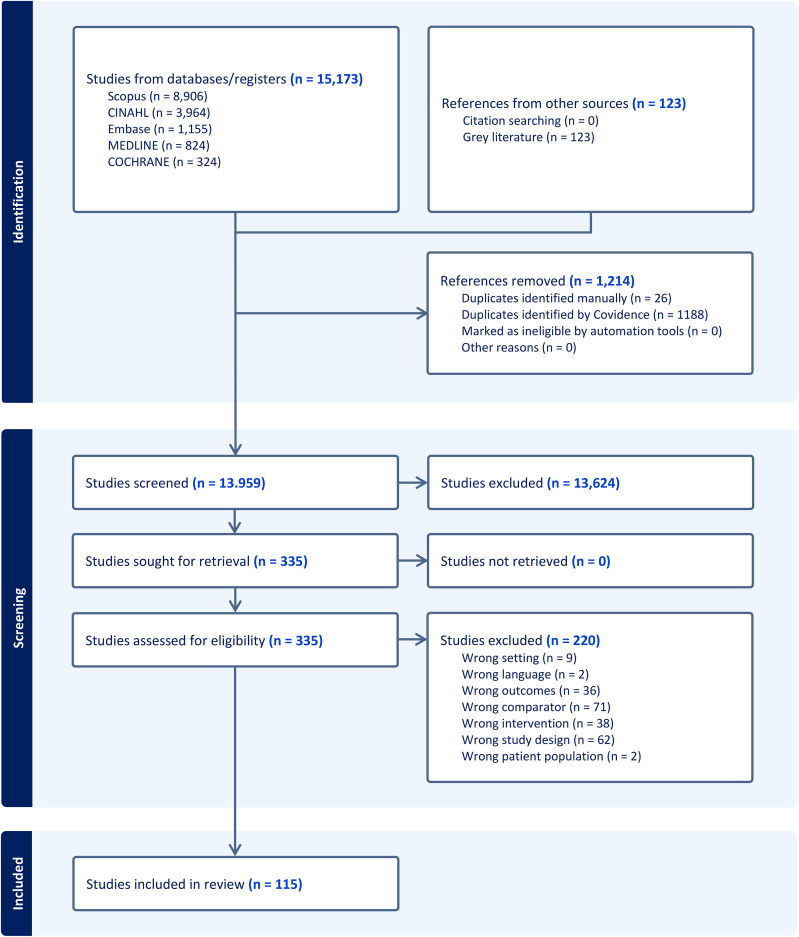
Study selection flow diagram.

After the initial database search and duplicate removal, the investigators screened 13,959 studies, assessed 335 studies for eligibility and excluded 220 studies. Ultimately, the investigators included 115 studies in the review. The non-indexed/grey literature search yielded 123 references that together with the references found from backward citation searching, however, were all excluded, since they did not meet the inclusion criteria or were found to be duplicates. The screening, assessment, and inclusion process is available in the PRISMA flow diagram (Fig 1) and databases searches are also available S1 File.

### Characteristics of study subjects

The study designs were retrospective observational studies (86.1%), prospective observational studies (11.3%), randomized controlled trials (1.7%), and interrupted time series (0.9%). The publication year ranged from 1979 to 2024. The studies originated from all parts of the world apart from Africa, including Europe (53.0%), Asia (16.5%), North America (13.0%), Australia/Oceania (11.3%), the Middle East (3.5%), and South America (2.6%).

The related prehospital organizations ranged from general EMS (35.7%); ground EMS (38.3%); helicopter EMS (7.0%); physician-manned mobile emergency care unit (5.2%); paramedic-manned EMS (2.6%), to combined organizations in mixed EMS (11.3%). The study settings comprised rural (1.7%); suburban (0.9%); urban (12.2%): metropolitan (14.8%) and a combination of those in mixed settings (70.4%). The diagnoses investigated comprised out-of-hospital cardiac arrest (75.7%), trauma (11.3%), miscellaneous/unspecified (7.0%), traumatic out-of-hospital cardiac arrest (3.5%), and drowning (2.6%) (Table 1).

**Table 1:** Study characteristics.

Study characteristics	Studies, n (%) (Total = 115)
**Study design**	
Retrospective observational study	99 (86.1)
Prospective observational study	13 (11.3)
Randomized controlled trial	2 (1.7)
Interrupted time series	1 (0.9)
**Publication year**	
1970–1979	1 (0.9)
1980–1989	1 (0.9)
1990–1999	5 (4.3)
2000–2009	23 (20.0)
2010–2019	52 (45.2)
2020–2024	33 (28.7)
**Geographical location**	
Europe	61 (53.0)
North America	15 (13.0)
South America	3 (2.6)
Middle East	4 (3.5)
Asia	19 (16.5)
Australia/Oceania	13 (11.3)
**Organization**	
EMS in general*	41 (35.7)
Ground EMS only	44 (38.3)
Helicopter EMS only	8 (7.0)
Mobile emergency care unit only	6 (5.2)
Paramedic-manned EMS only	3 (2.6)
Mixed**	13 (11.3)
**Setting**	
Metropolitan	17 (14.8)
Rural	2 (1.7)
Suburban	1 (0.9)
Urban	14 (12.2)
Mixed	81 (70.4)
**Diagnoses investigated in study**	
Drowning	3 (2.6)
Miscellaneous*	8 (7.0)
Out-of-hospital cardiac arrest	87 (75.7)
Trauma	13 (11.3)
Traumatic out-of-hospital cardiac arrest	4 (3.5)

Legend: EMS: Emergency medical services; *Unspecified in the included study; **E.g., Ground EMS/Helicopter EMS.

The included studies comprised 691,056 patients in total, and the sample size range was 64–182,895 patients, with a median (IQR) of 1,064 (394–3,334). Notably, two large studies accounted for 35% of all patients, with sample sizes of 59,926 and 182,895. Prior to formal analysis, each study was weighted equally, based on between-study heterogeneity and very diverse reporting of both patient-centered and EMS-centered outcomes.

### Main results

#### Patient-centered outcomes.

Survival rate, calculated from 30-day survival (n = 31 studies, 27.0%) or survival to discharge (n = 68 studies, 59.1%) in a composite outcome, was assessed in n = 99 (86.1%) studies and ranged between 0.0% and 98.6%. This wide variability reflects differences in study populations, EMS practices, and healthcare systems. The remaining studies reported alternate outcomes, such as return of spontaneous circulation in n = 23 (20.0%), Cerebral Performance Category in n = 12 (10.4%), 24-hour survival in n = 6 (5.2%) and a variety of other outcome measures S1 File.

For the organizational domain (n = 105 studies, 91.3%), median survival rate ranged between 11.2% (IQR 5.2; 20.4) in ground EMS only and 86.0% (IQR 31.2; 91.1) in helicopter EMS only. In the setting domain (115 studies, 100.0%), survival rate varied between 4.7% (IQR 1.5; 9.8) in metropolitan area and 98.6% in suburban areas. For diagnoses (115 studies, 100.0%), survival rates were between 7.5% (IQR 4.5; 10.9) in traumatic out-of-hospital cardiac arrest and 92.2% (IQR 86.0; 95.6) in trauma. The survival rates in geographical location (115 studies, 100.0%) ranged from 3.9% (IQR 2.0; 44.0) in South America to 31.0% (IQR 11.1; 93.6) in North America. The overall median survival rate in the included studies was 11.5% (IQR 5.2; 25.8). Please refer to Supplemental material S1 File for a full overview of survival rates related to study characteristics.

#### EMS-centered measures.

Response time was reported in mean with standard deviation or median with inter-quartile range in n = 108 (93.9%) studies, ranging from 1.10 to 48.62 minutes. On-scene time and call-to arrival time were also reported in n = 15 (13.0%) and in n = 10 (8.7%) of the studies, respectively.

#### Pre-defined data extraction results.

The five pre-defined domains with items were accounted for in varying degrees by the included studies: Geo-political setting in 68.0%, EMS characteristics in 65.0%, EMS response characteristics in 30.0%, vehicle activation characteristics in just 4.8% and response time characteristics in 53.6. The overall rate of domains with items accounted for was 46.7% (Table 2) S1 File.

**Table 2:** Study findings from the included literature.

Study findings	Domains and items answered % (n/total items)
**Data extraction domains and items**	
Geo-political setting • Basic information on area, population, accessibility, other	**68.0%** (313/460)
Emergency medical services characteristics • Population covered, catchment area, structure, other	**65.0%** (290/460)
Emergency medical services response • Type of dispatch system, criteria, EMDC structure, other	**30.0%** (138/460)
Activation characteristics • Limitations in activation, response time requirements, Fines for late response, other	**4.8%** (22/460)
Response time characteristics • Definitions, measurements, storage, information use, outcome measures, mortality correlation, outcome correlation diagnoses, correlation to distance & capacity at receiving hospital	**53.6%** (739/1,380)
**Overall**	**46.7%** (1,502/3,220)
**Quality appraisal domains and items**	
Internal validity • Author employed in EMS, where/how was data obtained, conflicts of interest, ethics committee approval	**74.4%** (428/575)
External validity • Structure description, dispatch system, response time characteristics, Missing data, limitations, study design, outcomes described	**73.3%** (590/805)
**Overall**	**73.8%** (1,018/1,380)

Legend: EMDC: Emergency medical dispatch center.

A summary of the results for each outcome category, the results of the individual studies and, finally, a summary of results and the references of the included studies are presented in S1 File, which also provides a narrative interpretation of each of the individual studies.

#### Association between response time and survival.

An association between response time and survival was assessed in n = 47 (40.9%) studies. The authors used very diverse methods such as differently adjusted odds ratios, which were based on both continuous and dichotomized response times. Further, some odds ratios for survival were given with or without good cerebral or functional outcome. Therefore, the odds ratios could not be pooled or combined in an overall meta-analysis as per Cochrane guidelines [9].

In meta-analyses of the sub-groups drowning (n = 2 studies, 570 patients), traumatic out-of-hospital cardiac arrest (n = 2 studies, 8,514 patients), miscellaneous (n = 3 studies, 235,736 patients), trauma (n = 5 studies, 22,400 patients) and out-of-hospital cardiac arrest (n = 27 studies, 140,757 patients), the investigators made forest plots based on the studies providing comparable, adjusted odds ratios for the association between response time and survival. The investigators calculated the individual study weights based on confidence intervals. In a Random-effects model, the odds ratios for the association between response time and survival ranged from 0.87 95% CI [0.68; 1.13] in drowning to 1.21, 95% CI [0.80; 1.84] in trauma. Heterogeneity I^2^ varied between 16.40% (Traumatic out-of-hospital cardiac arrest) and 99.59% (out-of-hospital cardiac arrest) in the subgroup meta-analyses of the eligible studies of the included literature (Table 3). Please refer to Supplemental material S1 File for forest plots.

**Table 3:** Meta-analysis of subgroups.

Domains	Odds ratio, 95% CI	Heterogeneity,%	Studies, n	Patients in studies, n
Drowning	0.87 [0.68; 1.13]	68.73	2	570
Traumatic OHCA	1.00 [0.97; 1.02]	16.40	2	8,514
Miscellaneous	0.94 [0.83; 1.07]	98.93	3	235,736
Trauma	1.21 [0.80; 1.84]	74.97	5	22,400
OHCA	1.02 [0.86; 1.21]	99.59	27	140,757
Total			39	406,977

Legend: CI: Confidence interval; OHCA: Out-of-hospital cardiac arrest.

### Study quality

For internal and external validity, 74.4% and 73.3% of the predefined domains and items were accounted for, respectively (Table 2) (S1 File). As per protocol, the investigators assessed risk of bias across studies in three groups of: 1) treatment by ambulance and/or helicopter services with recorded response time, 2) patient-centered outcomes, and 3) EMS-centered measures, respectively. A serious risk of bias was found in observational studies (n = 113) but not in the randomized controlled studies (n = 2). The overall quality was assessed using the GRADE [21] methodology as per protocol. The quality of the randomized controlled trials (n = 2) was moderate, whereas the quality of the observational studies (n = 113) was very low. The investigators down-rated quality as per GRADE guidelines, i.e., rated quality depending on the number of risk of bias in the QUADAS-2 [24] domains *patient selection* and *reference standard*. Please refer to Table 4 for details and comments to the quality of evidence.

**Table 4:** Overall quality of evidence (GRADE).

Overall quality of evidence (GRADE)							
Outcome	Study design	Risk of bias	Inconsistency	Indirectness	Imprecision	Publication bias	Overall quality of evidence
Exposure to response time, EMS treatment (n = 115)	113 Observational	Serious (a)	Not serious	Not serious (c)	Serious (d)	Undetected	Very low ⊕OOO
	2 RCT	Not serious (b)	Not serious	Not serious (c)	Serious (d)	Undetected	Moderate ⊕⊕⊕O
Patient-centered outcome measures (n = 115)	113 Observational	Serious (a)	Serious (e)	Not serious (c)	Not serious	Undetected	Very low ⊕OOO
	2 RCT	Not serious (b)	Serious (e)	Not serious (c)	Not serious	Undetected	Moderate ⊕⊕⊕O
EMS-related measures (n = 115)	113 Observational	Serious (a)	Serious (e)	Not serious (c)	Not serious	Undetected	Very low ⊕OOO
	2 RCT	Not serious (b)	Serious (e)	Not serious (c)	Not serious	Undetected	Moderate ⊕⊕⊕O

Abbreviations: Grading of Recommendations Assessment, Development, and Evaluation (GRADE), Randomized Controlled Trials (RCT).

From: Schunemann HJ, Brennan S, Akl EA, Hultcrantz M, Alonso Coello P, Xia J, et al. The development methods of official GRADE articles and requirements for claiming the use of GRADE – a statement by the GRADE Guidance Group. J Clin Epidemiol. 2023 May 19;159:79-84. https://doi.org/10.1016/j.jclinepi.2023.05.010.

(a) Observational studies were overall judged to be of high risk of bias due to a lack of contemporaneous comparison groups with a potential effect on the reported results.

(b) Randomized controlled trials were overall judged to be of low risk of bias, due to randomization and blinding.

(c) Measurement of hard endpoints, i.e., survival to discharge, 30-day mortality.

(d) All of the included studies reported on exposure to response time, but in very diverse settings and organizations.

(e) Inconsistencies in outcome measures were substantive. This could be due to differences in settings and organizations. The investigators observed large variations in reported results.

A summary of findings assessing the risk of biases related to patient selection, the index test, and the reference standard is presented in Table 5. Patient selection was unclear or at high risk in n = 113 (98.3%). In the Index test comprising dispatch and interventions performed by EMS, there was a low risk in n = 115 (100.0%). Accordingly, the overall certainty of evidence as per GRADE was *very low*.

**Table 5:** Summary of findings.

Review question: What is the association between overall emergency medical services unit response time and patient survival?				
*Population:* All patients treated by ambulance and/or helicopter services				
*Setting:* Rural, suburban, urban, metropolitan, mixed				
*Study design:* Prospective observational study, retrospective observational study, interrupted time series, randomized controlled trials				
*Index test:* Response time, interventions performed by EMS personnel, treatment by ambulance/helicopter services				
*Target condition:* Condition triaged by EMDC to be in need of ambulance/helicopter services treatment				
*Reference standards:* Utstein concept, reference standards if applicable,				
*Limitations in the evidence:*				
High risk of bias in the included studies (98.3%), apart from randomized controlled studies (1.7%), driven primarily by the nature of observational studies; lack of reference standards beyond Utstein concept. • Patient selection: Unclear or high risk of bias in n = 113 (98.3%) studies, high concern of applicability in n = 113 (98.3%) studies • Index test: Low risk of bias in n = 115 (100.0%) studies; low concern of applicability in n = 115 (100.0%) studies • Reference standard: High or unclear risk of bias n = 104 (90.4%), high or unclear concern of applicability in n = 113 (98.3%) studies. Utstein reference standard in n = 11 (9.6%) with no risk of bias. • Flow and timing: High or unclear risk of bias in n = 113 (98.3%) studies				
Overall assessment: Most studies (98%) were at risk of bias and had concerns regarding applicability				
**Findings**				
**Number of studies** **(Participants)**	**Patient selection Unclear/high risk (%)**	**Index test** **Low risk of bias (%)**	**Reference standard** **High/unclear risk (%)**	**GRADE certainty of** Evidence*
115 (691,056)	113 (98.3)	115 (100.0)	104 (90.4)	⊕OOO Very low

Legend: EMDC: Emergency medical dispatch center; EMS: Emergency medical services; CI: Confidence interval.

* GRADE methodology was applied for the assessment of the certainty of evidence: Risk of bias was rated serious, because 98% of the studies had a high risk of bias in one QUADAS-2 domain; indirectness was rated not serious; inconsistency was rated not serious, because the substantive heterogeneity (I^2 ^= 99.93%) could be explained by the patient variability/spectrum; imprecision was rated not serious for sensitivity and not serious for specificity. The investigators detected no publication bias.

GRADE Working Group grades of evidence

- **High quality:** The investigators are very confident that the true effect lies close to that of the estimate of the effect
- **Moderate quality:** The investigators are moderately confident in the effect estimate: The true effect is likely to be close to the estimate of the effect, but there is a possibility that it is substantially different
- **Low quality:** Our confidence in the effect estimate is limited: The true effect may be substantially different from the estimate of the effect
- **Very Low quality:** Our confidence in the effect estimate is limited: The true effect may be substantially different from the estimate of the effect

## Discussion

The main finding of this systematic review and meta-analysis is that with very low certainty, there is a lack of evidence to infer an association between the EMS response time and survival. To our best knowledge, this systematic review and meta-analysis of 115 studies comprising more than half a million patients from most parts of the world, except Africa that do not have EMS in large parts of the continent [25], provides an almost complete picture of the current body of evidence. Dominated by observational studies, the certainty of evidence was very low, and further, the substantive heterogeneity of the underlying diagnoses, age groups and the predominance of traumatic cardiac arrest and out-of-hospital cardiac arrest weakens the signal of the review. In a meta-analysis of sub-groups, there was a positive correlation in selected, small populations, whereas in the majority of patient populations, heterogeneity hindered conclusions. Therefore, no actionable conclusions can be made and the review has demonstrated substantive research and knowledge gaps. It is very important to emphasize that the low quality of the evidence weakens the signal presented here.

This systematic review and meta-analysis included just two randomized controlled trials. Conducting randomized controlled studies in the prehospital arena is still considered difficult. However, several studies are currently being conducted worldwide. Therefore, the scientific quality of prehospital research is on the rise [26]. One study [27] addresses the inherent methodological problems in prehospital research such as inappropriate research methods. Solutions include agreement on uniform datasets, classifications and definitions, and the introduction of risk adjustment measurements to mitigate confounders. Due to patient heterogeneity, the risk of incomplete data, and the possibility of inconsistency in the prehospital care provided, registry studies may be difficult to interpret. In a systematic mapping review of twenty randomized controlled trials in prehospital trauma [28], the authors found that evidence is sparse and study designs and reporting is poor. Furthermore, the outcome following prehospital events may be influenced by the in-hospital treatment with variations in and between hospitals. Thus, in an expert perspective comment on research questions in pre-hospital trauma care research [29], the author finds mortality to be an “insensitive outcome measure of an isolated pre-hospital intervention”, since for instance trauma patients undergo many interventions during their hospital stay that may affect outcome.

Key performance indicators may be defined as “indicators used to monitor and evaluate critical areas of clinical and support functions that influence patient outcome”. In a systematic literature review [30] followed by a three-round Delphi process, an expert group developed 101 indicators, divided into structure, process and outcomes. Similar processes to develop indicators were conducted for an EMS benefit score [31] and in a study on quality indicators in prehospital advanced airway management [32]. Another study described the lack of consensus regarding which quality indicators to use [33], posing a risk of a narrow perspective in quality assessment in EMS. The same group published [34] the results from an expert panel that developed 26 quality indicators for EMS. The use of expert panels in Delphi processes [35,36] to synthesize existing knowledge and issue recommendations is not widespread [37] and has been subjected to criticism [38] for low reliability and should optimally follow a registered protocol [39].

The heterogeneity of the available literature poses a major challenge in attempting to understand performance indicators of interest to improve patient outcome. In this review, the investigators detected a substantive variability in treatment and outcome classifications and often, the included studies only described outcome data instead of reporting quantitative results, echoing the diversity in EMS organizations. This is an indication of a lack of uniform reporting guidelines that the Utstein [40] concept methodology encompasses. The development of formula of survival [41] reflects the ambitions of the Utstein format. In this review, several of the included studies did use the Utstein reporting framework.

In this review, the preferred reported measure related to EMS performance was response time [1–4]. The use of response time as a performance indicator has been questioned extensively [1,4,42,43]. The authors of these studies have not been able to identify a significant association between response time and survival. In a recent joint statement [6], the authors suggest a modernization of the assessment of the EMS performance by applying several domains with key performance indicators to be measured over time, and benchmarked against comparable EMS systems. In contrast to response time, the patient perspective is also suggested as an important aspect in terms of satisfaction and equal access to EMS provided care.

This review has demonstrated substantive research and hence knowledge gaps despite extensive research in the EMS field. However, the methodology of the literature is weak and dominated by observational studies, characterized by the inability to address important biases such as confounding by indication [7]. A study describes benchmarking in EMS [44], suggesting that the benefit conferred by EMS interventions is presented in the *numbers-needed-to- treat* format.

Based on low certainty of evidence, response time association with patient survival remains ambiguous and may not be of major importance for the vast majority of EMS missions. Therefore, future research in this field should embrace more rigorous and precise study designs, and perhaps limiting analysis to comparable countries and types of transport may produce more context-specific conclusions.

Based on the influence of the heterogeneity of the included studies to the review signal, the investigators suggest that future studies should focus on fixed variables and endpoints, such as short-term mortality, e.g., 48 hour mortality. There should be a clear distinction between the levels of competencies within the prehospital service, i.e., emergency medical technicians, paramedics and prehospital physicians. Further, the mode of transportation in ground-based units or rotor wing aircraft. Finally, studies should describe clearly defined study populations such as cardiac arrest, respiratory insufficiency, general trauma and neurotrauma.

In retrospect, the authors could have chosen narrower inclusion criteria for this systematic review, which would have produced a further focused review. Further, the investigators could have imposed time and language limitations as well, whereas limiting study designs to exclude conference abstracts or only include studies presenting uniformly calculated odds ratios for the association between response time and survival, would have restricted the inclusion of studies further.

### Strengths and limitations

The strengths of this systematic review are protocol adherence [13,14] and the consistent use of validated guidelines [9,10,13] in the design of the review and the reporting of the study findings. Any protocol amendment or deviation was reported in the relevant portal and the number of amendments and deviations was limited, adding to the generalizability and reproducibility of the review. In this review, a comprehensive search strategy is likely to have captured all relevant studies; i.e., the substantive number of studies included in the review and a very large number of patients may have led to greater precision in summary estimates. Furthermore, snowballing reference lists of the included literature did not render additional studies, adding to an almost complete picture of the relevant literature on the topic.

The review has several limitations as well. There is a skewness in the included literature towards cardiac arrest and trauma, which do not represent the majority of EMS missions. Therefore, the lack of data surrounding the vast majority of patients hinders general conclusions based on the review findings. The low-quality evidence of the included studies cannot address confounding by indication that is inherent in observational studies.

Heterogeneity in the meta-analysis of out-of-hospital cardiac arrest and drowning studies was substantive, calling for outmost caution in the interpretation of the effect investigated. In the Cochrane Handbook [9], statistical heterogeneity refers to variability in the intervention effects being evaluated in the studies. When heterogeneity is present, this may partly be due to an insufficient number of included studies. In the exploration of heterogeneity, obvious outliers were not identified, and therefore, the exclusion of outlying studies was not an option, which could introduce bias. Further, the use of inverse variance in the weighing of studies in the meta-analyses addresses and serves to incorporate considerable heterogeneity but does not allow for actionable conclusions based on interpretation of the findings.

Finally, the use of composite outcome may be problematic [45,46], owing to the fact that components may be unreasonably combined, inconsistently defined, and inadequately reported. The investigators believe that was not the case in this systematic review, as the selected outcome components are well defined, reasonably combined and adequately reported.

## Conclusion

This systematic review and meta-analysis found lack of evidence to infer an association between the EMS response time and patient survival, with very low certainty of evidence. In a meta-analysis of sub-groups, there was a positive correlation in selected, small populations. Accordingly, no actionable conclusions can be made and therefore, the review has demonstrated substantive research and knowledge gaps. Response time association with patient survival remains ambiguous and may not be of major importance for the vast majority of EMS missions.

## Supporting information

S1 File Supporting information. Zenodo Repository (https://zenodo.org/records/15076410).
(DOCX)

S1 Table Irrelevant studies from the titles and abstract screening. (XLSX)

S2 Table Included studies from full-text. (XLSX)

S3 Table Excluded studies from full-text. (XLSX)

S4 Table Data extracted from the primary research sources. (DOCX)

S5 Table Data or supporting information obtained from another source. (DOCX)

## Abbreviations

EMS: emergency medical services
PRISMA: Preferred Reporting Items for Systematic Reviews and Meta-analyses
GRADE: Grading of Recommendations Assessment, Development, and Evaluation
IQR: Inter quartile range.
