# Supplementary material for: Association of ambulance and helicopter response times with patient survival: A systematic literature review and meta-analysis
Source: PLoS One. 2025 Nov 17;20(11):e0335665. doi: 10.1371/journal.pone.0335665 (PMC12622838; doi:10.1371/journal.pone.0335665)
Supplement: S1 File — Zenodo Repository (https://zenodo.org/records/15076410). (DOCX) [file pone.0335665.s001.docx]

Peter Martin Hansen, Martine Siw Nielsen, Marius Rehn, Annmarie Touborg Lassen, Anders Perner, Søren Mikkelsen, Anne Craveiro Brøchner

**Supplemental digital content**

This supplement has been provided by the authors to provide the readers with additional information about the review and meta-analysis.

**Table of contents**

**Supplemental methods**……………………………………………………………………………………………………………………………..……2

Supplemental Text 1: Search strategies of electronic databases………………………………………….………………………2

Supplemental Text 2: Search strategies of non-indexed and grey literature…………………………………………….….7

Supplemental Text 3: Data extraction template domains and items………………………..………………………………….8

Supplemental Text 4: Quality appraisal template domains and items…………………………………………..……………..9

Supplemental Text 5: Deviations from study protocol…………………………………………………….…………………………10

Supplemental Text 6: Generic letter to authors…………………………………….…………………….….…………………………10

**Supplemental results**……………………………………………………………………………………………………………………………….……11

Supplemental Table 1: Author contact………………………………………………………………………………………………………11

Supplemental Table 2: Data extraction………………………………………………………………………………………………………13

Supplemental Table 3: Quality appraisal……………………………………………………………………………………………………19

Supplemental Table 4: Reported outcome measures categorized…………………………………………………………….23

Supplemental Table 5: Summary of results for each outcome category…………………………………………………….24

Supplemental Table 6: Overview of study characteristics of the included literature ………...……….…………….27

Supplemental Table 7: Results of the individual studies………………………………………… …………………………………38

Supplemental Table 8: Outcomes and study periods………………………….. ………………..………………………………….49

Supplemental Table 9: Survival rates related to study characteristics from the included literature……..……54

Supplemental Table 10: Narrative interpretation of the included studies ………….………………..……………………55

Supplemental Figure 1: Sub-group forest plots………………………………………………………………………………………….68

**PRISMA 2000 checklist**………………………………………………………………………………………………………………………………….71

**References of the included literature**…………..……………………………………………………………………………………………….74

**Supplemental methods**

**Search strategies of electronic databases**

EMBASE (Ovid) 1974 to date of search (28 September 2024)

**# Search ____**

**1** exp air medical transport/

**2** (air medical transport* or air ambulance* or aeromedical transfer or helicopter emergency medical service* or EMS helicopter or helicopter EMS).mp. [mp=title, abstract, heading word, drug trade name, original title, device manufacturer, drug manufacturer, device trade name, keyword heading word, floating subheading word, candidate term word]

**3** 1 or 2

**4** reaction time/

**5** (reaction time* or Response time* or Reaction latenc* or Response latenc*).mp. [mp=title, abstract, heading word, drug trade name, original title, device manufacturer, drug manufacturer, device trade name, keyword heading word, floating subheading word, candidate term word]

**6** 4 or 5

**7** 3 and 6

**8** ambulance response time/

**9** (Ambulance response time* or Ambulance reaction time* or ambulance service response time* or response time by ambulance service* or response times of ambulance*).mp. [mp=title, abstract, heading word, drug trade name, original title, device manufacturer, drug manufacturer, device trade name, keyword heading word, floating subheading word, candidate term word]

**10** 8 or 9

**11** 7 or 10

**12**  exp ambulance/

**13** (ambulance* or rapid response unit* or rapid response car* or mobile emergency care unit* or mobile intensive care unit*).mp. [mp=title, abstract, heading word, drug trade name, original title, device manufacturer, drug manufacturer, device trade name, keyword heading word, floating subheading word, candidate term word]

**14** 12 or 13

**15** 6 and 14

MEDLINE (Ovid) 1946 to date of search (28 September 2024)

**# Search ____**

**1** exp Air ambulance/

**2** (air medical transport* or air ambulance* or aeromedical transfer or helicopter emergency medical service* or EMS helicopter or helicopter EMS).mp. [mp=title, book title, abstract, original title, name of substance word, subject heading word, floating sub-heading word, keyword heading word, organism supplementary concept word, protocol supplementary concept word, rare disease supplementary concept word, unique identifier, synonyms, population supplementary concept word, anatomy supplementary concept word]

**3** 1 or 2

4 reaction time/

**5** (reaction time* or Response time* or Reaction latenc* or Response latenc*).mp. [mp=title, book title, abstract, original title, name of substance word, subject heading word, floating sub-heading word, keyword heading word, organism supplementary concept word, protocol supplementary concept word, rare disease supplementary concept word, unique identifier, synonyms, population supplementary concept word, anatomy supplementary concept word]

**6** 4 or 5

**7** 3 and 6

**8** ambulance response time/

**9** (Ambulance response time* or Ambulance reaction time* or ambulance service response time* or response time by ambulance service* or response times of ambulance*).mp. [mp=title, book title, abstract, original title, name of substance word, subject heading word, floating sub-heading word, keyword heading word, organism supplementary concept word, protocol supplementary concept word, rare disease supplementary concept word, unique identifier, synonyms, population supplementary concept word, anatomy supplementary concept word]

**10** 8 or 9

**11** 7 or 10

**12** exp ambulance/

**13** (ambulance* or rapid response unit* or rapid response car* or mobile emergency care unit* or mobile intensive care unit*).mp. [mp=title, book title, abstract, original title, name of substance word, subject heading word, floating sub-heading word, keyword heading word, organism supplementary concept word, protocol supplementary concept word, rare disease supplementary concept word, unique identifier, synonyms, population supplementary concept word, anatomy supplementary concept word]

**14** 12 or 13

**15** 6 and 14

Cochrane Library search (28 September 2024)

**# Search ____**

**1** MeSH descriptor: [Air Ambulances] explode all trees

**2** MeSH descriptor: [Ambulances] explode all trees

**3** MeSH descriptor: [Reaction Time] explode all trees

**4** MeSH descriptor: [Time-to-Treatment] explode all trees

**5** MeSH descriptor: [Emergency Medical Services] explode all trees

**6** (rapid response unit):ti,ab,kw (Word variations have been searched)

**7** (mobile emergency care unit):ti,ab,kw (Word variations have been searched)

**8** (hems):ti,ab,kw (Word variations have been searched)

**9** (air retrieval service):ti,ab,kw (Word variations have been searched)

**10** (ambulance time):ti,ab,kw (Word variations have been searched)

**11** (helicopter time):ti,ab,kw (Word variations have been searched)

**12** (dispatch time):ti,ab,kw (Word variations have been searched)

**13** (#1 or #2 or #6 or #7 or #8 or #9)

**14** (#3 or #4 or #5 or #10 or #11 or #12)

**15** (#13 and #14)

CINAHL (EBSCO) search (28 September 2024)

**# Search phrase ____**

**1** aeromedical transport*

**2** helicopter transport*

**4** helicopter emergency medical services*

**5** ambulance transport*

**6** ambulance transfer*

**7** mobile emergency team*

**8** reaction time or response time*

Free text search:

(aeromedical transport* OR helicopter transport OR helicopter emergency medical services* AND ambulance transport OR ambulance transfer OR mobile emergency team AND (reaction time or response time))

Embase Classic+Embase 1947 to date of search (28 September 2024)

# **Searches**  ________________

**1** exp air medical transport/

**2** (air medical transport* or air ambulance* or aeromedical transfer or helicopter emergency medical service* or EMS helicopter or helicopter EMS).mp. [mp=title, abstract, heading word, drug trade name, original title, device manufacturer, drug manufacturer, device trade name, keyword heading word, floating subheading word, candidate term word]

**3** 1 or 2

**4** reaction time/

**5** (reaction time* or Response time* or Reaction latenc* or Response latenc*).mp. [mp=title, abstract, heading word, drug trade name, original title, device manufacturer, drug manufacturer, device trade name, keyword heading word, floating subheading word, candidate term word]

**6** 4 or 5

**7** 3 and 6

**8** ambulance response time/

**9** (Ambulance response time* or Ambulance reaction time* or ambulance service response time* or response time by ambulance service* or response times of ambulance*).mp. [mp=title, abstract, heading word, drug trade name, original title, device manufacturer, drug manufacturer, device trade name, keyword heading word, floating subheading word, candidate term word]

**10** 8 or 9

**11** 7 or 10

**12**  exp ambulance/

**13** (ambulance* or rapid response unit* or rapid response car* or mobile emergency care unit* or mobile intensive care unit*).mp. [mp=title, abstract, heading word, drug trade name, original title, device manufacturer, drug manufacturer, device trade name, keyword heading word, floating subheading word, candidate term word]

**14** 12 or 13

**15** 6 and 14

**Search strategies of non-indexed and grey literature**

We also included non-indexed and grey literature and searched the following databases:

- Web of Science searched 24 September 2024
- Google Scholar searched 24 September 2024
- <http://www.ndltd.org> searched 25 September 2024
- <https://www.dart-europe.org/basic-search.php> searched 24 September 2024
- <http://www.opengrey.eu/> searched 25 September 2024
- <https://www-base-search-net.ezproxy.uis.no/> searched 24 September 2024
- <https://oatd.org/> searched 25 September 2024

**Data extraction template domains and items**

Does the included literature report the following? (Yes, no)

**Data on geo-political setting**

- Basic information on area?
- Basic information on population?
- Basic information on accessibility in the region, e.g., rural, city, etc.?
- Other relevant data on area?

**Emergency medical services (EMS) characteristics**

- Population covered by EMS?
- EMS catchment area?
- EMS structure?
- Other EMS characteristics?

**EMS response characteristics**

- Type of EMS dispatch system?
- Description of dispatch criteria
- Structure of emergency medical dispatch center (EMDC) in the area?
- Other EMS response characteristics?

**Ambulance and helicopter activation characteristics**

- Limitations in activation, e.g., weather, conflict of simultaneity etc.
- Response time requirements?
- Fines for late response?
- Other response times characteristics?

**Ambulance and helicopter response times characteristics**

- How was response time data defined?
- How was response time data measured?
- How was response time data stored?
- How was response time data information used?
- What were the primary and secondary outcome measures?
- Was response time correlated to mortality?
- Was response time correlated to outcome, adverse events etc.?
- Was response time correlated to diagnoses?
- Was response time correlated to distance to patient?
- Was response time correlated to distance to receiving hospital?
- Was response time correlated to capacity at receiving hospital?
- Other response time data described?

**Quality appraisal template domains and items**

Does the included literature report the following? (Yes, no)

**Internal validity**

- Is the author employed in the EMS organization?
- Does the literature provide reference to where data were obtained?
- Does the literature provide reference to how data were obtained?
- Do the authors have conflicts of interest?
- Has an ethics committee approved the study?

**External validity**

- Is the EMS structure clearly described?
- Is the type of EMS dispatch system clearly described?
- Are the response time characteristics clearly described?
- Are there indications of missing data?
- Are other limitations discussed?
- Is the study design clearly explained?
- Are the primary and secondary outcomes clearly described?

**Deviations from the study protocol**

**Protocol method Deviation from protocol Justification**

**_______________________________________________________________________________________**

We planned to include the Prior to database search, we decided We sought to include references SweMed database in our to discard the SweMed database from from updated and relevant data- search strategy. our search strategy. bases. SweMed had been discontinued

_______________________________________________________________________________________

We planned to apply Trial After the database search, we decided Only two randomized controlled Sequential Analysis in the to abandon Trial Sequential Analysis in trials in the review.

Systematic review. the synthesis of results.

_______________________________________________________________________________________

**Generic letter to authors**

Dear Madam/Sir.

My name is Peter Martin Hansen. I am a Danish PhD student and prehospital physician conducting a systematic review and meta-analysis on the association between ambulance and helicopter response time and survival and outcome.

The protocol is published here:

<https://onlinelibrary.wiley.com/doi/10.1111/aas.14339>

We included your study:

**XXX**

in the review.

We address you to ask if you have unpublished data from your study that describes the association between response time and outcome. That is, if you have assessed the association statistically, e.g., by way of Odds Ratio or a similar method.

Thank you for your time. Your contribution to our review is valuable.

Best regards

Dr. Peter Martin Hansen, MD, MSc, PhD stude

**Supplemental Table 1: Author contact.**

| **Author/year** | **Author contacted** | **Author responded** | **Additional data** | **No response** |
| --- | --- | --- | --- | --- |
| **Afzali 2013^2^** | X | X |  |  |
| **Ahmoudi 2022^3^** | * |  |  | X |
| **Aziz 2020^7^** | X | X |  |  |
| **Berge 2005^10^** | X | X |  |  |
| **Biewener 2000^12^** | * |  |  | X |
| **Bjorkmann 2020^13^** | X | X | X |  |
| **Blaengsdottir 1994^16^** | * |  |  | X |
| **Bossers 2021^18^** | X |  |  | X |
| **Brison 1992^20^** | * |  |  | X |
| **Brown 2019^21^** | * |  |  | X |
| **Cardoso 2014^25^** | X |  |  | X |
| **Chesters 2015^28^** | X |  |  | X |
| **Deasy 2012^30^** | X |  |  | X |
| **Einarsson 1989^35^** | * |  |  | X |
| **Eisenburger 2001^36^** | * |  |  | X |
| **Ong 2003^37^** | X |  |  | X |
| **Finney 2023^39^** | X | X |  |  |
| **Fothergill 2021^40^** | X | X |  |  |
| **Fraga-Sastrias 2009^41^** | * |  |  | X |
| **Gnesin 2021^42^** | X | X |  |  |
| **Goh 2018^43^** | * |  |  | X |
| **Gregers 2021^45^** | X | X | X |  |
| **Grmec 2009^46^** | * |  |  | X |
| **Gunaga 2020^47^** | * |  |  | X |
| **Hayes 2010^49^** | * |  |  | X |
| **Hillis 1993^53^** | * |  |  | X |
| **Huabbangyang 2021^55^** | X |  |  | X |
| **Jennings 2006^57^** | X | X |  |  |
| **Kennedy 2023^59^** | X |  |  | X |
| **Kentsch 2000^60^** | * |  |  | X |
| **Klosiewicz 2017^62^** | X | X | X |  |
| **Lee 2013^64^** | X |  |  | X |
| **Leung 2001^65^** | X |  |  | X |
| **Lin 2014^67^** | * |  |  |  |
| **Little 2020^68^** | X |  |  | X |
| **Lyon 2013^70^** | X |  |  | X |
| **Meyer 2001^74^** | X |  |  | X |
| **Mogensen 2015^79^** | X |  |  | X |
| **Morrisey 1996^80^** | * |  |  | X |
| **Nichol 2016^84^** | X | X |  |  |
| **Nielsen 2022^85^** | X | X |  |  |
| **Park 2017^87^** | X |  |  | X |
| **Renkiewicz 2014^94^** | X |  |  | X |
| **Schinnerl 1990^95^** | * |  |  | X |
| **Semensato 2011^96^** | * |  |  | X |
| **Shah 2010^97^** | X |  |  | X |
| **Shepherd 2008^98^** | * |  |  | X |
| **Sigursson 2000^99^** | * |  |  | X |
| **Stromsoe 2015^103^** | X | X |  |  |
| **Stromsoe 2011^104^** | X | X |  |  |
| **TerAvest 2019^107^** | X |  |  | X |
| **Thompson 2017^108^** | * |  |  | X |
| **Trenkler 2012^109^** | * |  |  | X |
| **Wai 2005^111^** | * |  |  | X |
| * : No valid electronic mail address; electronic mail bounced back | | |  |  |

**Supplemental table 2. Data extraction**

|  | GEO-POLITICAL | | | | EMS CHARACTERISTICS | | | | EMS RESPOSONSE | | | | ACTIVATION | | | |
| --- | --- | --- | --- | --- | --- | --- | --- | --- | --- | --- | --- | --- | --- | --- | --- | --- |
|  | Basic info on area | Basic info on population | Accessibility in the region | Other relevant data | Population covered by EMS | EMS catchment area | EMS structure | Other EMS characteristics | Type of EMS dispatch system | Description of dispatch criteria | Structure of EMDC in the area | Other EMS response characteristics | Limitations in activation. E-g- weather etc. | Response time requirements | Fines for late response | Other response time characteristics |
| Abrams 2011^1^ | Y | Y | Y | Y | Y | Y | Y | Y | N | Y | Y | Y | N | N | N | N |
| Afzali 2013^2^ | Y | Y | Y | Y | Y | Y | Y | Y | N | Y | N | Y | Y | N | N | Y |
| Ahmoudi 2022^3^ | N | N | N | N | N | N | N | N | N | N | N | N | N | N | N | N |
| Al-Dury 2020^4^ | N | N | N | N | N | N | N | N | N | N | N | N | N | N | N | N |
| Alqudah 2021^5^ | Y | Y | Y | Y | Y | Y | Y | Y | Y | Y | Y | Y | N | N | N | N |
| Alumran 2020^6^ | Y | N | N | Y | N | N | Y | N | N | N | N | N | N | N | N | N |
| Aziz 2020^7^ | Y | Y | N | Y | Y | Y | Y | N | Y | Y | Y | Y | N | N | N | Y |
| Azpiazu 2024^8^ | Y | N | N | Y | Y | Y | Y | N | Y | Y | Y | Y | N | N | N | Y |
| Bagher 2017^9^ | Y | Y | Y | Y | Y | Y | Y | Y | Y | Y | Y | Y | N | N | N | Y |
| Baker 2008^10^ | Y | Y | Y | Y | Y | Y | Y | Y | Y | Y | Y | Y | N | N | N | Y |
| Berge 2005^11^ | Y | Y | Y | Y | Y | Y | Y | Y | N | Y | N | Y | Y | N | N | N |
| Biewener 2000^12^ | N | N | N | Y | N | Y | N | N | N | Y | N | N | N | N | N | N |
| Bjorkmann 2022^13^ | Y | N | N | Y | N | N | Y | Y | N | N | N | N | N | N | N | N |
| Bjornsson 2006^14^ | Y | Y | N | N | N | Y | Y | Y | N | Y | N | N | N | N | N | N |
| Blackwell 2009^15^ | Y | Y | Y | Y | Y | Y | Y | Y | Y | Y | N | Y | N | Y | N | N |
| Blaengsdottir 1994^16^ | Y | Y | N | Y | Y | Y | Y | Y | N | Y | N | N | N | N | N | N |
| Blanchard 2012^17^ | N | N | N | N | N | N | N | N | Y | Y | Y | Y | N | N | N | N |
| Bossers 2021^18^ | Y | N | N | Y | N | Y | Y | Y | N | Y | N | Y | N | N | N | N |
| Brede 2020^19^ | N | N | N | Y | N | N | N | N | N | N | N | N | N | N | N | N |
| Brison 1992^20^ | Y | Y | N | Y | Y | Y | Y | Y | N | N | N | Y | N | N | N | N |
| Brown 2019^21^ | Y | Y | Y | Y | Y | Y | Y | Y | N | Y | N | N | N | N | N | N |
| Bujak 2021^22^ | Y | Y | Y | Y | Y | Y | N | N | N | Y | N | N | N | N | N | N |
| Burger 2018^23^ | N | N | N | Y | N | N | N | N | N | N | N | N | N | N | N | N |
| Byrne 2019^24^ | N | Y | Y | Y | Y | N | N | Y | N | N | N | Y | N | N | N | Y |
| Cardoso 2014^25^ | N | N | Y | N | N | N | Y | Y | Y | Y | Y | Y | Y | Y | N | Y |
| Chang 2018^26^ | N | N | N | N | N | N | Y | Y | Y | Y | N | Y | N | N | N | N |
| Chen 2015^27^ | Y | N | Y | N | N | N | Y | Y | Y | N | Y | Y | N | Y | N | Y |
| Chesters 2015^28^ | Y | N | Y | N | Y | N | Y | N | N | Y | N | N | N | N | N | N |
| Claesson 2008^29^ | N | N | N | N | N | N | N | N | N | N | N | N | N | N | N | N |
| Deasy 2012^30^ | Y | Y | Y | Y | Y | Y | Y | Y | N | Y | Y | Y | N | N | N | N |
| deGraf 2019^31^ | Y | N | Y | Y | Y | N | Y | Y | Y | Y | Y | Y | N | N | N | N |
| Dicker 2018^32^ | Y | Y | Y | Y | Y | Y | Y | Y | Y | Y | Y | Y | N | N | N | N |
| Dinh 2023^33^ | Y | Y | Y | Y | Y | Y | Y | Y | Y | Y | Y | Y | N | N | N | N |
| Dyson 2013^34^ | Y | Y | Y | Y | Y | Y | Y | Y | N | N | N | N | N | N | N | N |
| Einarsson 1989^35^ | Y | Y | Y | Y | Y | Y | Y | Y | N | N | N | N | N | N | N | N |
| Eisenburger 2001^36^ | Y | Y | Y | Y | Y | Y | Y | N | N | N | N | N | N | N | N | N |
| Ong 20033 ^37^ | Y | Y | Y | Y | Y | Y | Y | N | N | N | N | N | N | N | N | N |
| Fake 2013^38^ | Y | Y | Y | Y | Y | Y | N | N | N | N | N | N | N | N | N | N |
| Finney 2003^39^ | Y | Y | Y | Y | Y | Y | Y | Y | N | N | N | N | N | N | N | N |
| Fothergill 2021^40^ | Y | Y | Y | Y | Y | Y | Y | Y | Y | Y | Y | Y | Y | N | N | Y |
| Fraga-Sastrias 2009^41^ | Y | Y | Y | Y | Y | Y | Y | Y | N | N | N | N | N | N | N | N |
| Gnesin 2021^42^ | Y | Y | Y | Y | Y | Y | Y | Y | Y | Y | Y | Y | N | N | N | N |
| Goh 2018^43^ | N | N | N | N | N | N | N | N | N | N | N | N | N | N | N | N |
| Goh 2013^44^ | N | N | N | N | N | N | N | N | N | N | N | N | N | N | N | N |
| Gregers 2021^45^ | N | N | N | N | N | N | N | N | N | N | N | N | N | N | N | N |
| Grmec 2009^46^ | Y | Y | Y | Y | Y | Y | Y | Y | N | N | N | N | N | N | N | N |
| Gunaga 2020^47^ | N | N | N | N | N | Y | Y | Y | N | N | N | N | N | N | N | N |
| Han 2022^48^ | Y | N | N | N | N | N | N | N | N | N | N | N | N | N | N | N |
| Hayes 2010^49^ | N | N | N | N | N | N | N | N | N | N | N | N | N | N | N | N |
| Henry 2013^50^ | Y | Y | Y | Y | Y | Y | Y | Y | Y | Y | Y | Y | N | N | N | N |
| Herlitz 2006^51^ | N | N | N | N | Y | N | N | N | N | N | N | N | N | N | N | N |
| Herlitz 2008^52^ | N | N | N | N | Y | N | N | N | N | N | N | N | N | N | N | N |
| Hillis 1993^53^ | Y | Y | Y | N | Y | Y | Y | Y | N | N | N | Y | N | N | N | N |
| Holmen 2020^54^ | Y | Y | Y | Y | Y | N | N | N | N | N | N | N | N | N | N | N |
| Huabbangyang 2021^55^ | N | N | N | N | N | N | N | N | N | N | N | N | N | N | N | N |
| Hubert 2016^56^ | N | N | N | N | N | N | Y | N | N | N | N | N | N | N | N | N |
| Jennings 2006^57^ | Y | Y | Y | Y | Y | Y | Y | Y | Y | Y | Y | Y | N | N | N | N |
| Jeong 2017^58^ | N | N | N | N | N | N | N | N | N | N | N | N | N | N | N | N |
| Kennedy 2023^59^ | Y | Y | Y | N | Y | Y | Y | Y | Y | Y | N | N | N | N | N | N |
| Kentsch 2000^60^ | Y | Y | Y | Y | Y | Y | Y | Y | N | N | N | N | Y | N | N | N |
| Kitano 2022^61^ | N | N | N | N | N | N | Y | Y | Y | Y | N | Y | N | N | N | N |
| Klosiewiecz 2017^62^ | Y | Y | Y | Y | Y | Y | Y | Y | Y | Y | Y | Y | N | N | N | N |
| Lee 2019^63^ | N | N | N | N | N | N | Y | Y | Y | Y | N | N | N | N | N | N |
| Lee 2013^64^ | Y | Y | Y | Y | Y | Y | Y | Y | N | N | N | N | N | N | N | N |
| Leung 2001^65^ | Y | Y | Y | Y | Y | Y | Y | Y | N | N | N | N | N | N | N | N |
| Lim 2020^66^ | Y | Y | Y | Y | Y | Y | Y | Y | Y | Y | Y | Y | N | N | N | N |
| Lin 2014^67^ | N | N | N | N | Y | N | N | N | N | N | N | N | N | N | N | N |
| Little 2020^68^ | Y | Y | Y | Y | Y | Y | N | N | N | N | N | N | N | N | N | N |
| Liu 2023^69^ | Y | Y | Y | Y | Y | Y | Y | Y | Y | Y | Y | Y | N | N | N | N |
| Lyon 2013^70^ | Y | Y | Y | Y | Y | Y | Y | Y | N | N | N | N | N | N | N | N |
| Margey 2011^71^ | Y | Y | Y | Y | Y | Y | Y | Y | Y | Y | Y | Y | Y | Y | N | N |
| Mathiesen 2018^72^ | Y | Y | Y | Y | Y | Y | Y | Y | Y | Y | Y | Y | N | N | N | N |
| Mayer 1979^73^ | Y | Y | Y | N | N | N | Y | Y | N | N | N | N | N | N | N | N |
| Meyer 2001^74^ | Y | Y | Y | Y | Y | Y | Y | Y | Y | Y | Y | N | N | N | N | N |
| Mikiewicz 2023^75^ | Y | Y | Y | Y | Y | Y | Y | Y | Y | Y | N | N | N | N | N | N |
| Mikkelsen 2017^76^ | Y | Y | Y | Y | Y | Y | Y | Y | Y | Y | N | N | N | N | N | N |
| Mills 2023^77^ | Y | N | N | N | N | N | Y | Y | Y | Y | N | N | N | N | N | N |
| Mills 2019^78^ | Y | Y | Y | Y | Y | Y | Y | Y | Y | Y | Y | Y | N | N | N | N |
| Mogensen 2015^79^ | Y | Y | Y | Y | Y | Y | Y | Y | N | N | N | N | N | N | N | N |
| Morrisey 1996^80^ | Y | Y | Y | Y | Y | Y | Y | N | N | N | N | N | N | N | N | N |
| Nadolny 2021^81^ | Y | Y | Y | Y | Y | Y | Y | Y | N | N | N | N | N | N | N | N |
| Naroo 2012^82^ | N | N | N | N | N | N | N | N | N | N | N | N | N | N | N | N |
| Navab 2019^83^ | N | N | N | N | N | N | Y | Y | N | N | N | N | N | N | N | N |
| Nichol 2016^84^ | Y | Y | Y | N | N | N | Y | Y | N | N | N | N | N | N | N | N |
| Nielsen 2022^85^ | Y | Y | Y | Y | Y | Y | Y | Y | N | Y | N | N | Y | N | N | N |
| O’Keefe 2011^86^ | Y | N | Y | Y | N | N | Y | Y | Y | Y | Y | Y | N | N | N | N |
| Park 2017^87^ | Y | Y | Y | Y | Y | N | N | N | N | N | N | N | N | N | N | N |
| Pell 2001^88^ | Y | Y | Y | Y | Y | Y | Y | Y | N | N | N | N | N | N | N | N |
| Pons 2005^89^ | Y | Y | Y | Y | Y | Y | N | N | N | N | N | N | N | N | N | N |
| Pons 2002^90^ | N | N | Y | Y | N | N | N | Y | N | N | N | N | N | N | N | N |
| Puolakka 2023^91^ | Y | Y | Y | Y | Y | Y | Y | Y | Y | Y | Y | Y | N | N | N | N |
| Do 2010^92^ | Y | Y | Y | Y | Y | Y | Y | Y | N | N | N | N | N | N | N | N |
| Rajan 2016^93^ | N | N | N | N | N | N | N | N | N | N | N | N | N | N | N | N |
| Renkiewicz 2014^94^ | Y | Y | Y | Y | Y | Y | Y | Y | N | N | N | N | N | N | N | N |
| Schinnerl 1990^95^ | N | N | N | N | N | N | Y | Y | Y | Y | N | N | N | N | N | N |
| Semensato 2011^96^ | Y | Y | Y | Y | Y | Y | Y | Y | N | N | N | N | N | N | N | N |
| Shah 2010^97^ | Y | Y | Y | Y | Y | Y | Y | Y | N | N | N | N | N | N | N | N |
| Shepherd 2008^98^ | Y | Y | Y | Y | Y | Y | N | N | N | N | N | N | Y | N | N | N |
| Sigursson 2000^99^ | Y | Y | Y | Y | Y | Y | Y | Y | N | N | N | N | N | N | N | N |
| Sipria 2016^100^ | Y | Y | Y | Y | N | N | N | N | N | N | N | N | N | N | N | N |
| Spaite 2008^101^ | Y | Y | Y | Y | Y | Y | Y | Y | N | N | N | N | N | N | N | N |
| Stoeckl 1020^102^ | N | N | N | N | N | N | N | N | N | N | N | N | N | N | N | N |
| Stromsoe 2015^103^ | Y | Y | Y | Y | Y | Y | Y | Y | N | N | N | N | N | N | N | N |
| Stromsoe 2011^104^ | Y | Y | Y | Y | Y | Y | Y | Y | N | N | N | N | N | N | N | N |
| Sugiyama 2023^105^ | Y | Y | Y | Y | Y | Y | Y | Y | N | N | N | N | N | N | N | N |
| Syvaoja 2018^106^ | Y | N | Y | N | N | N | Y | Y | Y | Y | N | N | N | N | N | N |
| TerAvest 2019^107^ | Y | Y | Y | N | N | Y | Y | Y | N | N | N | N | N | N | N | N |
| Thompson 2017^108^ | Y | Y | Y | Y | Y | Y | Y | Y | Y | Y | N | N | Y | N | N | N |
| Trenkler 2012^109^ | Y | Y | Y | Y | Y | Y | Y | Y | N | N | N | N | N | N | N | N |
| Tsai 2017^110^ | Y | Y | Y | Y | Y | Y | Y | Y | N | N | N | N | N | N | N | N |
| Wai 2005^111^ | N | N | N | N | Y | Y | Y | N | N | N | N | N | N | N | N | N |
| Weinlich 2019^112^ | N | N | N | N | N | N | Y | Y | N | N | Y | N | N | N | N | N |
| Wik 2003^113^ | Y | Y | Y | Y | Y | Y | Y | Y | N | N | N | N | N | N | N | N |
| Wissa 2021^114^ | Y | Y | Y | Y | Y | Y | Y | Y | N | N | N | N | N | N | N | N |
| Zheng 2023^115^ | Y | Y | Y | Y | Y | Y | Y | Y | N | N | N | N | N | N | N | N |

EMS: Emergency medical services; Y: yes; N: no.

**Supplemental table 2. Data extraction (continued).**

|  | RESPONSE TIMES CHARACTERISTICS | | | | | | | | | | | |
| --- | --- | --- | --- | --- | --- | --- | --- | --- | --- | --- | --- | --- |
|  | How was response time data defined? | How was response time data measured? | How was response time data stored? | How was response time data information used? | What were the primary and secondary outcomes? | Was response time correlated to mortality? | Was response time correlated to outcome and AE? | Was response time correlated to diagnoses? | Was response time correlated to distance? | Was response time correlated to receiving hospital | Was response time correlated to capacity? | Other response time data described? |
| Abrams 2011^1^ | Y | Y | Y | Y | Y | Y | Y | Y | N | N | N | Y |
| Afzali 2013^2^ | Y | Y | N | Y | Y | N | N | N | N | N | N | Y |
| Ahmoudi 2022^3^ | Y | Y | N | Y | Y | Y | Y | Y | N | N | N | N |
| Al-Dury 2020^4^ | Y | Y | N | Y | Y | Y | Y | Y | N | N | N | Y |
| Alqudah 2021^5^ | Y | Y | N | Y | Y | Y | Y | Y | N | N | N | Y |
| Alumran 2020^6^ | Y | Y | N | Y | Y | Y | Y | Y | N | N | N | N |
| Aziz 2020^7^ | Y | Y | N | Y | Y | Y | Y | Y | N | N | N | N |
| Bagher 2017^8^ | Y | Y | N | Y | Y | Y | Y | Y | N | N | N | Y |
| Azpiazu 2024^9^ | Y | Y | N | Y | Y | Y | Y | Y | N | N | N | Y |
| Baker 2008^10^ | Y | Y | N | Y | Y | Y | Y | Y | N | N | N | Y |
| Berge 2005^11^ | Y | Y | N | Y | Y | N | N | N | N | N | N | N |
| Biewener 2000^12^ | N | N | N | N | Y | N | N | N | N | N | N | N |
| Bjorkmann 2022^13^ | Y | Y | Y | Y | Y | Y | Y | Y | Y | N | N | N |
| Bjornsson 2006^14^ | Y | Y | N | Y | Y | N | N | N | N | N | N | N |
| Blackwell 2009^15^ | Y | Y | Y | Y | Y | Y | Y | Y | N | N | N | N |
| Blaengsdottir 1994^16^ | Y | Y | N | Y | Y | N | N | N | N | N | N | N |
| Blanchard 2012^17^ | Y | Y | N | Y | Y | Y | Y | Y | N | N | N | N |
| Bossers 2021^18^ | Y | Y | N | Y | Y | N | N | N | N | N | N | N |
| Brede 2020^19^ | Y | Y | Y | Y | Y | N | N | N | N | N | N | N |
| Brison 1992^20^ | Y | Y | Y | Y | Y | Y | Y | N | N | N | N | N |
| Brown 2019^21^ | Y | Y | Y | Y | Y | Y | Y | Y | N | N | N | N |
| Bujak 2021^22^ | Y | Y | Y | Y | Y | Y | Y | Y | N | N | N | N |
| Burger 2018^23^ | Y | Y | Y | Y | Y | Y | Y | Y | N | N | N | N |
| Byrne 2019^24^ | Y | Y | Y | Y | Y | Y | Y | Y | Y | N | N | Y |
| Cardoso 2014^25^ | Y | Y | Y | Y | Y | N | N | N | N | N | N | N |
| Chang 2018^26^ | Y | Y | Y | Y | Y | Y | Y | Y | N | N | N | Y |
| Chen 2015^27^ | Y | Y | Y | Y | Y | Y | Y | Y | N | N | N | N |
| Chesters 2015^28^ | Y | Y | Y | Y | Y | N | N | N | N | N | N | N |
| Claesson 2008^29^ | Y | Y | Y | Y | Y | Y | Y | Y | Y | N | N | N |
| Deasy 2012^30^ | Y | Y | Y | Y | Y | Y | Y | Y | N | N | N | N |
| deGraf 2019^31^ | Y | Y | Y | Y | Y | Y | Y | Y | N | N | N | N |
| Dicker 2018^32^ | Y | Y | Y | Y | Y | Y | N | N | N | N | N | N |
| Dinh 2023^33^ | Y | Y | Y | Y | Y | Y | N | N | N | N | N | N |
| Dyson 2013^34^ | Y | Y | Y | Y | Y | Y | Y | Y | N | N | N | N |
| Einarsson 1989^35^ | Y | Y | Y | Y | Y | Y | Y | N | N | N | N | N |
| Eisenburger 2001^36^ | Y | Y | Y | Y | Y | Y | Y | Y | N | N | N | N |
| Ong 2003^37^ | Y | Y | Y | Y | Y | Y | Y | Y | N | N | N | N |
| Fake 2013^38^ | Y | Y | Y | Y | Y | Y | Y | Y | N | N | N | N |
| Finney 2003^39^ | Y | Y | Y | Y | Y | Y | N | N | N | N | N | N |
| Fothergill 2021^40^ | Y | Y | Y | Y | Y | Y | Y | Y | N | N | N | N |
| Fraga-Sastrias 2009^41^ | Y | Y | Y | Y | Y | Y | Y | N | N | N | N | N |
| Gnesin 2021^42^ | Y | Y | Y | Y | Y | Y | Y | Y | N | N | N | N |
| Goh 2018^43^ | Y | Y | Y | Y | Y | Y | Y | N | N | N | N | N |
| Goh 2013^44^ | Y | Y | Y | Y | Y | N | N | N | N | N | N | N |
| Gregers 2021^45^ | N | N | N | N | Y | N | N | N | N | N | N | N |
| Grmec 2009^46^ | Y | Y | Y | Y | Y | Y | Y | N | N | N | N | N |
| Gunaga 2020^47^ | Y | Y | Y | Y | Y | Y | N | N | N | N | N | N |
| Han 2022^48^ | Y | Y | Y | Y | Y | Y | N | N | N | N | N | N |
| Hayes 2010^49^ | N | N | N | Y | Y | N | N | N | N | N | N | N |
| Henry 2013^50^ | Y | Y | Y | Y | Y | Y | Y | N | N | N | N | N |
| Herlitz 2006^51^ | Y | Y | Y | Y | Y | Y | N | N | N | N | N | N |
| Herlitz 2008^52^ | Y | Y | Y | Y | Y | Y | N | N | N | N | N | N |
| Hillis 1993^53^ | Y | N | N | N | Y | Y | Y | N | N | N | N | N |
| Holmen 2020^54^ | Y | Y | Y | Y | Y | Y | Y | N | N | N | N | N |
| Huabbangyang 2021^55^ | Y | Y | Y | Y | Y | Y | Y | N | N | N | N | N |
| Hubert 2016^56^ | Y | Y | Y | Y | Y | Y | Y | N | N | N | N | N |
| Jennings 2006^57^ | Y | Y | Y | Y | Y | Y | Y | N | N | N | N | N |
| Jeong 2017^58^ | N | N | N | N | Y | Y | Y | Y | N | N | N | N |
| Kennedy 2023^59^ | N | Y | Y | Y | Y | Y | N | N | N | N | N | N |
| Kentsch 2000^60^ | N | N | N | N | Y | Y | N | N | N | N | N | N |
| Kitano 2022^61^ | Y | Y | Y | Y | Y | Y | Y | N | N | N | N | N |
| Klosiewiecz 2017^62^ | Y | Y | Y | Y | Y | Y | Y | N | N | N | N | N |
| Lee 2019^63^ | Y | Y | Y | Y | Y | Y | Y | Y | N | N | N | N |
| Lee 2013^64^ | Y | Y | Y | Y | Y | N | N | N | N | N | N | N |
| Leung 2001^65^ | Y | Y | Y | Y | Y | Y | N | N | N | N | N | N |
| Lim 2020^66^ | Y | Y | Y | Y | Y | Y | N | N | N | N | N | N |
| Lin 2014^67^ | N | N | N | N | Y | Y | Y | Y | N | N | N | N |
| Little 2020^68^ | N | N | N | N | Y | Y | Y | Y | N | N | N | N |
| Liu 2023^69^ | Y | Y | Y | Y | Y | Y | Y | Y | N | N | N | N |
| Lyon 2013^70^ | Y | Y | Y | Y | Y | Y | Y | N | N | N | N | N |
| Margey 2011^71^ | Y | Y | Y | Y | Y | Y | Y | N | N | N | N | N |
| Mathiesen 2018^72^ | Y | Y | Y | Y | Y | Y | Y | Y | N | N | N | N |
| Mayer 1979^73^ | N | N | N | N | Y | Y | N | N | N | N | N | N |
| Meyer 2001^74^ | Y | Y | Y | Y | Y | Y | Y | Y | N | N | N | N |
| Mikiewicz 2023^75^ | Y | Y | Y | Y | Y | Y | Y | Y | N | N | N | N |
| Mikkelsen 2017^76^ | Y | Y | Y | Y | Y | Y | Y | Y | N | N | N | N |
| Mills 2023^77^ | Y | Y | Y | Y | Y | Y | Y | Y | N | N | N | N |
| Mills 2019^78^ | Y | Y | Y | Y | Y | Y | Y | Y | N | N | N | N |
| Mogensen 2015^79^ | Y | Y | Y | Y | Y | Y | Y | N | N | N | N | N |
| Morrisey 1996^80^ | Y | Y | Y | Y | Y | Y | Y | N | N | N | N | N |
| Nadolny 2021^81^ | Y | Y | Y | Y | Y | Y | Y | N | N | N | N | N |
| Naroo 2012^82^ | N | N | N | N | Y | Y | N | N | N | N | N | N |
| Navab 2019^83^ | Y | Y | Y | Y | Y | Y | N | N | N | N | N | N |
| Nichol 2016^84^ | Y | Y | Y | Y | Y | Y | N | N | N | N | N | N |
| Nielsen 2022^85^ | Y | Y | Y | Y | Y | Y | Y | Y | N | N | N | N |
| O’Keefe 2011^86^ | Y | Y | Y | Y | Y | Y | Y | Y | N | N | N | N |
| Park 2017^87^ | Y | Y | Y | Y | Y | Y | Y | Y | N | N | N | N |
| Pell 2001^88^ | Y | Y | Y | Y | Y | Y | N | N | N | N | N | N |
| Pons 2005^89^ | Y | Y | Y | Y | Y | Y | N | N | N | N | N | N |
| Pons 2002^90^ | Y | Y | Y | Y | Y | Y | N | N | N | N | N | N |
| Puolakka 2023^91^ | Y | Y | Y | Y | Y | Y | Y | Y | N | N | N | N |
| Do 2010^92^ | Y | Y | Y | Y | N | N | N | N | N | N | N | N |
| Rajan 2016^93^ | Y | Y | Y | Y | Y | Y | Y | Y | N | N | N | N |
| Renkiewicz 2014^94^ | Y | Y | Y | Y | Y | Y | N | N | N | N | N | N |
| Schinnerl 1990^95^ | Y | Y | Y | Y | Y | Y | N | N | N | N | N | N |
| Semensato 2011^96^ | Y | Y | Y | Y | Y | Y | N | N | N | N | N | N |
| Shah 2010^97^ | Y | Y | Y | Y | Y | Y | Y | N | N | N | N | N |
| Shepherd 2008^98^ | Y | Y | Y | Y | Y | N | N | N | N | N | N | N |
| Sigursson 2000^99^ | Y | Y | Y | Y | Y | Y | N | N | N | N | N | N |
| Sipria 2016^100^ | Y | Y | Y | Y | Y | N | N | N | N | N | N | N |
| Spaite 2008^101^ | Y | Y | Y | Y | Y | Y | N | N | N | N | N | N |
| Stoeckl 2010^102^ | Y | Y | Y | Y | Y | Y | N | N | N | N | N | N |
| Stromsoe 2015^103^ | Y | Y | Y | Y | Y | Y | N | N | N | N | N | N |
| Stromsoe 2011^104^ | Y | Y | Y | Y | Y | Y | N | N | N | N | N | N |
| Sugiyama 2023^105^ | Y | Y | Y | Y | Y | Y | Y | N | N | N | N | N |
| Syvaoja 2018^106^ | Y | Y | Y | Y | Y | Y | N | N | N | N | N | N |
| TerAvest 2019^107^ | Y | Y | Y | Y | Y | Y | N | N | N | N | N | N |
| Thompson 2017^108^ | Y | Y | Y | Y | Y | Y | N | N | N | N | N | N |
| Trenkler 2012^109^ | Y | Y | Y | Y | Y | Y | N | N | N | N | N | N |
| Tsai 2017^110^ | Y | Y | Y | Y | Y | Y | N | N | N | N | N | N |
| Wai 2005^111^ | Y | Y | Y | Y | Y | Y | N | N | N | N | N | N |
| Weinlich 2019^112^ | Y | Y | Y | Y | Y | Y | Y | Y | Y | N | N | N |
| Wik 2003^113^ | Y | Y | Y | Y | Y | Y | N | N | N | N | N | N |
| Wissa 2021^114^ | Y | Y | Y | Y | Y | Y | Y | N | N | N | N | N |
| Zheng 2023^115^ | Y | Y | Y | Y | Y | Y | Y | N | N | N | N | N |

Y: yes; N: no.

**Supplemental table 3. Quality appraisal**

|  | INTERNAL VALIDITY | | | | | EXTERNAL VALIDITY | | | | | | |
| --- | --- | --- | --- | --- | --- | --- | --- | --- | --- | --- | --- | --- |
|  | Is the author employed in the EMS organization? | Does the literature provide reference to where data were obtained? | Does the literature provide reference to how data were obtained? | Do the authors declare conflicts of interest? | Has an ethics committee approved the reporting? | Is the EMS structure clearly described? | Is the type of EMS dispatch system clearly described? | Are the response time characteristics clearly described? | Are missing data accounted for? | Are other limitations discussed? | Is the study design clearly described? | Are the primary and secondary outcomes clearly described? |
| Abrams 2011^1^ | Y | Y | Y | Y | Y | Y | Y | Y | Y | Y | Y | Y |
| Afzali 2013^2^ | Y | Y | Y | Y | Y | Y | Y | Y | Y | Y | Y | Y |
| Ahmoudi 2022^3^ | Y | Y | Y | N | N | N | N | Y | N | Y | Y | Y |
| Al-Dury 2020^4^ | N | Y | Y | Y | Y | N | N | Y | Y | Y | Y | Y |
| Alqudah 2021^5^ | Y | Y | Y | Y | Y | Y | N | Y | Y | Y | Y | Y |
| Alumran 2020^6^ | Y | Y | Y | Y | Y | N | N | Y | Y | Y | Y | Y |
| Aziz 2020^7^ | Y | Y | Y | Y | Y | Y | N | Y | N | Y | Y | Y |
| Axpiazu 2024^8^ | Y | Y | Y | Y | Y | Y | N | Y | N | Y | Y | Y |
| Bagher 2017^9^ | Y | Y | Y | Y | Y | Y | Y | Y | Y | Y | Y | Y |
| Baker 2008^10^ | Y | Y | Y | Y | Y | Y | Y | Y | Y | Y | Y | Y |
| Berge 2005^11^ | N | Y | Y | N | N | Y | N | N | Y | Y | Y | Y |
| Biewener 2000^12^ | N | Y | Y | Y | N | N | N | N | N | N | Y | Y |
| Bjorkmann 2022^13^ | Y | Y | Y | Y | Y | N | N | Y | Y | Y | Y | Y |
| Bjornsson 2006^14^ | Y | Y | Y | N | N | N | N | N | N | N | Y | Y |
| Blackwell 2009^15^ | Y | Y | Y | N | Y | Y | N | Y | N | Y | Y | Y |
| Blaengsdottir 1994^16^ | Y | Y | Y | N | Y | Y | N | N | N | Y | Y | Y |
| Blanchard 2012^17^ | Y | Y | Y | N | Y | N | Y | Y | Y | Y | Y | Y |
| Bossers 2021^18^ | Y | Y | Y | Y | Y | N | N | N | N | Y | Y | Y |
| Brede 2020^19^ | Y | Y | Y | Y | Y | N | N | N | N | Y | Y | Y |
| Brison 1992^20^ | N | Y | Y | N | N | Y | N | N | N | N | Y | Y |
| Brown 2019^21^ | N | Y | Y | N | N | Y | Y | Y | Y | Y | Y | Y |
| Bujak 2021^22^ | N | Y | Y | Y | Y | Y | N | Y | Y | Y | Y | Y |
| Burger 2018^23^ | N | Y | Y | Y | Y | N | N | Y | Y | Y | Y | Y |
| Byrne 2019^24^ | N | Y | Y | Y | Y | N | N | Y | Y | Y | Y | Y |
| Cardoso 2014^25^ | Y | Y | Y | Y | Y | N | Y | Y | Y | N | Y | Y |
| Chang 2018^26^ | Y | Y | Y | Y | Y | Y | N | Y | Y | Y | Y | Y |
| Chen 2015^27^ | Y | Y | Y | Y | Y | Y | Y | Y | N | Y | Y | Y |
| Chesters 2015^28^ | Y | Y | Y | N | N | Y | N | N | Y | Y | Y | Y |
| Claesson 2008^29^ | N | Y | Y | Y | N | N | N | Y | N | Y | Y | Y |
| Deasy 2012^30^ | Y | Y | Y | Y | Y | Y | Y | Y | Y | Y | Y | Y |
| deGraf 2019^31^ | N | Y | Y | Y | Y | Y | Y | Y | Y | Y | Y | Y |
| Dicker 2018^32^ | Y | Y | Y | Y | Y | Y | Y | Y | N | Y | Y | Y |
| Dinh 2023^33^ | Y | Y | Y | Y | Y | Y | Y | Y | N | Y | Y | Y |
| Dyson 2013^34^ | Y | Y | Y | Y | Y | Y | Y | Y | Y | Y | Y | Y |
| Einarsson 1989^35^ | Y | Y | Y | N | N | Y | Y | Y | N | N | Y | Y |
| Eisenburger 2001^36^ | Y | Y | Y | N | N | Y | Y | Y | Y | N | Y | Y |
| Ong 2003^37^ | Y | Y | Y | N | N | Y | N | Y | Y | N | Y | Y |
| Fake 2013^38^ | N | Y | Y | Y | Y | Y | Y | Y | Y | Y | N | N |
| Finney 2003^39^ | Y | Y | Y | Y | Y | Y | Y | Y | Y | Y | Y | Y |
| Fothergill 2021^40^ | Y | Y | Y | Y | Y | Y | Y | N | N | Y | Y | Y |
| Fraga-Sastrias 2009^41^ | N | Y | Y | N | Y | Y | Y | Y | Y | Y | Y | Y |
| Gnesin 2021^42^ | N | Y | Y | Y | Y | Y | Y | Y | Y | Y | Y | Y |
| Goh 2018^43^ | N | Y | Y | Y | Y | N | N | Y | N | Y | Y | Y |
| Goh 2013^44^ | N | Y | Y | N | N | N | N | Y | N | Y | Y | Y |
| Gregers 2021^45^ | Y | Y | Y | N | N | N | N | N | N | N | Y | Y |
| Grmec 2009^46^ | Y | Y | Y | Y | N | Y | Y | Y | Y | Y | Y | Y |
| Gunaga 2020^47^ | N | Y | Y | N | N | N | N | Y | N | N | Y | Y |
| Han 2022^48^ | N | Y | Y | Y | N | N | Y | Y | Y | N | N | Y |
| Hayes 2010^49^ | N | Y | Y | N | N | N | N | Y | N | N | N | Y |
| Henry 2013^50^ | Y | Y | Y | Y | Y | Y | Y | Y | N | Y | Y | Y |
| Herlitz 2006^51^ | N | Y | Y | Y | N | N | N | Y | N | Y | Y | Y |
| Herlitz 2008^52^ | N | Y | Y | Y | N | N | N | Y | N | Y | Y | Y |
| Hillis 1993^53^ | N | Y | Y | N | N | Y | Y | Y | N | Y | Y | Y |
| Holmen 2020^54^ | N | Y | Y | Y | N | Y | N | Y | N | Y | Y | Y |
| Huabbangyang 2021^55^ | N | Y | Y | Y | N | N | N | Y | Y | Y | Y | Y |
| Hubert 2016^56^ | N | Y | Y | N | Y | Y | N | Y | Y | Y | Y | Y |
| Jennings 2006^57^ | Y | Y | Y | Y | Y | Y | Y | Y | Y | Y | Y | Y |
| Jeong 2017^58^ | N | Y | Y | N | N | N | N | N | N | N | Y | Y |
| Kennedy 2023^59^ | Y | Y | Y | Y | Y | Y | Y | Y | Y | Y | Y | Y |
| Kentsch 2000^60^ | Y | Y | Y | N | N | Y | Y | Y | N | N | Y | Y |
| Kitano 2022^61^ | N | Y | Y | Y | Y | Y | Y | Y | Y | Y | Y | Y |
| Klosiewiecz 2017^62^ | N | Y | Y | Y | N | Y | Y | Y | Y | Y | Y | Y |
| Lee 2019^63^ | N | Y | Y | Y | Y | Y | Y | Y | Y | Y | Y | Y |
| Lee 2013^64^ | N | Y | Y | N | Y | Y | Y | Y | N | Y | Y | Y |
| Leung 2001^65^ | N | Y | Y | N | N | Y | Y | Y | N | Y | Y | Y |
| Lim 2020^66^ | N | Y | Y | Y | Y | Y | Y | Y | N | Y | Y | Y |
| Lin 2014^67^ | N | Y | Y | Y | N | N | N | Y | N | N | N | N |
| Little 2020^68^ | N | Y | Y | Y | Y | N | N | Y | N | Y | Y | Y |
| Liu 2023^69^ | N | Y | Y | Y | N | Y | Y | Y | N | N | Y | Y |
| Lyon 2013^70^ | Y | Y | Y | Y | Y | N | N | Y | N | Y | Y | Y |
| Margey 2011^71^ | N | Y | Y | Y | N | Y | Y | Y | N | Y | Y | Y |
| Mathiesen 2018^72^ | N | Y | Y | Y | Y | Y | Y | Y | Y | Y | Y | Y |
| Mayer 1979^73^ | N | Y | Y | N | N | Y | Y | N | N | N | Y | Y |
| Meyer 2001^74^ | N | Y | Y | N | Y | Y | Y | Y | Y | Y | Y | Y |
| Mikiewicz 2023^75^ | N | Y | Y | N | Y | Y | Y | Y | Y | Y | Y | Y |
| Mikkelsen 2017^76^ | Y | Y | Y | Y | Y | Y | Y | Y | Y | Y | Y | Y |
| Mills 2023^77^ | N | Y | Y | Y | Y | Y | Y | Y | N | Y | Y | Y |
| Mills 2019^78^ | N | Y | Y | Y | Y | Y | Y | Y | N | Y | Y | Y |
| Mogensen 2015^79^ | Y | Y | Y | N | Y | Y | Y | Y | N | Y | Y | Y |
| Morrisey 1996^80^ | N | Y | Y | N | N | Y | Y | Y | N | N | Y | Y |
| Nadolny 2021^81^ | Y | Y | Y | Y | Y | Y | Y | Y | N | Y | Y | Y |
| Naroo 2012^82^ | N | Y | Y | N | N | N | N | N | N | N | N | Y |
| Navab 2019^83^ | N | Y | Y | Y | Y | N | N | Y | Y | Y | Y | Y |
| Nichol 2016^84^ | Y | Y | Y | Y | Y | Y | N | Y | N | Y | Y | Y |
| Nielsen 2022^85^ | N | Y | Y | Y | Y | Y | Y | Y | Y | Y | Y | Y |
| O’Keefe 2011^86^ | N | Y | Y | Y | Y | Y | N | Y | Y | Y | Y | Y |
| Park 2017^87^ | N | Y | Y | N | Y | Y | Y | Y | Y | Y | Y | Y |
| Pell 2001^88^ | N | Y | Y | Y | N | Y | Y | Y | Y | N | Y | Y |
| Pons 2005^89^ | N | Y | Y | N | N | Y | Y | Y | N | N | Y | Y |
| Pons 2002^90^ | Y | Y | Y | N | N | N | N | Y | N | Y | Y | Y |
| Puolakka 2023^91^ | Y | Y | Y | Y | Y | Y | Y | Y | Y | Y | Y | Y |
| Do 2010^92^ | Y | Y | Y | Y | N | Y | Y | Y | N | Y | Y | Y |
| Rajan 2016^93^ | N | Y | Y | Y | Y | N | N | Y | Y | Y | Y | Y |
| Renkiewicz 2014^94^ | Y | Y | Y | Y | Y | Y | Y | Y | Y | Y | Y | Y |
| Schinnerl 1990^95^ | N | Y | Y | N | N | N | N | Y | N | N | Y | Y |
| Semensato 2011^96^ | N | Y | Y | Y | Y | Y | Y | Y | N | Y | Y | Y |
| Shah 2010^97^ | N | Y | Y | Y | Y | Y | Y | Y | N | Y | Y | Y |
| Shepherd 2008^98^ | N | Y | Y | Y | Y | Y | Y | Y | Y | Y | Y | Y |
| Sigursson 2000^99^ | Y | Y | Y | N | N | Y | Y | Y | N | N | Y | Y |
| Sipria 2016^100^ | Y | Y | Y | Y | N | Y | Y | Y | N | N | Y | Y |
| Spaite 2008^101^ | N | Y | Y | Y | N | Y | Y | Y | Y | Y | Y | Y |
| Stoeckl 2010^102^ | Y | N | N | N | N | N | N | N | N | N | Y | Y |
| Stromsoe 2015^103^ | N | Y | Y | Y | Y | Y | Y | Y | N | Y | Y | Y |
| Stromsoe 2011^104^ | N | Y | Y | Y | Y | Y | Y | Y | Y | Y | Y | Y |
| Sugiyama 2023^105^ | N | Y | Y | Y | Y | Y | Y | Y | N | Y | Y | Y |
| Syvaoja 2018^106^ | N | Y | Y | Y | Y | Y | Y | Y | N | Y | Y | Y |
| TerAvest 2019^107^ | Y | Y | Y | Y | Y | N | N | Y | N | Y | Y | Y |
| Thompson 2017^108^ | Y | Y | Y | Y | Y | Y | Y | Y | N | Y | Y | Y |
| Trenkler 2012^109^ | N | Y | Y | N | N | Y | N | N | N | N | Y | Y |
| Tsai 2017^110^ | N | Y | Y | Y | Y | Y | Y | Y | N | Y | Y | Y |
| Wai 2005^111^ | Y | Y | Y | N | N | N | N | Y | N | N | Y | Y |
| Weinlich 2019^112^ | N | Y | Y | Y | N | N | N | Y | N | Y | Y | Y |
| Wik 2003^113^ | N | Y | Y | N | Y | Y | Y | Y | N | N | Y | Y |
| Wissa 2021^114^ | Y | Y | Y | Y | Y | Y | Y | Y | N | Y | Y | Y |
| Zheng 2023^115^ | Y | Y | Y | Y | N | N | N | Y | N | Y | Y | Y |

EMS: Emergency medical service; Y: yes; N: no.

**Supplemental Table 4: Reported outcome measures categorized**

**Category** **Reported specific outcome measures within each category**

**Patient-centered** Survival to discharge; 30-days survival; return of spontaneous circulation; **outcomes** cerebral performance category; 24H-survival; favorable neurological outcome;

Neurological outcome; intensive care unit admission; Glasgow outcome scale, length of stay, 90-days survival; 1-year survival; 7-days survival; death;

extended Glasgow outcome scale; functional outcome, Sickness Impact Profile;

**EMS-centered** Response time; on-scene time; call-to-arrival time; presenting rhythm, total

**measures** prehospital time, collapse-to-call time; total transport time; collapse-to-hospital time; population density; collapse-to-defibrillation time; CPR duration;

3 minutes CPR first vs. defibrillation first; defibrillation time; time to dispatch; arrest-to-BLS time; physician presence; resuscitation time, dispatch interval

Legend: EMS: emergency medical services, 24H: 24 hours; CPR: cardiopulmonary resuscitation; BLS: basic life support

| Supplemental material Table 5: Summary of results for each outcome category | | | |
| --- | --- | --- | --- |
|  | **Specific outcome measures** | **Total number of studies** | **Summary of results** |
| Patient- centered outcomes | Survival to discharge | n = 68 | Reported in the majority of the included studies |
|  | 30-days survival | n = 31 | Reported in 31 studies |
|  | Return of spontaneous circulation | n = 23 | Reported in twenty-three studies |
|  | Cerebral Performance Category | n = 13 | Reported in thirteen studies |
|  | 24H-survival | n = 6 | Reported in six studies |
|  | Favorable neurological outcome | n = 4 | Reported in four studies |
|  | Intensive care unit admission | n = 3 | Reported in three studies |
|  | Glasgow outcome scale | n = 2 | Reported in two studies |
|  | Length of Hospital stay | n = 2 | Reported in two studies |
|  | 90-days survival | n = 2 | Reported in two studies |
|  | 1-year survival | n = 1 | Reported in one study |
|  | 7-days survival | n = 1 | Reported in one study |
|  | Death | n = 1 | Unspecified. Reported in one study |
|  | Extended Glasgow Outcome Scale | n = 1 | Neurological outcome assessment reported in one study |
|  | Functional outcome Scale | n = 1 | Reported in one study |
|  | Sickness Impact Profile | n = 1 | Reported in one study |
|  | Glasgow Coma Scale | n = 1 | Reported in one study |
| EMS-centered measures | Response time | n = 108 | The preferred outcome measure, reported in 108 studies |
|  | On-scene time | n = 15 | Reported in fifteen studies |
|  | Call-to-arrival time | n = 10 | Reported in nine studies |
|  | Presenting rhythm | n =5 | Reported in five studies |
|  | Total prehospital time | n = 5 | Reported in five studies |
|  | Collapse-to-call time | n = 3 | Reported in three studies |
|  | Total transport time | n = 3 | Reported in three studies |
|  | Transport interval | n = 2 | Reported in two studies |
|  | Collapse-to-CPR time | n = 2 | Reported in two studies |
|  | Arrest-to-BLS time | n = 2 | Reported in two studies |
|  | Collapse-to-hospital time | n = 1 | Reported in one study |
|  | Population density | n = 1 | Reported in one study |
|  | Collapse-to-Defibrillation time | n = 1 | Reported in one study |
|  | CPR duration | n = 1 | Reported in one study |
|  | 3 mins. CPR first vs. Defibrillation | n = 1 | Reported in on study |
|  | Defibrillation time | n = 1 | Reported in one study |
|  | Time to dispatch | n = 1 | Reported in on study |
|  | Arrest-to-BLS time | n = 1 | Reported in one study |
|  | Call-to-door time | n = 1 | Reported in one study |
|  | Physician presence | n = 1 | Reported in one study |
|  | Resuscitation time | n = 1 | Reported in one study |
|  | Dispatch interval | n = 1 | Reported in one study |
|  |  |  |  |
|  |  |  |  |
|  |  |  |  |
| Legend: EMS: emergency medical services, 24H: 24 hours; CPR: cardiopulmonary resuscitation; BLS: basic life support | | | |

**Supplemental material Table 6: Overview of study characteristics of the included literature**

**Author/ year**

**Abrams 2011^1^**

**Afzali 2013^2^**

**Ahmoudi**

**2022^3^**

**Al-Dury 2020^4^**

**Alqudah 2021^5^**

**Alumran 2020^6^**

**Aziz 2020^7^**

**Azpiazu 2024^8^**

**Bagher 2017^9^**

**Baker 2008^10^**

**Berge 2005^12^**

**Biewener 2000^12^**

**Source**

Published

article

Published

article

Author contact

Published

conference

abstract

Published

article

Published

article

Published

article

Published

article

Author contact

Published

article

Published

article

Published

article

Published

article

Author contact

Published

article

**Study type**

Retrospective

observational

study

Prospective

observational

study

Prospective

observational

study

Retrospective

observational

study

Retrospective

observational

study

Retrospective

observational

study

Prospective

observational

study

Retrospective

study

Retrospective

observational

study

Randomized

controlled

study

Retrospective

observational

study

Retrospective

observational

study

**Country**

USA

DEN

UAE

SWE

AUS

KSA

MY

ESP

AUS

AUS

NOR

GER

**Setting**

Urban

Rural

Mixed

Urban

Rural

Urban

Metropolitan

Mixed

Urban

Urban

Mixed

Mixed

**Organization**

GEMS

HEMS

EMS

GEMS

GEMS

GEMS

GEMS

Mixed

GEMS

GEMS

HEMS

Mixed

**Population**

1,156

609

330

45,067

5,631

108

82

9,499

378

202

252

**122**

**Transport**

Ambulance

transport

Helicopter

transport

Ambulance

transport

Ambulance

transport

Ambulance

transport

Ambulance

transport

Ambulance

Transport

Ambulance

transport

Ambulance

transport

Ambulance

transport

Helicopter

transport

Ambulance

transport

**Outcomes**

Survival to

discharge

30-days

survival

Survival to

discharge

30-days

survival

Survival to

discharge;

Event survival

Unspecified

survival

Survival to

discharge

Survival to

discharge

90-days

survival

Survival to

discharge

24H, 1-year

survival

7-days

survival

**Study focus**

Predictors of survival in OHCA

Introduction

of HEMS service

Predictors of

survival in OHCA

Predictors of

survival in OHCA

Response time in traumatic OHCA

Predictors of

survival in OHCA

Predictors of

survival in OHCA

Predictors of

Survival in OHCA

Survival factors

In trauma

Defibrillation vs.

3 mins. CPR

Helicopter

transport of sick neonates

Diagnose and

Treatment in trauma

**Author/ year**

**Bjorkmann 2020^13^**

**Bjornsson 2006^14^**

**Blackwell 2009^15^**

**Blaengsdottir**

**1994^16^**

**Blanchard 2012^17^**

**Bossers 2021^18^**

**Brede 2020^19^**

**Brison 1992^20^**

**Brown 2019^21^**

**Bujak 2021^22^**

**Burger 2018^23^**

**Source**

Published

article

Author contact

Published

article

Published

article

Published

article

Published

article

Published

article

Published

article

Published

article

Published

article

Published

article

Published

article

**Study type**

Retrospective

observational

study

Retrospective

observational

study

Retrospective

case-control

study

Retrospective

observational

study

Retrospective

observational

study

Prospective

observational

study

Retrospective

observational

study

Retrospective

observational

study

Retrospective

observational

study

Retrospective

observational

study

Retrospective

observational

study

**Country**

FIN

ISL

USA

ISL

CAN

NL

NOR

CAN

AUS

POL

GER

**Setting**

Mixed

Urban

Metropolitan

Urban

Urban

Mixed

Mixed

Mixed

Metropolitan

Urban

Mixed

**Organization**

HEMS

GEMS

PEMS

GEMS

GEMS

Mixed

Mixed

GEMS

PEMS

GEMS

GEMS

**Population**

4,809

232

746

308

7 760

2 589

2 241

1 510

1 625

1 392

10 853

**Transport**

Helicopter

transport

Ambulance

transport

Ambulance

transport

Ambulance

transport

Ambulance

transport

Ambulance/

helicopter

transport

Ambulance/

helicopter

transport

Ambulance

transport

Ambulance

transport

Ambulance

transport

Ambulance

transport

**Outcomes**

30-days

survival

Survival to

discharge;

1-year survival

Survival to

discharge

Survival to

discharge

Survival to

discharge

30-days

survival

30-days

survival

Survival to

discharge

30-days

survival

Survival to

discharge

Survival to

discharge

**Study focus**

Prehospital time intervals in trauma

EMS CPR; by-stander CPR

in OHCA

Response time

>/< 11 mins. in

EMS

ACLS; bystander

effects in OHCA

Response time

>/< 8 mins. in

OHCA

GCS<8 as

predictor in

TBI

REBOA in

OHCA

Defibrillation

In OHCA

Prehospital time

in trauma

Predictors of

survival in OHCA

Response time

In OHCA

**Author/ year**

**Byrne 2019^24^**

**Cardoso 2014^25^**

**Chang 2015^26^**

**Chen 2015^27^**

**Chesters 2015^28^**

**Claesson 2008^29^**

**Deasy 2012^30^**

**deGraaf 2019^31^**

**Dicker 2018^32^**

**Dinh 2023^33^**

**Dyson 2013^34^**

**Einarsson 1989^35^**

**Source**

Published

article

Published

article

Published

article

Published

article

Published

article

Published

article

Published

article

Published

article

Published

article

Published

article

Published

article

Published

article

**Study type**

Retrospective

observational

study

Prospective

observational

study

Retrospective

observational

study

Retrospective

observational

study

Retrospective

observational

study

Retrospective

observational

study

Retrospective

observational

study

Retrospective

observational

study

Retrospective

observational

study

Retrospective

Observational study

Retrospective

observational

study

Retrospective

observational

study

**Country**

USA

BRA

KOR

TWN

UK

SWE

AUS

NL

NZ

AUS

AUS

SLO

**Setting**

Mixed

Metropolitan

Mixed

Metropolitan

Mixed

Urban

Mixed

Mixed

Mixed

Mixed

Mixed

Urban

**Organization**

EMS

HEMS

GEMS

EMS

Mixed

EMS

EMS

EMS

EMS

Mixed

EMS

MECU

**Population**

12,098

220

1,953

3,856

605

255

2,107

655

3,862

9,012

336

138

**Transport**

Ambulance

transport

Helicopter

transport

Ambulance

transport

Ambulance

transport

Ambulance/

helicopter

transport

Ambulance

transport

Ambulance

transport

Ambulance

transport

Ambulance

transport

Ambulance

transport

Ambulance

transport

Ambulance

transport

**Outcomes**

Crude mortality

24H-survival

CPC: survival

to discharge

Survival to

discharge

Survival to

discharge;

CPC

30-days

survival

Survival to

discharge

30-days

survival

30-days

survival

30-days

survival

Survival to

discharge

Survival to

discharge

**Study focus**

Response time

In MVC

Performance

Indicators in

trauma

Bystander CPR;

dispatch advice in

pediatric OHCA

Performance

Indicators in

OHCA

Performance

indicators in

OHCA

Performance

Indicators in

drowning

Performance

Indicators in

Traumatic OHCA

On-scene time

In OHCA

Performance

Indicators in

OHCA

Protocol

Impact in

trauma

Performance

indicators in

drowning

MECU perfor-

mance; bystander

CPR

**Author/ year**

**Eisenburger**

**2001^36^**

**Ong**

**2003^37^**

**Fake 2013^38^**

**Finney 2023^39^**

**Fothergill 2021^40^**

**Fraga-Sastrias**

**2009^41^**

**Gnesin 2021^42^**

**Goh 2018^43^**

**Goh 2013^44^**

**Gregers 2021^45^**

**Grmec 2009^46^**

**Source**

Published

article

Published

article

Published

article

Published

article

Author contact

Published

article

Author contact

Published

article

Published

article

Author contact

Published

article

Published

article

Published

abstract

Author contact

Published

article

**Study type**

Retrospective

observational

study

Prospective

observational

study

Retrospective

observational

study

Retrospective

observational

study

Retrospective

observational

study

Prospective

observational

study

Retrospective

observational

study

Retrospective

observational

study

Retrospective

observational

study

Retrospective

observational

study

Retrospective

observational

study

**Country**

AUT

SG

NZ

UK

UK

MEX

DEN

USA

SG

DEN

SLO

**Setting**

Mixed

Metropolitan

Mixed

Mixed

Metropolitan

Mixed

Mixed

Mixed

Metropolitan

Mixed

Mixed

**Organization**

GEMS

GEMS

EMS

GEMS

PEMS

EMS

GEMS

GEMS

GEMS

EMS

GEMS

**Population**

368

548

413

1,614

3,122

255

3,548

654

2,375

16,670

788

**Transport**

Ambulance

transport

Ambulance

transport

Ambulance

transport

Ambulance

transport

Ambulance

transport

Ambulance

transport

Ambulance

transport

Ambulance

transport

Ambulance

transport

Ambulance

transport

Ambulance

transport

**Outcomes**

Survival to

discharge;

1-year survival

Survival to

discharge

Survival to

discharge

ROSC at

arrival to

hospital

30-days

survival

Survival to

discharge;

CPC

30-days

survival

Unspecified

survival

30-days

survival

30-days

survival

Survival to

Discharge;

CPC

**Study focus**

Performance

Indicators in

alpine OHCA

Performance

Indicators in

OHCA

Location/

socioeconomic

factors in OHCA

Urban vs. rural location in OHCA

COVID-19

effects in OHCA

Performance

indicators in

OHCA

Time to dispatch in OHCA

Nearby medical

facility in OHCA

Location in OHCA

focus

Bystander CPR

in OHCA focus

Performance

Indicators in

OHCA

**Author/ year**

**Gunaga 2020^47^**

**Han 2022^48^**

**Hayes 2010^49^**

**Henry 2013^50^**

**Herlitz 2006^51^**

**Herlitz 2008^52^**

**Hillis 1993^53^**

**Holmen 2020^54^**

**Huabbangyang**

**2021^55^**

**Hubert 2016^56^**

**Jennings 2006^57^**

**Source**

Published

article

Published

conference

abstract

Published

conference

abstract

Published

article

Published

article

Published

article

Published

article

Published

article

Published

article

Published

article

Published

article

Author contact

**Study type**

Retrospective

observational

study

Retrospective

observational

study

Retrospective

observational

study

Retrospective

observational

study

Retrospective

observational

study

Retrospective

observational

study

Prospective

cohort study

Retrospective

observational

study

Retrospective

observational

study

Prospective

observational

study

Retrospective

observational

study

**Country**

USA

KOR

IRL

IRL

SWE

SWE

CAN

SWE

THA

FRA

AUS

**Setting**

Suburban

Urban

Mixed

Mixed

Mixed

Mixed

Urban

Mixed

Mixed

Rural

Mixed

**Organization**

GEMS

GEMS

EMS

GEMS

GEMS

GEMS

GEMS

GEMS

GEMS

Mixed

GEMS

**Population**

769

3,289

74

231

4,667

22,465

157

20,420

273

234

1,790

**Transport**

Ambulance

transport

Ambulance

transport

Ambulance

transport

Ambulance

transport

Ambulance

transport

Ambulance

transport

Ambulance

transport

Ambulance

transport

Ambulance

transport

Ambulance

transport

Ambulance

transport

**Outcomes**

Survival to

discharge

Survival to

discharge

Survival to

discharge

Survival to

discharge

30-days

survival

30-days

survival

Survival to

discharge

30-days

survival

On-scene

ROSC

Survival to

Discharge;

CPC

Survival to

discharge

**Study focus**

Private vs. public

EMS delivery

in OHCA

Response time

second unit in

OHCA

Performance

indicators in

OHCA

Performance

indicators in

OHCA

Time to call

for ambulance

in OHCA

Predictors of

Survival in OHCA

Defibrillation and

airway manage-

ment in OHCA

Response time

in OHCA

Predictors of

ROSC in OHCA

Performance

Indicators and

TOR in drowning

Predictors of

Survival in

OHCA

**Author/ year**

**Jeong 2017^58^**

**Kennedy 2023^59^**

**Kentsch 2000^60^**

**Kitano 2022^61^**

**Klosiewicz 2017^62^**

**Lee 2019^63^**

**Lee 2013^64^**

**Leung 2001^65^**

**Lim 2020^66^**

**Lin 2014^67^**

**Little 2020^68^**

**Source**

Published

conference

abstract

Published

article

Published

article

Published

article

Published

article

Author contact

Published

article

Published

article

Published

article

Published

article

Published

conference

abstract

Published

article

**Study type**

Retrospective

observational

study

Interrupted

time-series

analysis

Retrospective

observational

study

Retrospective

observational

study

Retrospective

observational

study

Prospective

observational

study

Retrospective

observational

study

Prospective

observational

study

Retrospective

observational

study

Retrospective

observational

study

Retrospective

observational

study

**Country**

KOR

AUS

GER

JPN

POL

KOR

TWN

HK

SG

TWN

UK

**Setting**

Urban

Mixed

Mixed

Mixed

Mixed

Mixed

Metropolitan

Metropolitan

Urban

Metropolitan

Mixed

**Organization**

EMS

EMS

EMS

EMS

Mixed

GEMS

EMS

EMS

GEMS

EMS

GEMS

**Population**

400

5,034

326

3,883

511

2,309

515

329

25,895

2,156

1,790

**Transport**

Ambulance

transport

Ambulance

transport

Ambulance

transport

Ambulance

transport

Ambulance

transport

Ambulance

transport

Ambulance

transport

Ambulance

transport

Ambulance

transport

Ambulance

transport

Ambulance

transport

**Outcomes**

Survival to

discharge

CPC

Survival to

discharge

Survival to

discharge

CPC

30-days

survival

On-scene

ROSC

Survival to

discharge

CPC

Survival to

discharge

Survival to

discharge

Survival to

discharge

Survival to

discharge

Survival to

discharge

**Study focus**

Performance

Indicators in

OHCA

COVID-19

related effects

in OHCA

Performance

indicators in

OHCA

EMS witnessed vs.

unwitnessed

traumatic OHCA

Performance

indicators

in OHCA

Response time

in OHCA

5:1 vs. continuous

compressions

in OHCA

Performance

indicators

in OHCA

Predictors of

survival in

OHCA

Presenting

rhythm in

OHCA

COVID-19

Influence in

STEMI to PCI

**Author/ year**

**Liu 2023^69^**

**Lyon 2013^70^**

**Margey 2011^71^**

**Mathiesen 2018^72^**

**Mayer 1979^73^**

**Meyer 2001^74^**

**Mickiewicz 2023^75^**

**Mikkelsen 2017^76^**

**Mills 2023^77^**

**Mills 2019^78^**

**Mogensen 2015^79^**

**Morrisey 1996^80^**

**Source**

Published

article

Published

article

Published

article

Published

article

Published

article

Published

article

Published

article

Published

article

Published

article

Published

article

Published

article

Published

article

**Study type**

Retrospective

observational

study

Retrospective

observational

study

Retrospective

observational

study

Retrospective

observational

study

Retrospective

observational

study

Retrospective

observational

study

Retrospective

Observational

study

Retrospective

observational

study

Retrospective

observational

study

Retrospective

observational

study

Retrospective

observational

study

Retrospective

observational

study

**Country**

TWN

UK

IRL

NOR

USA

AUS

POL

DEN

DEN

DEN

ISL

USA

**Setting**

Mixed

Rural

Mixed

Mixed

Mixed

Metropolitan

Rural

Mixed

Mixed

Mixed

Mixed

Rural

**Organization**

GEMS

HEMS

Mixed

Mixed

GEMS

GEMS

Mixed

MECU

GEMS

EMS

Mixed

EMS

**Population**

1,064

64

962

1,138

535

778

74

32,873

182,895

19,968

289

2,550

**Transport**

Ambulance

transport

Helicopter

transport

Ambulance

transport

Ambulance

transport

Ambulance

transport

Ambulance

transport

Ambulance

transport

Ambulance

transport

Ambulance

transport

Ambulance

transport

Ambulance

transport

Ambulance

transport

**Outcomes**

Survival to

discharge

Survival to

discharge

Survival to

discharge

Survival to

discharge

Survival to

discharge

Survival to

discharge

Survival to

discharge

30-days

survival

30-days

survival

30-days

survival

Survival to

Discharge

30-days

survival

**Study focus**

COVID-19

related effects

in OHCA

HEMS response

effects in

OHCA

Predictors of

indicators in

OHCA

Predictors of

indicators in

OHCA

Response time

in OHCA

Performance

indicators in

OHCA

Performance

Indicators in

Rural OHCA

Response time

in MECU served

population

Response time

in general

population

Prehospital

time in general

population

Performance

Indicators in

OHCA

Performance

Indicators in

rural trauma

**Author/ year**

**Nadolny 2021^81^**

**Naroo 2012^82^**

**Navab 2019^83^**

**Nichol 2016^84^**

**Nielsen 2021^85^**

**O’Keefe 2011^86^**

**Park 2017^87^**

**Pell 2001^88^**

**Pons 2005^89^**

**Pons 2002^90^**

**Puolakka 2023^91^**

**Source**

Published

article

Published

conference

abstract

Published

Published

article

Published

article

Author contact

Published

article

Published

article

Published

article

Published

article

Published

article

Published

article

**Study type**

Retrospective

observational

study

Retrospective

observational

study

Retrospective

observational

study

Retrospective

observational

study

Retrospective

observational

study

Retrospective

observational

study

Retrospective

observational

study

Retrospective

observational

study

Retrospective

observational

study

Retrospective

observational

study

Retrospective

observational

study

**Country**

POL

UAE

IRI

USA

DEN

UK

KOR

SCO

USA

USA

FIN

**Setting**

Mixed

Metropolitan

Mixed

Metropolitan

Mixed

Mixed

Mixed

Mixed

Mixed

Mixed

Mixed

**Organization**

EMS

GEMS

GEMS

GEMS

HEMS

GEMS

EMS

GEMS

GEMS

GEMS

EMS

**Population**

218

60

3,214

2,687

525

778

15,354

10,554

9,559

3,490

73

**Transport**

Ambulance

transport

Ambulance

transport

Ambulance

transport

Ambulance

transport

Helicopter

transport

Ambulance

transport

Ambulance

transport

Ambulance

transport

Ambulance

transport

Ambulance

transport

Ambulance

transport

**Outcomes**

Survival to

discharge

30-days

survival

Survival to

discharge

Survival to

discharge

30-days

survival

Survival to

discharge

Survival to

discharge;

CPC

Survival to

discharge

Survival to

discharge

Survival to

discharge

Survival to

Discharge;

CPC

**Study focus**

Primary GCS

as predictor

in OHCA

Response time

In OHCA

Performance

indicators in

OHCA

Activation time

In OHCA

Characteristics

of pediatric

population

Response time

in OHCA

Response time

in OHCA

Response time

in OHCA

Response time

in OHCA

Response time

in trauma

ECPR protocol

in OHCA

**Author/ year**

**Do 2010^92^**

**Rajan 2016^93^**

**Renkiewicz**

**2014^94^**

**Schinnerl 1990^95^**

**Semensato**

**2011^96^**

**Shah 2010^97^**

**Shepherd 2008^98^**

**Sigursson 2000^99^**

**Sipria 2016^100^**

**Spaite 2008^101^**

**Stoeckl 2010^102^**

**Source**

Published

article

Published

Published

Published

article

Published

article

Published

article

Published

article

Published

article

Published

article

Published

article

Published

conference

abstract

**Study type**

Retrospective

observational

study

Retrospective

observational

study

Retrospective

observational

study

Retrospective

observational

study

Retrospective

observational

study

Retrospective

observational

study

Retrospective

observational

study

Retrospective

observational

study

Prospective

observational

study

Retrospective

observational

study

Retrospective

observational

study

**Country**

DEN

DEN

USA

AUT

BRA

UK

AUS

ISL

EST

USA

AUT

**Setting**

Mixed

Mixed

Mixed

Mixed

Mixed

Metropolitan

Rural

Mixed

Mixed

Mixed

Mixed

**Organization**

MECU

EMS

EMS

MECU

EMS

EMS

HEMS

MECU

EMS

EMS

EMS

**Population**

2,432

7,623

599

89

593

3,181

222

308

3,335

1,177

552

**Transport**

Ambulance

transport

Ambulance

transport

Ambulance

transport

Ambulance

transport

Helicopter

transport

Ambulance

transport

Helicopter

transport

Ambulance

transport

Ambulance

transport

Ambulance

transport

Ambulance

transport

**Outcomes**

ROSC at

hospital

admission

30-days

survival

ROSC at

hospital

admission

Survival to

discharge

30-days

Survival; to

discharge

Survival to

discharge

Unspecified

death

Survival to

discharge

Survival to

discharge;

CPC

Survival to

discharge

Survival to

Discharge;

CPC

**Study focus**

Response time

In OHCA

Bystander CPR

Effect in OHCA

Response time

association w/

shockable rhythm

Predictors of

Survival in

OHCA

Performance

Indicators in

OHCA

Ethnicity effects

in OHCA

Performance

Indicators in

rural trauma

Performance

indicators in

OHCA

Performance

indicators in

OHCA

Performance

Indicators in

OHCA

Dispatch time

effects in

OHCA

**Author/ year**

**Stromsoe 2015^103^**

**Stromsoe 2011^104^**

**Sugiyama 2023^105^**

**Syvaoja 2018^106^**

**TerAvest 2019^107^**

**Thompson**

**2017^108^**

**Trenkler 2012^109^**

**Tsai 2017^110^**

**Wai 2005^111^**

**Weinlich 2019^112^**

**Wik 2003^113^**

**Wissa 2021^114^**

**Zheng 2023^115^**

**Source**

Published

article

Author contact

Published

article

Author contact

Published

Published

article

Published

article

Published

article

Published

conference

abstract

Published

article

Published

article

Published

article

Published

article

Published

Article

Published

Article

**Study type**

Retrospective

observational

study

Retrospective

observational

study

Retrospective

observational

study

Retrospective

observational

study

Retrospective

observational

study

Retrospective

observational

study

Retrospective

observational

study

Retrospective

observational

study

Retrospective

observational

study

Retrospective

observational

study

Randomized

controlled

trial

Retrospective

observational

study

Prospective

Observational

study

**Country**

SWE

SWE

JPN

FIN

UK

UK

SLO

TWN

HK

GER

NOR

AUS

CHI

**Setting**

Mixed

Mixed

Mixed

Mixed

Mixed

Mixed

Mixed

Mixed

Metropolitan

Mixed

Mixed

Mixed

Mixed

**Organization**

EMS

EMS

EMS

EMS

HEMS

EMS

EMS

EMS

EMS

HEMS

EMS

EMS

MECU

**Population**

59,926

9,979

3,367

2,054

263

1,033

2,906

546

124

1,646

200

502

38,227

**Transport**

Ambulance

transport

Ambulance

transport

Ambulance

transport

Ambulance

transport

Helicopter

transport

Ambulance

transport

Ambulance

transport

Ambulance

transport

Ambulance

transport

Helicopter

transport

Ambulance

transport

Ambulance

Transport

Ambulance Transport

**Outcomes**

30-days

survival;

CPC

30-days

survival

Survival to

discharge;

CPC

Survival to

discharge

Survival to

discharge

Survival to

discharge

30-days

survival

Survival to

discharge

Survival

to discharge

Survival to

discharge;

GOS

Survival to

discharge;

CPC

Survival to

Discharge

Survival to

Discharge

**Study focus**

Performance

Indicators in

OHCA

Performance

Indicators in

OHCA

COVID-19 effects

On EMS in

OHCA

Dispatch

Recognition in

OHCA

Performance

Indicators in

traumatic OHCA

Performance

indicators in

major trauma

Performance

Indicators

OHCA

Performance

Indicators in

OHCA

Performance

Indicators in

OHCA

Performance

Indicators in

trauma

CPR vs. defibrillation

In OHCA

Private vs. public

EMS delivery

in OHCA

Performance

Indicators in

OHCA

**Legend:**

**Medical abbreviations**: ACLS: Advanced cardiac life support; CAD: cardiac arrest after drowning; COVID-19: Corona virus disease 2019; CPR: cardio-pulmonary resuscitation ECPR: Extracorporeal cardiopulmonary resuscitation; EMDC: Emergency medical dispatch centre; EMS: Emergency medical service; GCS: Glasgow Coma Scale; GEMS: Ground EMS; HEMS: Helicopter EMS; MECU: Mobile emergency care unit; MICU: Mobile intensive care unit; MVC: motor vehicle crash; OHCA: Out of hospital cardiac arrest;; PEMS: Paramedic staffed EMS; PPCI: Primary percutaneous coronary intervention;; REBOA: resuscitative endovascular balloon occlusion of the aorta; ROSC: Return of spontaneous circulation; STEMI: ST-elevation myocardial infarction; TBI: Traumatic brain injury; TOR: Termination of resuscitation; 24H: 24 hours;

**Origin abbreviations**: AUS: Australia; AUT: Austria; BRA: Brazil; CHI: People’s Republic of China; DEN: Denmark; ESP: Spain: EST: Estonia; FIN: Finland; FRA: France; GER: Germany; HK: Hong Kong; IRI: Iran; IRL: Ireland; ISL: Iceland; JPN: Japan; KSA: Saudi Arabia; KOR: South Korea; MY: Malaysia; NOR: Norway; NZ: New Zealand; POL: Poland; SCO: Scotland; SG: Singapore; SLO: Slovakia; SWE: Sweden TWN: Taiwan; UAE: United Arab Emirates; UK: United Kingdom; USA; United States of America

**Supplemental Table 7. Results of the individual studies**

**Author/year Outcome results**

**Abrams 2011^1^** Patient-centered measures:

- Survival to discharge rate 11.1%
- Survival to discharge OR 2.10 (1.20; 3.60)

EMS-centered measures:

- Mean response time: N/A, composite

**Afzali 2013^2^** Patient-centered measures:

- 30-day survival rate 93.9%;
- 14.1% admitted to ICU
- Response time association with survival not assessed

EMS-centered measures:

- Mean response time: 16 minutes

**Ahmoudi 2022^3^** Patient-centered measures:

- Survival to discharge rate 4.85%
- Response time association with survival not assessed

EMS-centered measures:

- Mean response time 10.15±4.92 minutes.

**Al-Dury 2020^4^** Patient-centered measures:

- 30-days survival: N/A
- Response time association with survival not assessed

EMS-centered measures:

- Median response time 10,0 (IQR 6.9 - 15.0)

**Alqudah 2021^5^** Patient-centered measures:

- Survival to discharge rate 2.7%
- Survival to discharge OR 0.96 (0.89; 1.03)

EMS-centered measures:

- Mean response time 9.8 minutes.

**Alumran 2020^6^** Patient-centered measures:

- Survival to discharge 21.3%
- Survival to discharge OR 2.40 (0.81; 7.10), p-value 0.106

EMS-centered measures:

- Mean response time 8.5±5.1 minutes

**Aziz 2020^7^** Patient-centered measures:

- Survival to discharge rate 1.2%
- Response time association with survival not assessed

EMS-centered measures:

- Mean response time 14.9 minutes.

**Azpiazu 2024^8^** Patient-centered measures:

- Survival to discharge rate 11.5%
- Response time association with survival not assessed

EMS-centered measures:

- Median response time 11,9 (IQR 8.2 - 17;5)

**Bagher 2017^9^** Patient-centered measures:

- 90-days OR 1.70 (0.90; 3.30), p-value 0.13
- 90-days survival rate 89.0%

EMS-centered measures:

- Median response time 8 (IQR 6-12)

**Baker 2008^10^** Patient-centered measures:

- Survival to discharge rate 18% with defib. vs. 10% in CPR before defib.
- Survival to discharge OR 0.56 (0.25; 1.25), p-value 0.16

EMS-centered measures:

- Median response time 8.14 (IQR 7.39-8.49)

**Berge 2005^11^** Patient-centered measures:

- 24H survival rate 5.16%
- 1-year survival 12%
- Response time association with survival not assessed

EMS-centered measures:

- Mean response time 42 minutes.

**Biewener 2000^12^** Patient-centered measures:

- Survival to discharge rate: N/A
- Response time association with survival not assessed

EMS-centered measures:

- Mean response time 8.1±5.9 minutes

**Bjorkmann2020^13^** Patient-centered measures:

- 30-day survival rate 87.9%
- Survival to discharge OR 0.71 (0.29; 1.29)

EMS-centered measures:

- Median response time 11 (IQR 8-19) for EMS vs. 26 (IQR 17-41) for HEMS

**Bjornsson 2006^14^** Patient-centered measures:

- Survival to discharge rate 19.0%
- Response time association with survival not assessed

EMS-centered measures:

- Mean response time 6.1 minutes.

**Blackwell 2009^15^** Patient-centered measures:

- Survival to discharge rate 80% at response time > 11 mins. vs. 82% at < 11 mins.
- Response time association with survival not assessed

EMS-centered measures:

- Median response time: 12.40 vs. 06.32 minutes

**Blaengsdottir^16^** Patient-centered measures:

- Survival to discharge rate 17%
- Survival to discharge OR 1.02 (1.01; 1.72)

EMS-centered measures:

- Mean response time 4.6 minutes.

**Blanchard 2012^17^** Patient-centered measures:

- 30-days survival rate 93.6% <8 minutes vs. 92.9% > 8 minutes response time
- Survival to discharge OR 1.02 (0.99; 1.05), p-value 0.285

EMS-centered measures:

- Mean response time NR

**Bossers 2021^18^** Patient-centered measures:

- 30-days survival rate 61.0%
- Response time association with survival not assessed

EMS-centered measures:

- Median response time 18 (IQR 13-23)

**Brede 2020^19^** Patient-centered measures:

- 30-day Survival rate 7-64% between groups
- Response time association with survival not assessed

EMS-centered measures:

- Mean response time: N/A

**Brison 1992^20^** Patient-centered measures:

- Survival to discharge rate 2.5%
- Response time association with survival not assessed

EMS-centered measures:

- Mean response time 7.8 minutes

**Brown 2020^21^** Patient-centered measures:

- 30-days survival rate: N/A
- 30-days survival OR 1.00 (0.64; 1.59)

EMS-centered measures:

- Median response time 10.0 vs. 13.0

**Bujak 2021^22^** Patient-centered measures:

- Survival to discharge rate 9.2%
- Survival to discharge OR 0.76 (0.66; 0.87), p-value <0.001

EMS-centered measures:

- Median response time 8 (IQR 6-11)

**Burger 2018^23^** Patient-centered measures:

- Survival to discharge rate: 12.9% vs. 6.4% at 1.10 vs. 9.47 minutes
- Survival to discharge OR 0.75 (0.69; 0.82), p-value <0.001

EMS-centered measures:

- Mean response time 1.10/9.47 minutes

**Byrne 2019^24^** Patient-centered measures:

- Survival to discharge rate N/A
- Survival to discharge OR 1.95 (1.72; 2.22)

EMS-centered measures:

- Median response time 9 (IQR 7-11) minutes

**Cardoso 2014^25^** Patient-centered measures:

- Survival to discharge rate 84.1%
- Response time association with survival not assessed

EMS-centered measures:

- Mean response time 10±4 minutes

**Chang 2018^26^** Patient-centered measures:

- Survival to discharge rate 8.65%
- Survival to discharge OR 2.52 (0.91; 6.97)

EMS-centered measures:

- Median response time 6 (IQR 5-9)

**Chen 2015^27^** Patient-centered measures:

- Survival to discharge rate 5.0%
- Survival to discharge OR 2.12 (1.40; 3.23). p-value <0.001

EMS-centered measures:

- Mean response time 6.5 minutes

**Chesters 2015^28^** Patient-centered measures:

- Survival to discharge rate 11.7%
- CPC 1 or 2 in 86%
- Response time association with survival not assessed

EMS-centered measures:

- Mean response time 29.5 minutes

**Claesson 2008^29^** Patient-centered measures:

- Survival to discharge rate 8.8% in OHCA vs. 11.5% in drowning
- Response time association with survival not assessed

EMS-centered measures:

- Mean response time 6 (IQR 4-19) OHCA vs. 9 (IQR 5-18) drowning

**Deasy 2012^30^** Patient-centered measures:

- Survival to discharge rate 5.1%
- Response time association with survival not assessed

EMS-centered measures:

- Median response time 8 (IQR 6-11)

**deGraaf 2019^31^** Patient-centered measures:

- 30-days survival rate 4.0%
- 30-days survival OR 0.85 (0.76; 0.96), p-value 0.007

EMS-centered measures:

- Median response time 12 (IQR 9-15)

**Dicker 2018^32^** Patient-centered measures:

- 30-days survival rate 15%
- 30-days survival OR 0.43 (0.21; 0.88), p-value <0.001

EMS-centered measures:

- Median response time 9 (IQR 7-12).

**
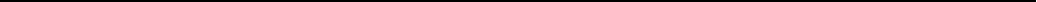
**

**Dinh 2023^33^** Patient-centered measures:

- 30-days survival rate 98.49%
- 30-days mortality OR 1.00 (0.99; 1.01)

EMS-centered measures:

- Median response time 15 (IQR 11 – 23)

**Dyson 2013^34^** Patient-centered measures:

- Survival to discharge rate 8%
- Survival to discharge OR 0.73 (0.54; 0.98)

EMS-centered measures:

- Median response time 8 (IQR 6-12)

**Einarsson 1989^35^** Patient-centered measures:

- Survival to discharge rate 17%
- Response time association with survival not assessed

EMS-centered measures:

- Mean response time 5.0 minutes

**Eisenburger^36^** Patient-centered measures:

- 1-year survival rate 8%
- Response time association with survival not assessed

EMS-centered measures:

- Mean response time 8 minutes

**Ong^37^** Patient-centered measures:

- Survival to discharge rate 2.0%
- Response time association with survival not assessed

EMS-centered measures:

- Mean response time 10.2$\pm$4.3 minutes

**Fake 2013^38^** Patient-centered measures:

- Survival to discharge rate 12.6%
- Response time association with survival not assessed

EMS-centered measures:

- Mean response time 9.5±4.9 minutes

**Finney 2023^39^** Patient-centered measures:

- ROSC at hospital 27.3% rural vs. 31.6% urban
- Response time association with survival not assessed

EMS-centered measures:

- Mean response time 10.43±8.2 rural vs. 7.35±7.1 urban

**Fothergill 2021^40^** Patient-centered measures:

- 30-days survival rate 4.4% in COVID-19 vs. 10.6% in non-COVID-19
- Response time association with survival not assessed

EMS-centered measures:

- Median response time 9.3 (IQR 6.4-15.3) COVID vs. 7.2 (IQR 5.4-9.8) non-COVID

**Fraga-Sastrias^41^** Patient-centered measures:

- Survival to discharge rate 0.0%
- Response time association with survival not assessed

EMS-centered measures:

- Mean response time 13.6±10.4 minutes

**Gnesin 2021^42^** Patient-centered measures:

- Survival to discharge rate 12.0% low priority vs. 13.7% high
- Response time association with survival not assessed

EMS-centered measures:

- Median response time 9.3 (IQR 7.0-12.2)

**Goh 2018^43^** Patient-centered measures:

- Survival to discharge 31.0 %
- Response time association with survival not assessed

EMS-centered measures:

- Median response time 7.8 (IQR 5.2-10.2)

**Goh 2013^44^** Patient-centered measures:

- Survival to discharge rate 0.9 vs. 2.7%
- Survival to discharge OR 1.00 (1.00; 1.00)

EMS-centered measures:

- Median response time 8.55 (IQR 6.51-11.18) vs. 09.04 (IQR 6.35-11.59)

**Gregers 2021^45^** Patient-centered measures:

- 30-day survival rate 13.00%
- Survival to discharge OR 1.22 ( 1.08; 1.38)

EMS-centered measures:

- Mean response time 6/7/8 minutes

**Grmec 2009^46^** Patient-centered measures:

- Survival to discharge rate 44% in drowning vs. 22% in OHCA
- Response time association with survival not assessed

EMS-centered measures:

- Mean response time 11 vs. 6 minutes

**Gunaga 2020^47^** Patient-centered measures:

- Survival to discharge rate 98.58 private vs. 98.09% municipal
- Response time association with survival not assessed

EMS-centered measures:

- Mean response time 8.1 municipal vs. 3.9 municipal

**Han 2022^48^** Patient-centered measures:

- Survival to discharge rate 12.6% in <14 mins. vs. 6.3% in > 14. Mins. RT
- Survival to discharge OR 2.47 (1.60; 3.87), p-value <0.001

EMS-centered measures:

- Mean response time N/A

**Hayes 2010^49^** Patient-centered measures:

- Survival to discharge 3%
- Response time association with survival not assessed

EMS-centered measures:

- Mean response time 9±4 minutes

**Henry 2013^50^** Patient-centered measures:

- Survival to discharge 7.4%/7.4%
- Response time association with survival not assessed

EMS-centered measures:

- Median response time 16.5 (IQR 11-23.5) vs. 9 (IQR 7-12) minutes

**Herlitz 2006^51^** Patient-centered measures:

- 30-day survival rate 5%
- Survival to discharge OR 3.25 (2.66; 3.99)

EMS-centered measures:

- Median response time 10 minutes

**Herlitz 2008^52^** Patient-centered measures:

- 30-day survival rate1.3%
- Survival to discharge OR 2.00 (0.72; 2.24), p-value <0.0001

EMS-centered measures:

- Median response time 13.0 minutes

**Hillis 1993^53^** Patient-centered measures:

- Survival to discharge rate 3.3% vs. 6.3% with defibrillation
- Response time association with survival not assessed

EMS-centered measures:

- Mean response time 5.8±3.7 vs. 5.7±3.6 minutes

**Holmen 2020^54^** Patient-centered measures:

- 30-days survival rate 19.5% (0-6 min. RT) vs 9.4% (>10 mins. RT)
- 30-days survival OR 0.69 (0.60; 0.79)

EMS-centered measures:

- Median response time 5 (IQR 4-6) – 21 (IQR 18-26) minutes

**Huabbangyang 2021^55^**

Patient-centered measures:

- Survival to hospital 25.6%
- Response time association with survival not assessed

EMS-centered measures:

- Mean response time 12.87±7.30

**Hubert 2016^56^** Patient-centered measures:

- Survival to discharge 9.0%
- Survival to discharge OR 0.96 (0.93; 1.00), p-value 0.031

EMS-centered measures:

- Mean response time 22 (IQR 15.30)

**Jennings 2006^57^** Patient-centered measures:

- Survival to discharge rate 7.1%
- Response time association with survival not assessed

EMS-centered measures:

- Median response time 7 (IQR 6-9) urban vs. 8 (IQR 6-11) minutes

**Jeong 2017^58^** Patient-centered measures:

- Survival to discharge rate 9.5%
- Favorable neurologic status at discharge rate 5.3%
- Survival to discharge OR 0.74 (0.25; 2.00), p-value 0.74

EMS-centered measures:

- Median response time 8 (IQR 6-10) minutes

**Kennedy 2023^59^** Patient-centered measures:

- Survival to discharge 30.8%
- Response time association with survival not assessed

EMS-centered measures:

- Median response time 10.6 (IQR 7.7.-14.8) minutes.

**Kentsch 2000^60^** Patient-centered measures:

- Survival to discharge rate 3.7% (1989) vs. 8.1% (after 1989)
- Response time association with survival not assessed

EMS-centered measures:

- Mean response time 11.0±1.4 vs. 9.0±0.4 minutes

**Kitano 2022^61^** Patient-centered measures:

- 30-day survival rate 10.9% in EMS witnessed OHCA vs. 7.2% (bystander) vs. 5.6% (unwitnessed)
- 30-days survival OR 1.00 (1.00; 1.00), p-value < 0.01

EMS-centered measures:

- Median time from injury to cardiac arrest 18.0 minutes.

**Klosiewicz 2017^62^** Patient-centered measures:

- Survival to discharge rate N/A
- Response time association with survival not assessed

EMS-centered measures:

- Median response time 8.53 minutes.

**Lee 2019^63^** Patient-centered measures:

- Survival to discharge rate 11.2%
- Survival to discharge OR 1.08 (CI 1.04; 1.22) with response time decreased 1 minute
- Favorable neurological outcome OR 1.14 (CI 1.07;1.21)
- Survival to discharge w/ favorable neurological outcome OR 1.14 (1.07; 1.21), p-value <0.001

EMS-centered measures:

- Mean response time 9.28 ±8.27 minutes.

**Lee 2013^64^** Patient-centered measures:

- 10.1% w/ continuous compressions. 4.2% vs. 5:1 compression to ventilation rate
- Survival to discharge OR 2.43 (CI 1.15; 5.12)

EMS-centered measures:

- Mean response time 3.7±2.0 vs. 4.5±2.4 minutes

**Leung 2001^65^** Patient-centered measures:

- Survival to discharge rate 1.25%
- Response time association with survival not assessed

EMS-centered measures:

- Average total prehospital time 27.55 minutes.

**Lim 2020^66^** Patient-centered measures:

- Survival to discharge rate 4.0% Singapore vs. 13.6% Victoria
- Survival to discharge OR 0.91 (0.87; 0.94), p-value <0.0001

EMS-centered measures:

- Mean response time 11.5±4.8 vs. 12.2±11.2

**Lin 2014^67^** Patient-centered measures:

- Survival to discharge rate 3.8%
- Response time association with survival not assessed

EMS-centered measures:

- Mean response time 6.0 minutes.

**Little 2020^68^** Patient-centered measures:

- Survival to discharge rate 89.1% (2020) vs. 91.4 (2019)
- Response time association with survival not assessed

EMS-centered measures:

- Mean call-to-door time 87 (2020) vs. 75 (2019) minutes

**Liu 2023^69^** Patient-centered measures:

- Survival to discharge rate 5.29% pre-COVID vs. 2.21% COVID
- Survival to discharge OR 0.93 (0.87; 1.00), p-value 0.055

EMS-centered measures:

- Median response time 4 (IQR 3-5) vs. 5 (IQR 3-6) minutes

**Lyon 2013^70^** Patient-centered measures:

- Survival to discharge rate 6.3%
- Response time association with survival not assessed

EMS-centered measures:

- Median response time 17.0 minutes.

**Margey 2011^71^** Patient-centered measures:

- Survival to discharge rate improved 21.4% to 33% due to improved care
- Response time association with survival not assessed

EMS-centered measures:

- Mean response time decreased from 9.18 to 8.34 minutes

**Mathiesen^72^** Patient-centered measures:

- Survival to discharge rate 14.8% rural vs. 20.7% urban
- Survival to discharge OR 0.61 (0.44; 0.84) with response time > 10 minutes, p-value 0.002

EMS-centered measures:

- Median response time 11 (IQR 7-16) vs. 9 (IQR 7-12)

**Mayer 1979^73^** Patient-centered measures:

- Survival rate 30.2%
- Response time association with survival not assessed

EMS-centered measures:

- Mean response time N/A

**Meyer 2001^74^** Patient-centered measures:

- Survival to discharge rate 0.12%
- Response time association with survival not assessed

EMS-centered measures:

- Mean response time 9.76 minutes.

**
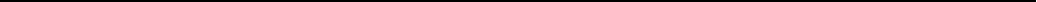
**

**Mikiewicz 2023^75^** Patient-centered measures:

- Survival to discharge rate 12.0%
- Response time association with survival not assessed

EMS-centered measures:

- CA to arrival time 19.0 mins (13.0; 26.0)

**Mikkelsen 2017^76^** Patient-centered measures:

- 30-days survival rate 94.3%
- 30-days survival OR 0.99 (0.97; 1.01), p-value 0.178

EMS-centered measures:

- Median response time 8 (IQR 5-12)

**Mills 2023^77^** Patient-centered measures:

- 30-days survival rate 23.9-95.9
- 30-days survival OR 1.00 (0.99; 1.00)

EMS-centered measures:

- Median response time 6.9-8.9 minutes

**Mills 2019^78^** Patient-centered measures:

- 30-day survival rate 81.7%
- 30-days mortality OR 0.76 (0.62; 0.94)

EMS-centered measures:

- Median response time 9.0 (IQR 5-13)

**Mogensen^79^** Patient-centered measures:

- Survival to discharge rate 25%
- Response time association with survival not assessed

EMS-centered measures:

- Mean response time 6.3 minutes

**Morrisey 1996^80^** Patient-centered measures:

- Survival to discharge 95.18%
- Response time association with survival not assessed

EMS-centered measures:

- Mean response time 8.5 minutes

**Nadolny 2021^81^** Patient-centered measures:

- Survival to discharge rate 18.9% at GCS<4 vs. 77.27% GCS>4
- Survival to discharge OR 0.91 (0.85; 0.98)

EMS-centered measures:

- Median response time 8 (IQR 5-11) vs. 6 (IQR4-9)

**Naroo 2012^82^** Patient-centered measures:

- Survival to discharge rate 6.67%
- Response time association with survival not assessed

EMS-centered measures:

- Mean response time 7.7 minutes

**Navab 2019^83^** Patient-centered measures:

- Survival to discharge rate 4.1%
- Survival to discharge OR 0.74 (0.69; 0.80), p-value < 0.001

EMS-centered measures:

- Median response time 6.0 minutes

**Nichol 2016^84^** Patient-centered measures:

- Survival to discharge rate 17.7%
- Favorable neurologic status 15.9%
- Response time association with survival not assessed

EMS-centered measures:

- Mean response time 6.1±2.4 minutes

**Nielsen 2021^85^** Patient-centered measures:

- 30-days survival rate 93.8%
- ICU admission 18.1%
- Response time association with survival not assessed

EMS-centered measures:

- Mean response time: N/A

**O’Keefe 2011^86^** Patient-centered measures:

- Survival to discharge rate 2.6% vs. 15.6% if witnessed by paramedics
- Survival to discharge OR 1.24 (1.04; 1.48), p-value 0.02

EMS-centered measures:

- Mean response time N/A

**Park 2017^87^** Patient-centered measures:

- Survival to discharge rate 10.6%
- Favorable neurological outcome rate 6.5%
- Survival to discharge OR 0.89 (0.66-1.20) fast group vs. OR 0.76 (0.57-1.02) late group
- Favorable neurological outcome OR 1.12 (0.77-1.62) vs. OR 0.90 (0.62-1.30)

EMS-centered measures:

- Median response time 7(IQR 5-0) minutes

**Pell 2001^88^** Patient-centered measures:

- Survival to discharge rate 6.0%
- Response time association with survival not assessed

EMS-centered measures:

- Mean response time N/A

**Pons 2005^89^** Patient-centered measures:

- Survival to discharge rate 92.0%
- Survival to discharge OR 1.01 (0.98; 1.04)

EMS-centered measures:

- Median response time 5.8 (IQR4.3-7.7)

**Pons 2002^90^** Patient-centered measures:

- Survival to discharge rate 94.0% RT<8 min. vs. 95.9% RT>8 mins.
- Survival to discharge OR 0.81 (0.43; 1.52), p-value 0.51

EMS-centered measures:

- Mean response time 4.7±1.7 vs. 10.4±3.3 minutes

**Puolakka 2023^91^** Patient-centered measures:

- Survival to discharge rate 35.1%
- Response time association with survival not assessed

EMS-centered measures:

- Median response time 9 (7-11) minutes.

**QuocDo 2010^92^** Patient-centered measures:

- Survival to ROSC 20.0%
- Response time association with survival not assessed

EMS-centered measures:

- Mean response time 6.16 (ROSC vs. 6.57 minutes (non-ROSC)

**Rajan 2016^93^** Patient-centered measures:

- 30-days survival rate: 2.7% -22.6%
- Response time association with survival not assessed

EMS-centered measures:

- Median response time 7.0 minutes

**Renkiewicz^94^** Patient-centered measures:

- Survival to ROSC 40.6% (non-shockable) vs. 61.7% (shockable)
- Response time association with survival not assessed

EMS-centered measures:

- Mean response time 9.36±4.1

**Schinnerl 1990^95^** Patient-centered measures:

- Survival to discharge 4.44%
- Response time association with survival not assessed

EMS-centered measures:

- Mean response time 6.88±4.19

**Semensato^96^** Patient-centered measures:

- 30-days survival rate 3.9%
- Response time association with survival not assessed

EMS-centered measures:

- Median response time 13 (IQR 9-18) minutes

**Shah 2010^97^** Patient-centered measures:

- Survival to discharge rate 8.7%/8.9% between groups
- Response time association with survival not assessed

EMS-centered measures:

- Mean response time 7.48 vs. 7.46 minutes between groups

**Shepherd 2008^98^** Patient-centered measures:

- Survival to 24H discharge rate: 26.0%
- Response time association with survival not assessed

EMS-centered measures:

- Mean response time 48.62 minutes

**Sigursson 2000^99^** Patient-centered measures:

- Survival to discharge rate 26.0%
- Response time association with survival not assessed

EMS-centered measures:

- Mean response time 4.6 minutes

**Sipria 2016^100^** Patient-centered measures:

- Survival to discharge and CPC 1-2 rate 10.2%
- Survival to discharge OR 0.37 (0.21; 0.64)

EMS-centered measures:

- Median response time 6 minutes in survivors vs. 7 minutes in non-survivors

**Spaite 2008^101^** Patient-centered measures:

- Survival to discharge rate 5.9%
- Survival to discharge OR 0.43 (0.25; 0.73)

EMS-centered measures:

- Mean response 5.4 (5.2-5.5) minutes

**Stoeckl 2010^102^** Patient-centered measures:

- Survival to discharge rate 9.0%
- Response time association with survival not assessed

EMS-centered measures:

- Mean response time 8.31 minutes for survivors vs. 9.04 minutes in non-survivors

**Stromsoe 2015^103^** Patient-centered measures:

- 30-days survival rate 2.0% - 11.0%
- Response time association with survival not assessed

EMS-centered measures:

- Median response time 6.0 – 11.0 minutes

**Stromsoe 2011^104^** Patient-centered measures:

- 30-days survival rate 4.8% vs. 10.7%
- Response time association with survival not assessed

EMS-centered measures:

- Mean response time 11.0 vs. 15.0 minutes

**Sugiyama 2023^105^** Patient-centered measures:

- 30-days survival rate 3.7% (COVID) vs. 5.7% (non-COVID)
- 30-days survival OR 0.86 (0.75; 0.99)

EMS-centered measures:

- Median response time 8.7 (IQR 7.0-10.9) vs. 8.3 (IQR 6.7-10.5)

**Syvaoja 2018^106^** Patient-centered measures:

- Survival to discharge rate 22.0%
- Response time association with survival not assessed

EMS-centered measures:

- Median response time 8 (IQR 6.5-10.0)

**TerAvest 2019^107^** Patient-centered measures:

- Survival to discharge 13.73%
- Response time association with survival not assessed

EMS-centered measures:

- Mean response time 30 (13-109) minutes

**Thompson^108^** Patient-centered measures:

- Survival to discharge rate 10.0% (traumatic OHCA)
- Response time association with survival not assessed

EMS-centered measures:

- Mean response time 12 ± 13.5 minutes

**Trenkler 2012^109^** Patient-centered measures:

- 30-days survival 6.7%
- Response time association with survival not assessed

EMS-centered measures:

- Mean response time 8.6 minutes.

**Tsai 2017^110^** Patient-centered measures:

- Survival to discharge rate 11.7%
- Survival to discharge OR 0.83 (0.70; 0.98)

EMS-centered measures:

- Mean response time 7.51 ± 3.1 minutes

**Wai 2005^111^** Patient-centered measures:

- Survival to discharge rate 0.8%
- Response time association with survival not assessed

EMS-centered measures:

- Mean response time N/A

**Weinlich 2019^112^** Patient-centered measures:

- Survival to discharge rate 92.2%
- Response time association with survival not assessed

EMS-centered measures:

- Mean response time 9.0 (1-64)

**Wik 2003^113^** Patient-centered measures:

- Survival to discharge rate 22.1% (CPR first) vs. 14.6% (standard)
- Survival to discharge OR 1.41 (1.03; 1.94)

EMS-centered measures:

- Mean response time 12.0 (10.7-13.4) vs. 11.7 (10.7-12.7)

**Wissa 2021^114^** Patient-centered measures:

- Survival to discharge rate 35.2%
- Survival to discharge OR 0.98 (0.94; 1.03)

EMS-centered measures:

- Median response time 8 (IQR 6-11)


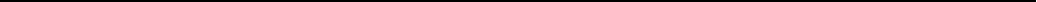


**Zheng 2023^115^** Patient-centered measures:

- 30-days survival rate 1.2%
- Response time association with survival not assessed

EMS-centered measures:

- Median response time 12 min (IQR 9 – 16).

Legend: CI: Confidence interval; COVID-19: Coronavirus disease 2019; CPC: Cerebral performance category; CPR: Cardiopulmonary resuscitation; EMS: Emergency medical services; HEMS: Helicopter emergency medical services; ICU: Intensive care unit; IQR: Interquartile range; mins.: minutes; N/A: not applicable; OHCA: Out-of-hospital cardiac arrest; OR: Odds ratio; ROSC: Return of spontaneous circulation; RT: Response time;

**Supplemental table 8. Outcomes and study periods**

| **Reference** | **Patient centered outcome 1** | **Patient centered**  **outcome 2** | **EMS centered measure 1** | **EMS centered measuree 2** | **Comments** | **Study period** |
| --- | --- | --- | --- | --- | --- | --- |
| **Abrams 2011^1^** | Survival to discharge | ROSC | Response time |  |  | 01.01.04-31.12.07 |
| **Afzali 2013^2^** | 30-day survival | ICU admission | Response time |  |  | 01.05.10-30.04.11 |
| **Ahmoudi 2022^3^** | Survival to discharge | ROSC | Response time | Collapse-to-CPR time |  | N/R |
| **Al-Dury 2020^4^** | 30-day survival |  | Response time | Collapse-to-CPR time |  | 01.01.08-31.12.16 |
| **Alqudah 2021^5^** | Survival to discharge | GOSE | Response time |  |  | 01.01.01-31.12.18 |
| **Alumran 2020^6^** | Survival to discharge |  | Response time |  |  | 01.01.17-31.10.18 |
| **Aziz 2020^7^** | Survival to discharge | CPC | Response time |  |  | 01.09.18-31.05.19 |
| **Azpiazu 2024^8^**  **Bagher 2017^9^** | Survival to discharge  90-day survival | NISS | Response time  Response time | On-scene time |  | 01.01.22-31.12.22  01.01.11-31.12.13 |
| **Baker 2008^10^** | Survival to discharge | CPC | Response time | 3 min. CPR first vs. defib. | Randomized controlled trial | 01.07.05-31.07.07 |
| **Berge 2005^11^** | 24H-survival | 1-year survival | Response time | Total transport time |  | 01.01.88-31.12.01 |
| **Biewener 2000^12^** | 24H-survival | 7D-survival | Response time |  |  | 01.01.93-31.12.94 |
| **Bjorkmann 2022^13^** | 30-day survival |  | Response time | On-scene time |  | 01.01.12-31.12.18 |
| **Bjornsson 2006^14^** | Survival to discharge |  | Response time |  |  | 01.01.99-31.12.02 |
| **Blackwell 2009^15^** | Survival to discharge |  | Response time |  |  | 01.01.04-31.12.04 |
| **Blaengsdottir 1994^16^** | Survival to discharge |  | Response time |  |  | 01.01.89-31.12.90 |
| **Blanchard 2012^17^** | Survival to discharge |  | Response time | Transport interval |  | 01.01.06-31.12.06 |
| **Bossers 2021^18^** | 30-day survival | GOS | Response time |  |  | 01.02.12-31.12.17 |
| **Brede 2020^19^** | 30-day survival | ROSC | Response time | CPR duration |  | 01.01.16-31.12.18 |
| **Brison 1992^20^** | Survival to discharge | Functional outcome* | Response time |  | *Instution/home/altered/previous | 01.03.86-31.07.89 |
| **Brown 2019^21^** | 30-day survival | LOS | Response time | On-scene time |  | 01.01.13-31.12.16 |
| **Bujak 2021^22^** | Survival to discharge | LOS | Response time | Defibrillation time |  | 01.01.18-31.12.18 |
| **Burger 2018^23^** | Survival to discharge | ROSC | Response time |  |  | 01.01.16-31.12.16 |
| **Byrne 2019^24^** | 30-day survival |  | Response time | On-scene time |  | 01.01.13-31.12.15 |
| **Cardoso 2014^25^** | 30-day survival | 24H-survival | Response time | On-scene time |  | 01.01.10-31.12.12 |
| **Chang 2018^26^** | Survival to discharge | CPC | Response time |  |  | 01.01.12-31.12.16 |
| **Chen 2015^27^** | Survival to discharge |  | Response time |  |  | 01.01.06-31.12.09 |
| **Chesters 2015^28^** | Survival to discharge | CPC | Call-to-arrival time* |  | *999 call to patient contact | 10.12.10-30.06.13 |
| **Claesson 2008^29^** | 30-day survival |  | Response time |  |  | 01.01.90-31.12.05 |
| **deGraaf 2019^30^** | 30-day survival |  | Response time | On-scene time |  | 01.01.12-31.12.16 |
| **Deasy 2012^31^** | Survival to discharge |  | Response time |  |  | 01.01.00-31.12.09 |
| **Dicker 2018^32^**  **Dinh 2023^33^** | 30-day survival  30-day survival |  | Response time  Response time |  |  | 01.10.13-30.09.15  01.01.19-31.12.20 |
| **Dyson 2013^34^** | Survival to discharge |  | Response time | Presenting rhythm |  | 01.10.99-31.12.11 |
| **Einarsson 1989^35^** | Survival to discharge |  | Response time |  |  | 01.01.82-31.12.86 |
| **Eisenburger 2001^36^** | Survival to discharge | 24H-survival | Response time |  |  | 01.01.91-31.01.98 |
| **Ong 2003^37^** | Survival to discharge |  | Response time | Collapse-to-call time |  | 01.10.01-30.04.02 |
| **Fake 2013^38^** | Survival to discharge | ROSC | Response time |  |  | 01.07.07-30.06.10 |
| **Finney 2023^39^** | ROSC |  | Response time | Presenting rhythm |  | 01.04.18-31.03.19 |
| **Fothergill 2021^40^** | 30-day survival | ROSC | Response time | On-scene time |  | 01.03.19-30.04.20 |
| **Fraga-Sastrias 2009^41^** | Survival to discharge | ROSC | Response time | Collapse-to-call time |  | 01.06.06-31.05.07 |
| **Gnesin 2021^42^** | 30-day survival | ROSC | Response time | Time to dispatch |  | 01.01.16-31.12.18 |
| **Goh 2018^43^** | Survival to discharge |  | Response time |  |  | 01.01.90-31.12.10 |
| **Goh 2013^44^** | Survival to discharge |  | Response time | Presenting rhythm |  | 01.10.01-30.09.04 |
| **Gregers 2021^45^** | 30-day survival | ROSC | Response time |  |  | 01.01.16-31.12.19 |
| **Grmec 2009^46^** | Survival to discharge | ROSC | Response time |  |  | 01.02.98-31.01.07 |
| **Gunaga 2020^47^** | Survival to discharge | ICU admission | Response time | Total prehospital time |  | 01.01.17-31.03.17 |
| **Han 2022^48^** | Survival to discharge | Neurological outcome* | Response time | Total transport time | *Unspecified | 01.01.17-31.12.18 |
| **Hayes 2010^49^** | Survival to discharge |  | Response time | Arrest to BLS time |  | 01.01.07-31.12.07 |
| **Henry 2013^50^** | Survival to discharge | ROSC | Response time |  |  | 01.09.07-31.08.08 |
| **Herlitz 2006^51^** | 30-day survival | ROSC | Call-to-arrival time* |  | *999 call to patient contact | 01.01.90-31.12.05 |
| **Herlitz 2008^52^** | 30-day survival | ROSC | Response time | Call-to arrival time* | *999 call to patient contact | 01.01.90-31.12.05 |
| **Hillis 1993^53^** | Survival to discharge | Sickness Impact Profile | Response time |  |  | 01.01.87-31.10.89 |
| **Holmen 2020^54^** | 30-day survival |  | Response time |  |  | 01.01.08-31.12.17 |
| **Huabbangyang 2022^55^** | ROSC |  | Response time |  |  | 01.05.19-30.04.20 |
| **Hubert 2016^56^** | Survival to discharge | CPC | Response time |  |  | 01.07.11-30.11.14 |
| **Jennings 2006^57^** | Survival to discharge |  | Response time |  |  | 01.01.22-31.12.23 |
| **Jeong 2017^58^** | Survival to discharge |  | Response time |  |  | 01.01.12-31.12.16 |
| **Kennedy 2023^59^** | Survival to discharge |  | Response time |  |  | 01.03.20-31.12.21 |
| **Kentsch 2000^60^** | Survival to discharge | Neurological outcome* | Response time |  | *Unspecified | 01.01.84-31.12.88 and 01.01.91-31.12.97 |
| **Kitano 2022^61^** | 30-day survival | ROSC | Response time | On-scene time |  | 01.01.14-31.12.19 |
| **Klosiewicz 2017^62^** | ROSC |  | Response time | Call-to arrival time* | *999 call to patient contact | 01.01.15-31.12.15 |
| **Lee 2019^63^** | Survival to discharge | Favorable neurological outcome | Response time |  |  | 01.10.15-31.12.16 |
| **Lee 2013^64^** | Survival to discharge | CPC | Response time | Total transport time |  | 01.01.08-31.05.11 |
| **Leung 2001^65^** | Survival to discharge |  | Response time |  |  | 15.03.99-15.10.99 |
| **Lim 2020^66^** | Survival to discharge | ROSC | Response time | On-scene time |  | 01.01.11-31.12.16 |
| **Lin 2014^67^** | Survival to discharge |  | Response time |  |  | 01.05.13-31.05.13 |
| **Little 2020^68^** | Survival to discharge | ICU admission | Response time | Call-to-door time |  | 01.03.19-30.04.19 and 01.03.20-30.04.20 |
| **Liu 2023^69^** | Survival to discharge | ROSC | Response time | Total prehospital time |  | 01.01.18-31.12.21 |
| **Lyon 2013^70^** | Survival to discharge |  | Response time | Call-to-arrival time* | *999 call to patient contact | 01.09.10-01.09.11 |
| **Margey 2011^71^** | Survival to discharge |  | Response time | On-scene time |  | 01.01.03-31.12.08 |
| **Mathiesen 2018^72^** | Survival to discharge |  | Response time | Physician presence |  | 01.01.06-31.12.15 |
| **Mayer 1979^73^** | Survival to discharge |  | Response time |  |  | 01.01.77-31.08.78 |
| **Meyer 2001^74^**  **Mikiewicz 2023^75^** | Survival to discharge  Survival to discharge |  | Response time  Call-to-arrival time |  |  | 01.01.97-31.12.97  01.01.01-  31.12.21 |
| **Mikkelsen 2017^76^** | 30-day survival | 90-day survival | Response time |  |  | 01.05.06-31.12.14 |
| **Mills 2023^77^** | 30-day survival |  | Response time |  |  | 01.01.14-31.12.18 |
| **Mills 2019^78^** | 30-day survival | 24H-survival | Response time | Total prehospital time |  | 04.04.06-01.12.12 |
| **Mogensen 2015^79^** | Survival to discharge |  | Response time |  |  | 01.01.04-31.12.07 |
| **Morrisey 1996^80^** | 30-day survival |  | Response time | On-scene time |  | 01.01.91-31.12.91 |
| **Nadolny 2021^81^** | Survival to discharge |  | Response time |  |  | 01.01.18-31.12.18 |
| **Naroo 2012^82^** | 30-day survival |  | Response time |  |  | 01.04.11-30.07.11 |
| **Navab 2019^83^** | Survival to discharge |  | Response time |  |  | 01.04.16-28.02.17 |
| **Nichol 2016^84^** | Survival to discharge | Favorable neurological outcome | Response time | Call-to-arrival time* |  | 01.01.99-31.12.12 |
| **Nielsen 2022^85^** | 30-day survival | 24H-survival | Response time |  |  | 01.10.14-30.09.18 |
| **O'Keefe 2011^86^** | Survival to discharge | Favorable neurological outcome | Response time | Presenting rhythm |  | 01.01.96-31.12.00 |
| **Park 2017^87^** | Survival to discharge | CPC | Response time |  |  | 01.01.12-31.12.14 |
| **Pell 2001^88^** | Survival to discharge |  | Response time |  |  | 01.05.91-01.03.98 |
| **Pons 2005^89^** | Survival to discharge |  | Response time | Total prehospital time |  | 01.01.98-31.12.98 |
| **Pons 2002^90^** | Survival to discharge |  | Response time | Call-to arrival time* |  | 01.01.94-31.12.98 |
| **Puolakka 2023^91^** | Survival to discharge | CPC | Response time |  |  | 01.01.16-31.12.21 |
| **Do 2010^92^** | ROSC |  | Response time |  |  | 01.01.02-31.12.08 |
| **Rajan 2016^93^** | 30-day survival |  | Response time |  |  | 01.01.05-31.12.11 |
| **Renkiewicz 2014^94^** | ROSC |  | Response time | Presenting rhythm |  | 01.01.12-30.06.12 |
| **Schinnerl 1990^95^** | Survival to discharge | Neurological outcome* | Response time |  | *Unspecified | 18.11.85-17.11.88 |
| **Semensato 2011^96^** | 30-day survival | CPC | Response time | Arrest to BLS time |  | 26.01.08-21.10.08 |
| **Shah 2010^97^** | Survival to discharge |  | Response time |  |  | 01.04.03-31.03.07 |
| **Shepherd 2008^98^** | Death* |  | Response time | On-scene time | *Unspecified | 01.01.04-30.11.06 |
| **Sigursson 2000^99^** | Survival to discharge |  | Response time |  |  | 01.01.91-31.12.96 |
| **Sipria 2016^100^** | Survival to discharge | CPC | Response time | Resuscitation time |  | 01.01.99-31.12.13 |
| **Spaite 2008^101^** | Survival to discharge |  | Response time | Total prehospital time |  | 01.10.04-31.12.06 |
| **Stoeckl 2010^102^** | Survival to discharge | Neurological outcome* | Response time | Dispatch interval | *Unspecified | 01.02.09-31.01.10 |
| **Stromsoe 2011^103^** | 30-day survival |  | Population density |  |  | 01.01.08-31.12.09 |
| **Stromsoe 2015^104^** | 30-day survival | CPC | Response time | Collapse-to-call time |  | 01.01.92-31.12.11 |
| **Sugiyama 2023^105^** | 30-day survival | Favorable neurological outcome | Response time | On-scene time |  | 01.03.20-31.09.22 |
| **Syvaoja 2018^106^** | Survival to discharge | ROSC | Response time |  |  | 01.01.97-31.12.13 |
| **TerAvest 2019^107^** | Survival to discharge | ROSC | Call-to-arrival time* |  | *999 call to patient contact | 01.01.13-01.01.18 |
| **Thompson 2017^108^** | Survival to discharge | GCS | Transport interval | Call-to-arrival time* | *999 call to patient contact | 01.04.12-30.09.12 |
| **Trenkler 2012^109^** | 30-day survival | ROSC | Response time |  |  | 01.01.11-31.12.11 |
| **Tsai 2017^110^** | Survival to discharge |  | Response time | On-scene time |  | 01.01.12-31.12.15 |
| **Wai 2005^111^** | Survival to discharge |  | Collapse-to-hospital time | Collapse-to-defibrillation time |  | 01.07.02-31.12.02 |
| **Weinlich 2019^112^** | Survival to discharge | GOS | Response time | On-scene time |  | 01.01.09-31.12.13 |
| **Wik 2003^113^** | Survival to discharge | CPC | Response time |  | Randomized controlled trial | 01.06.98-31.05.01 |
| **Wissa 2021^114^**  **Zheng 2023^115^** | Survival to discharge  Survival to discharge |  | Response time  Response time |  |  | 01.01.15-31.12.19  01.08.19-  31.12.20 |

**Legend:** BLS: Basic life support; GCS: Glasgow coma scale; CPC: Cerebral performance category; CPR: Cardiopulmonary resuscitation; Defib.: defibrillation; EMS: Emergency medical services; GOS: Glasgow outcome scale; GOSE: Extended Glasgow outcome scale; ICU: Intensive care unit; LOS: Length of stay; NISS: New Injury Severity Scale; N/R: Not registered; ROSC: Return of spontaneous circulation; 7D: 7-days; 24H: 24-hours

**Supplemental Table 9. Survival rates related to study characteristics from the included literature**

Study characteristics Studies, n Survival rate, %, median (inter-quartile range)

_______________________________________________________________________________________

**Publication year**

1970 – 1979 1 30.2 (30.2; 30.2)

1980 – 1989 1 17.0 (17.0; 17.0)

1990 – 1999 5 4.4. (3.3; 17.0)

2000 – 2009 20 8.1 (1.8; 23.1)

2010 – 2019 42 10.1 (6.7; 15.5)

2020 – 2024 30 16.6 (11.1; 69.0)

**Geographical location**

Europe 47 13.0 (8,5; 29,5)

North America 13 31.0 (11.1; 93,6)

South America 3 3.9 (2.0; 44.0)

Middle East 4 5.8 (4,7; 10.4)

Asia 20 7.2 (1.8; 11.0)

Australia/Oceania 12 10.9 (6,6; 21.2)

**Organization**

Emergency medical services 35 9.5 (5.4; 14.0)

Ground EMS only 39 11.2 (5.2; 20.4)

Helicopter EMS only 6 86.0 (31.2; 91.1)

Mobile emergency care unit only 6 21.5 (7.6; 76.9)

Paramedic-manned EMS only 2 42.2 (23.3; 61.6)

Mixed** 11 18.0 (11.6; 29.0)

**Setting**

Metropolitan 14 4.7 (1.5; 9.8)

Rural 6 10.5 (7.0; 73.4)

Suburban 1 98.6 (98.6; 98.6)

Urban 15 13.6 (9.4; 18.5)

Mixed 63 11.7 (6.4; 30.9)

**Diagnoses investigated in study**

Drowning 2 8.5 (8.3; 8.8)

Miscellaneous* 7 91.4 (75.9; 93.9)

Out-of-hospital cardiac arrest 79 10.2 (5.0; 17.9)

Trauma 7 92.2 (86.0; 95.6)

Traumatic out-of-hospital cardiac arrest 4 7.55 (4.5; 10.9)

**Overall** 99 11.5 (5.2; 25.8)

Legend: EMS: Emergency medical service; OHCA; *Unspecified in the included study; **E.g., Ground EMS/Helicopter EMS

**Supplemental Table 10. Narrative interpretation of the included literature**

**Author/year Narrative interpretation**

**Abrams 2011^1^** Objective: Survival rate for OHCA of cardiac etiology and survival predictor variables. Methods: Retrospective analysis of all arrests of presumed cardiac etiology. Results: The survival-at-hospital discharge rate was 11%. Response time, public location, witnessed, and age are significant but less sizable direct predictors of survival. A second equation shows that these four variables make an additional indirect contribution to survival by affecting the probability of joint presence of VF/VT and ROSC; bystander CPR also makes such an indirect contribution.

**Afzali 2013^2^** Objective: To describe activity and possible beneficial effect of a physician-staffed helicopter in a one-year trial period in Eastern Denmark. Methods: Prospective observational study of all missions. Results: 574 missions, 609 patient contacts. Median LOS was four days, 30-day mortality was 6.1% and 86 patients were transferred to intensive care. Conclusion: Two missions per day the first year, mainly in relation to trauma and cardiac patients needing specialized treatment.

**Ahmoudi 2022^3^** Objective: To identify the association of average time interval from patient collapsed to first CPR by EMS and it is survival to hospital discharge. Methods: Cross-sectional approach based on a quantitative prospective data. Results: ROSC was observed in 17%. Interval time of the patient arrest to EMS CPR for those who did not survive at 15.11 mins, which is significantly higher than survivors at 7.27 minutes. Conclusion: Patients were more likely to survive if basic life support is started < 7 mins from collapse.

**Al-Dury 2020^4^** Objectives: To investigate the relative importance of 16 well recognized factors in OHCA. Methods: Study of the relative importance of 16 factors assessed during the pre-hospital phase of OHCA. Results: 45 067 cases of OHCA; top five factors to predict survival in order of importance were initial rhythm, age, early CPR, time from EMS dispatch until EMS arrival, and place of cardiac arrest. Conclusion: Most important predictor of survival in OHCA is initial rhythm, followed by age, time to start of CPR, EMS response time and place of OHCA.

**Alqudah 2021^5^** Aim: The impact of temporal changes in the epidemiology and management of traumatic OHCA survival outcomes. Methods: A retrospective observational study of traumatic OHCA; logistic regression to assess trends in survival outcomes over the study period. Results: 5 631 patients; the frequency of EMS trauma-specific interventions increased over the study period. No temporal changes in survival. Conclusion: Rates of survival following traumatic OHCA did not change overtime in our region.

**Alumran 2020^6^** Objective: To investigate the role of response time, whether more or less than 8 minutes, on the survival in OHCA. Methods: Retrospective observational study. Results: 108 OHCA cases; bivariate analysis showed no significant association between response time and patient outcomes; however, the odds of having a negative outcome (death) if the response time is more than 8 minutes is double the odds of dying if the response time is less than 8 minutes. Conclusion: Ambulance response time to OHCA does not significantly influence the patient survival rate.

**Aziz 2020^7^** Objective: To investigate OHCA outcomes in relation to PHC services. Method: Prospective study conducted on OHCA cases. Results: 82 OHCA cases. Survival to admission was 12.2%, survival rate to discharge was 1.2%, mean ambulance response time was 14.91 min. Conclusion: Improvement in ambulance response time, public availability of automated external defibrillator, and public awareness of early cardiac arrest and cardiopulmonary resuscitation are required to increase the survivability of OHCA in developing countries.

**Azpiazu 2024^8^** Out-of-hospital cardiac arrest is a serious public health problem worldwide. The annual incidence is estimated at around 400 000 cases in Europe and the United States, and survival rates scarcely reach 10%. However, there is considerable variation between countries and even between regions that share a similar health care system within a single country. Information recorded by the Out-of-Hospital Spanish Cardiac Arrest Registry (OHSCAR) provides information on care provided by emergency ambulance services, final health outcomes after cardiac arrest cases (including variations), the possibility of organ donation, and the impact of the COVID-19 pandemic. This paper presents the OHSCAR report for Spanish emergency services for the year 2022

**Bagher 2017^9^** Objective: To analyze if pre-hospital rescue times were associated with mortality. Methods: Retrospective observational study. Results: 378 trauma patients; 89% received hospital care within 60 min; 51% had a response time of 8 mins. Conclusions: Pre-hospital rescue times had less impact on mortality than injury severity, age, and penetrating trauma.

**Baker 2008^10^** Objective: In VF OHCA, 3 min of CPR before defibrillation was more effective than immediate defibrillation. Methods: Randomized controlled study. Results: For all response times, no differences were observed between defibrillation group and CPR first group in survival to hospital discharge. Conclusion: In VF OHCA, we found no evidence to support the use of 3 min of CPR before the first defibrillation over the accepted practice of immediate defibrillation.

**Berge 2005^11^** Objective: To describe neonatal transport by HEMS in central Norway and report the outcome. Methods: Retrospective analysis. Results: 252 neonates were transported, indicating a prevalence of 0.90 per 100 newborns. Median response time was 42 min. Conclusion: HEMS provides rapid medical assistance in a wide spectrum of neonatal problems, but more attention should be paid to proper ventilation and prevention of hypothermia and hypoglycemia.

**Biewener 2000^12^** Objective: To examine patients who died in sequel of an accident. Methods: Data were assessed from autopsy protocol and the protocol of the physician who treated on scene Results: 122 cases were included. The mean response time was 8,1±5,9 min, the mean distance between EMS bases und incident location 5,9±5,7 km. HEMS was performed only in 8,7% of all cases. 82 patients reached the emergency room alive. Mean survival time of all 122 patients was 146±30,4 h. Only 26% of all patients were transported directly to a level I trauma center. Conclusion: Primary transport of the severely injured patient to a level I trauma center by helicopter was performed only rarely.

**Bjorkman 2020^13^** Objective: To investigate the effect of prehospital time-intervals on 30-day mortality on trauma patients. Methods: Retrospective observational study on all trauma patients encountered by HEMS. Results: 4 803 patients; 30-day mortality was 12.1% (582/4 803). No association between time intervals and 30-day mortality. Conclusion: No significant association between different timespans and mortality following severe trauma in general.

**Bjornsson 2006^14^** Objectives: Effects on EMS changes and bystander CPR on OHCA survival Methods: Retrospective observational study on OHCA characteristics. Results: 319 OHCA cases; average response time was 6,1 min. 19%) survived to discharge with 39 being alive at 12 months. Conclusion: In 54% of the cases CPR was performed by bystanders. Response time needs to be shortened and CPR training increased.

**Blackwell 2006^15^** Objective. Survival independent of response times >/< 11 minutes. Methods. Retrospective case– control retrospective study. Results. 373 study patients >11 min response time, 373 controls. Survival to hospital discharge was 80% (76% to 84%) for study patients vs. 82%. Conclusions: Compared with patients who wait 10:59 minutes or less for ALS response, Priority 1 patients who wait longer than 10:59 minutes could experience between a 6% increase and a 4% decrease in mortality.

**Blaengsdottir^16^** Objective: Evaluate ACLS and bystander influence on survival after OHCA. Methods: Retrospective observational study. Results: 308 OHCA cases; mean response time was 4.6 min. 17% survival to discharge. VF/VT most common Bystanders were present in 68%. Conclusions: When sudden cardiorespiratory arrest is witnessed the probability of survival is multiplied.

**Blanchard 2012^17^** Objective. To explore whether an 8-minute EMS response time was associated with mortality. Methods. Retrospective observational study. Results. 7 760 patients; 24% ≥8 minutes. Response time ≥8 minutes, 7.1% died, compared with 6.4% for patients with a response time <8 mins. Conclusion: Shorter response time associated with survival.

**Bossers 2021^18^** Objective: describe the prehospital epidemiology, characteristics and outcome of (suspected) severe TBI in the Netherlands. Methods: The BRAIN-PROTECT study is a prospective observational study on prehospital management of patients with severe TBI in the Netherlands. Results: 2 589 patients; median time from HEMS dispatch to hospital arrival was 54 minutes. The overall 30-day mortality was 39.0% (95% CI: 36.8; 41.2). Conclusion: This article summarizes the prehospital epidemiology, characteristics and outcome of severe TBI in the Netherlands.

**Brede 2020^19^** Objective: To assess the need for REBOA in OHCA. Methods: Retrospective observational study. Results: 2 241 patients; very few eligible for REBOA. Conclusion: 9% of ambulance treated OHCA, in Norway is potentially eligible for pre-hospital REBOA.

**Brison 1992^20^** Objectives: OHCA characteristics; effect on survival of defibrillation; identify survival predictors. Methods: Retrospective observational study; population-based before-and-after clinical trial. Results: 1 510 patients; The average ambulance response time for witnessed cases was 7.8 minutes. The overall survival rate was 2.5%. The survival rates before and after defibrillators were introduced were similar, and the general functional outcome of the survivors did not differ significantly between the two phases. Factors predicting survival included patient's age, ambulance response time and whether CPR was started before the ambulance arrived. Conclusions: Defibrillation did not affect survival.

**Brown 2019^21^** Objective: To determine the association between prehospital time and outcomes in adult major trauma. Methods: Retrospective cohort study of major trauma patients. Results: 1 625 patients; no significant association between prehospital time of one hour and 30-day mortality was found (adjusted odds ratio 1.10, 95% confidence interval (CI) 0.71–1.69). Conclusion: Longer prehospital times were not associated with an increased likelihood of 30-day mortality in major trauma.

**Bujak 2021^22^** Objective: To evaluate the epidemiology of OHCA. Methods: Retrospective observational study. Results: 1 392 patients. 66.8% of OHCA witnessed by bystanders and 20.4% by EMS, 30.7% of all patients were transported to the hospital, and 9.2% survived to hospital discharge. Epinephrine administration, unwitnessed OHCA, longer response time, older age, and initial non-shockable rhythm were independently associated with lower survival to discharge. Conclusions: The prognosis of OHCA patients in Poland is poor.

**Burger 2018^23^** Objective: To study the effect of ambulance response time on survival in OHCA. Methods: Retrospective observational study. Results: 10 853 patients; survival to discharge was significantly affected by the ambulance response time Conclusion: Rapid ambulance response is associated with a higher rate of survival from OHCA with good neurological outcome.

**Byrne 2019^24^** Objectives: To measure the association between EMS response times and MVC mortality at the population level across American counties. Methods: Retrospective observational study. Results: 2 214 480 ambulance responses to MVC; median response time was 9 minutes. Longer response times were significantly associated with higher rates of MVC mortality (≥12 vs <7 minutes; mortality rate ratio, 1.46; 95% CI, 1.32-1.61) after adjusting for measures of rurality, on-scene and transport times, access to trauma resources, and traffic safety laws. Conclusions: Longer EMS response times were associated with higher rates of MVC mortality.

**Cardoso^25^** 2014 Objective: To analyze the profile of patients served by the air medical rescue. Methods: We conducted a prospective, descriptive study. Results: of the 220 cases evaluated. The average response time was 10 ± 4 minutes and the averaged total pre-hospital time was 42 ± 11 minutes. Conclusion: studies of air medical rescue in Brazil are required due to the investments made in the pre-hospital care in a country without an organized trauma system.

**Chang 2018^26^** Objective: To investigate the effect of bystander CPR with dispatcher assistance on neurological outcomes based on the response time interval (RTI) of the pre-hospital emergency medical service (EMS). Methods: This retrospective registry study was conducted on pediatric patients (<19 years old) with OHCA assessed by EMS 2012-2016. Results:  The faster EMS RTI group (<5 min) had better neurological recovery than the later EMS RTI group (≥5 min) (AOR: 1.87 [1.04-3.29]). The AORs for good neurological recovery following BCPR with DA based on the EMS RTI were 2.52 (0.91-6.97) in the faster EMS RTI group and 2.17 (1.13-4.19) in the later EMS RTI.

**Chen 2015^27^** Objective: To determine whether EMS-related ambulance team process measures correlate with patient survival in OHCA. Methods: Retrospective observational study; EMS response time ≤4 min and prehospital Results: 3 856 OHCA patients distributed across forty-three EMS ambulance teams. Survival to discharge 5%. The two EMS team process measures were positively associated with an improvement in survival at the patient level after case-mix adjustment. However, they were not associated with improvement in the risk-adjusted survival rate. Conclusions: The EMS team-level process measures proposed by international institutes may not predict the risk-adjusted survival rate.

**Chesters 2015^28^** Objective: To report the outcomes of medical cardiac arrests according to the Utstein style. Methods: Retrospective database analysis and hospital follow-up of all non-traumatic cardiac arrests attended by either service over a 31-month period. Results: 193 patients achieved return of spontaneous circulation, sustained at handover to the hospital team. Of 140 follow-up patients, the overall survival rate was 50.7%, 86% of whom had a Cerebral Performance Category of 1 or 2. The overall survival-to-discharge rate for all patients attended was 11.7%. Conclusion: 31 months of data that pertain to medical cardiac arrest cases attended by our services and demonstrated a comparable survival rate to discharge with good neurological outcome.

**Claesson 2008^29^** Objective: To describe the characteristics and outcome among patients OHCA caused by drowning as compared with OHCA caused by a cardiac etiology. Methods:  Patients included in the Swedish OHCA Registry between 1990 and 2005. Results: 255 patients with OCHA due to drowning differed from patients with OHCA with a cardiac etiology (n=7494) as they were younger, less frequently suffered a witnessed OHCA, more frequently received bystander CPR and less frequently were found in a shockable rhythm. Conclusion: OHCA 0.9% were caused by drowning. They had a similar survival rate to 1 month as compared with OHCA outside home with a cardiac etiology.

**Deasy 2012^30^** Objectives: To describe the characteristics and profile of adult traumatic OHCA. Methods: Retrospective observational study. Results: 2 187 traumatic OHCA. EMS attempted resuscitation in 545 (24.9%) patients of whom 84 (15.4%) achieved ROSC and were transported, and 27 (5.1%) survived to hospital discharge. Conclusion: In paramedic delivered EMS attempted resuscitation was not always futile in traumatic OHCA with a survival of 5.1%.

**de Graaf 2019^31^** Objectives: To determine the time of resuscitation on scene ('time on scene') and survival in patients transported with ongoing CPR in the Netherlands. Methods: Retrospective observational study. Data on OHCA patients (>18 years) without ROSC on scene, where resuscitation was started. Results:  2437 of 5871 OHCA patients where resuscitation was started, did not achieve ROSC on scene. Of these, 655 patients were transported with ongoing CPR and 606 (93%) had complete rhythm data. Twenty-nine patients (4%) were alive at 30 days. In a multivariable model time on scene (OR 0.94; 95%CI 0.89-0.99) was independently associated with 30-day survival. Conclusion: In OHCA patients transported with ongoing CPR, the survival rate significantly declines when time on scene increases.

**Dicker 2018^32^** Objective: To describe the incidence and outcomes from OHCA. Methods: A retrospective observational study. Results: 3 862 cases; median response time was 9 min. Bystander CPR was administered in 62% of cases and 8% were defibrillated prior to EMS arrival. ROSC in 30% of events and 15% survived to 30 days. Conclusion: Findings provide important baseline data to monitor temporal trends, investigate the impact of changes in the management of OHCA and demonstrate that there are opportunities for improvement across the system of care.

**Dinh 2023**^33^ Introduction: Prehospital trauma systems are designed to ensure optimal survival from critical injuries. Objectives: We sought to evaluate whether prehospital time and location were associated with 30-day mortality using a trauma transport protocol. Median prehospital transport times were longer in non-metropolitan road transports compared to metropolitan transports. There was no significant difference in 30-day mortality between the two groups (1.24% vs 1.65%, p = 0.13.Discussion: In the context of an inclusive trauma system and an established prehospital major trauma protocol, increasing prehospital transport times and scene location were not associated with increased mortality.

**Dyson 2013^34^** Objective: To describe cardiac arrest due to drowning. Methods: Retrospective observational study. Results: 336 patients; resuscitation was attempted on 154 (46%) patients. 27% survived to hospital arrival and 8% survived to hospital discharge. Increased EMS response time (AOR 0.73, 95% CI: 0.54–0.98) and salt water drowning (AOR 0.69, 95% CI: 0.01–0.84) were found to negatively predict survival. Conclusions: Patients were more likely to survive if they did not drown in salt water, had a quick EMS response and they were found in a shockable rhythm.

**Einarsson 1989^35^** Objective: To assess OHCA treatment and outcome. Methods: retrospective observational study. Results: 138 OHCA patients. 17% were discharged home, all but one without mental impairment. The mean ambulance response time was 5 min. Bystanders initiated CPR in 29 % which significantly improved the outcome. In witnessed arrests, 19 of 36 patients (53 %) with bystander-initiated CPR were discharged compared to 5 of 62 patients (8 %) where CPR awaited the arrival of the ambulance team. Conclusions: In small urban areas, an advanced and efficient prehospital care can be organized as an extension of the emergency department’s role.

**Eisenburger^36^** Objective: To evaluate survival after OHCA in a rural alpine area. Methods: Retrospective observational study with prospective data collection. Results: 338 patients resuscitation was attempted. VF in 118 patients, 4% defibrillated. Response time 8 min to arrival of first tier and 16 min to defibrillation. ROSC in 46%, 1 year survival was 1 (8%) versus 20 (19%). Conclusion: In OHCA in alpine areas, response intervals and survival rate are not as poor as might be expected and are similar to metropolitan areas.

**Ong^37^**  Objectives. To describe the epidemiology OHCA, EMS response, and to identify possible areas for improvement. Methods. Prospective observational study, Utstein style. Results: 548 patients; mean EMS response time was 10.2 (4.3) minutes. Mean (SD) time from call to defibrillation was 16.7 (7.2) minutes. 17.9 % had ROSC, 8.5% survived to hospital admission, and 2.0% survived to discharge. Conclusion: Baseline for incremental introduction of prehospital ACLS.

**Fake 2013^38^** Objectives: To examine the influence of physical location on survival from out-of-hospital cardiac arrest (OHCA). Methods: Retrospective observational study. Comparison of OHCA characteristics and outcomes between public and residential locations and socioeconomic status. Results: 413 met the inclusion criteria. Survival from OHCA in public locations was approximately twice that for residential OHCA (19.8% vs 10.7%, p=0.021. No association between socioeconomic status and witnessing of the event, bystander cardiopulmonary resuscitation, the initial presenting rhythm, and ambulance response time. Conclusion: Ambulance response times and survival were not correlated with socioeconomic status.

**Finney 2023^39^** Objective: To quantitatively review and compare the OHCA response, treatment and pre-hospital outcomes Methods: Retrospective observational study. Results: 1 614 cases were compared. Bystander public-access defibrillator use was higher in rural areas in comparison to urban areas (20/319 (6.3%) vs 47/1295 (3.6%); p = 0.03). The mean ambulance response time was slower in rural areas. Overall ROSC rates at hospital were similar between the groups. Conclusion: This report showed differences in OHCA response and outcomes between rural and urban settings.

**Fothergill 2021^40^** Objective: To describe the incidence, characteristics and outcomes from OHCA in London during the first wave of the pandemic. Methods: Retrospective observational study. Results: 3 122 patients; 81% increase in OHCAs during the pandemic, and a strong correlation between daily number of COVID-19 cases and OHCA incidents (r=0.828, p<0.001). Survival at 30 days post-arrest was poorer during the pandemic (4.4% vs 10.6%, p<0.001). Conclusions: COVID-19 pandemic gave a dramatic rise in the incidence of OHCA, accompanied by a significant reduction in survival.

**Fraga-Sastrias^41^** Objective: To assess out-of-hospital cardiac arrest in a Mexican city. Methods: Prospective, cohort. Results: 148 OHCA cases. The collapse-assessment interval was 22.5 ±19:1 minutes, the mean value for the ambulance response times was 13:6 ±10:4 minutes. Chest compressions and airway control showed an OR of 8 and 12 respectively for ROSC. Conclusions: The poor survival rate in this study emphasizes the need to improve efforts in provider training and public education.

**Gnesin 2021^42^** Objectives: to investigate the effect of time to first dispatch on 30-day survival among patients with OHCA. Methods: all OHCA unwitnessed by EMS. Results: 3 548 patients. 94.1% received the highest priority response (median time to dispatch 0.84 min, 25th-75th percentile 0.58-1.24 min). Patients with time to dispatch within one minute compared to three or more minutes were more likely to receive bystander cardiopulmonary resuscitation (77.3 vs 54.2%), bystander defibrillation (11.5 vs 6.5%) and defibrillation by emergency medical services (24.1 vs 7.5%) and were 2.6-fold more likely to survive 30 days after the OHCA (P = 0.004). Conclusion: Rapid time to dispatch was significantly associated with a higher probability of 30-day survival following OHCA.

**Goh 2018^43^** Objectives: To examine the associations of local availability of types of medical facilities with SCA incidence, response times, and survival. Methods:  a population-based repository of data from adult cardiac arrest patients and population-based controls. Adult patients at SCA risk experiencing cardiac arrest (n = 446) were matched with controls (n = 208) without a history of heart disease. Results**:**More pharmacies in the home census tract was unexpectedly associated with higher odds of SCA (OR: 1.28, 95% CI: 1.03, 1.59), and similar associations were observed for other medical facility types. Conclusions: no protective association between medical facilities in the home census tract and SCA risk, or between major medical centers in the event census tract and survival.

**Goh 2013^44^** Objective: To study how the effect of the location of patient collapses in OHCA, Methods: A retrospective cohort study Results: 2 375 OHCA. Outcomes for OHCA in residential areas were poorer than in non-residential areas. Multivariate logistic regression analysis showed that location alone had no independent effect on. Instead, underlying factors such as bystander CPR and initial shockable rhythms gave rise to better outcomes. Conclusion: Efforts to improve survival from OHCA in residential areas should include increasing CPR by family members, and reducing ambulance response times.

**Gregers 2021^45^** Objectives: To evaluate the association between urbanization, bystander interventions, and 30-day survival from OHCAs. Methods: OHCA patients were divided in rural, suburban, and urban areas. Results: 21 385 OHCAs were included, of which 40% occurred in rural areas, 33% occurred in suburban areas, and 27% occurred in urban areas. 30-day survival was higher in suburban and urban areas compared with rural areas. Conclusions: degree of urbanization was associated with lower rates of bystander defibrillation and 30-day survival.

**Grmec 2009^46^** Objective: To compare the characteristics and outcome between patients suffering from OHCA and drowning victims in cardiac arrest by analysis of variables based on the USFD. Methods: Retrospective observational study. Results: 788 patients; 528 OHCA 67% and 4% drowning. ROSC in 65% vs. 57%; p = 0.33, discharge from hospital 44% vs. 22%; p = 0.01. Drowning had more bystander CPR, shorter call-arrival interval. Conclusion: Drowning due to cardiac arrest patients had a better survival and ambulance response time.

**Gunaga 2020^47^** Objective: To identify any quantitative differences in care between private and municipal EMS systems related to response times, intensity of services and patient outcomes. Methods: retrospective chart review. Results: 769 patients, 483 in the private EMS, 286 in the municipal EMS cohort. Conclusion: The current operating structure of providing EMS to our communities is based around the premise that there are no significant quality and safety differences between municipal and private EMS systems.

**Han 2022^48^** Objective: To investigate the relationship between the arrival time of last-arrival ambulance and the neurological outcome at discharge in OHCA. Methods: Retrospective observational study, comparing the early arrival team (response time of last-arrival ambulance < 14 minutes) and the late arrival team (response time of last-arrival ambulance > 14 minutes) with propensity score matching. Results 3 289 patients, optimal cut off was 14 minutes. 14-minute or longer group showed a significant association with poor neurological outcomes at discharge compared to less than 14 minutes [OR 2.87, 95% confidence interval 1.65-5.11, P value < 0.001]. Conclusion: Response time of last-arrival team 14-minute or longer was associated with poor neurological outcomes upon discharge in OHCA.

**Hayes 2010^49^** Objectives: to characterize the timelines and outcomes in OHCA. Methods Audit of consecutive non-traumatic OHCA victims. Results Of 74 OHCA victims, 69% male, aged 62 ± 19 years, the index event occurred at home in 86% of cases. Time from emergency call to ambulance arrival was 9 ± 4 minutes and from onset of OHCA to initiation of BLS measures was 13 ± 11 minutes. By-stander BLS was performed in 20% of cases. Time from OHCA to arrival in the ED was 35 ± 14 minutes. 15% of patients survived to ICU admission and 3% survived to discharge. Conclusion Mortality from OHCA remains high, even in a densely population urban area. By-stander BLS was rarely available. Ambulance response times remain too high.

**Henry 2013^50^** Objective: To characterize OHCA attended by the Ambulance Service. Methods: Retrospective observational Results: 231 patients; 56% urban and 44% in rural setting. Resuscitation attempted in 77.5%, 15% ROSC and 7.4% survived to leave hospital. Survival was 16.7%. Conclusion: Despite longer response times for rural compared with urban OHCAs, survival was similar.

**Herlitz 2006^51^** Objective: To describe the association between the interval between the call for ambulance and ROSC and survival in OHCA. Methods: Retrospective observational study. Results: 4 667 patients; if interval was < 5 min, 47% survived to one month. If > 30 min, 5% survival to one month. Conclusion: there is a very strong association between the interval between the call for ambulance and ROSC and survival to one month.

**Herlitz 2008^52^** Objective: To define factors associated with an improved outcome among OHCA in a non-shockable rhythm. Methods: Retrospective observational study. Results: 22 465 patients, 57% were witnessed, 64% cardiac etiology, 71% at home and 34% received bystander CPR. Survival to 1 month was 1.3%. The following were independently associated with an increased chance of survival: 1/Decreasing age, 2/Witnessed arrest, 3/Bystander CPR, 4/Cardiac arrest outside home, 5/Shorter ambulance response time and 6/Need for defibrillation. If these six criteria were fulfilled, survival to 1 month increased to 12.6%. Conclusion: The overall survival among patients with an OHCA found in a non-shockable rhythm is very low (1.3%). Six factors associated with survival can be defined.

**Hillis 1993^53^** Objective: To determine the effectiveness of defibrillation in prehospital cardiac arrest. Prospective cohort study. Defibrillation group vs no-defibrillation group. Results: 221 patients, experimental group (N = 161) and the control group (N = 60) were comparable. Survival to hospital discharge was 2/60 (3.3%) in the control group and 12/161 (6.3%) in the experimental group. Survivors had a good functional outcome. Conclusion: Defibrillation in OHCA significantly improves survival and outcome.

**Holmen 2020^54^** Objective: To assess the effect of ambulance response time on the outcome after OHCA. Methods: Retrospective observational study. Results: 20 420 patients; 30-day survival decreased as ambulance response time increased. Conclusions: Survival to 30 days after a witnessed OHCA decreases as ambulance response times increase. Shortening EMS response times is likely to be a fast and effective way of increasing survival in OHCA.

**Huabb. 2021^55^** Objective: To explore factors associated with successful resuscitation in OHCA. Methods: Retrospective descriptive study. Results: 273 patients. 25.6% ROSC. Conclusion: Four factors were found to have a significant association with successful prehospital cardiac resuscitation on scene. These factors will develop on-scene CPR guidelines.

**Hubert 2016^56^** Objectives: to describe the cohort of persons having experiences fatal and non-fatal drowning events. Methods: Prospective multicenter study. Results 234 patients. 33.8 % had an immediate basic life support. Advanced cardiac life support in 87.2%. Response time was 22 minutes. At hospital, 40.6% of patients were alive. 9.0% were discharged alive. Conclusions: The model is helpful to highlight explanative variables concerning drowning patients’ outcome.

**Jennings 2006^57^** Objective: To compare the survival rate from OHCA in rural and urban setting. Methods: Retrospective case series. Results: 1 790 patients; bystander CPR more likely in rural (65.7%) than urban areas (48.4%) (P = 0.001).). Factors associated with survival to hospital admission were distance of cardiac arrest from the closest ambulance, endotracheal intubation. Conclusions: Survival rates differ between urban and rural cardiac arrest patients. This is largely due to a difference in ambulance response time.

**Jeong 2017^58^** Objective: To evaluate the role of response time in improving survival for OHCA within a region. Methods: This study was a retrospective and observational analysis of data as Utstein style with registry of OHCA patients from 2012 to 2016. Results: 400 patients with OHCA were included. ROSC was achieved in 8.8% of patients and 9.5% of them survived to hospital discharge and 5.3% had favorable neurologic status at discharge. Response time was median 8.0 min (IQR 6.0 to 10.0), 8.0 min (IQR 5.0 to 10.0) min for survivors and 8.0 min (IQR 6.0 to 10.0) min for non-survivors. Conclusion: a shorter response time was not associated with survival to hospital discharge or with neurologic outcome at hospital discharge.

**Kennedy 2023^59^** Objective: To examine the impact of the COVID-19 pandemic on the incidence and survival outcomes of EMS-witnessed OHCA. Methods:  an interrupted time-series analysis of adult EMS-witnessed OHCA patients of medical etiology. Results: 5 034 patients, 79.0% in the comparator period and 21.0% in the COVID-19 period. Patients in the COVID-19 period had longer EMS response times, fewer public location arrests and were significantly more likely to receive mechanical CPR and laryngeal mask airways compared to the historical period.. Conclusion: changes during the COVID-19 pandemic did not influence incidence or survival outcomes in EMS-witnessed OHCA.

**Kentsch 2000^60^** Objectives: To analyze the effects of political and socioeconomic changes, patients receiving ALS for OHCA between 1984 and 1988, and from 1991 to 1997 were studied. Methods: retrospective observational study. Results: Survival without relevant neurologic defects was achieved in 3.7% of patients before 1989 and in 8.1% after 1990. Response time of the ALS unit shortened from 11.0 +/- 1.4 to 9.0 +/- 0.4 min (ns.), while response time of any EMS shortened from 11.0 +/- 1.4 to 6.1 +/- 0.3 min (P < 0.005). Conclusion: In parallel to socioeconomic changes, the restructuring of the EMS in Stralsund and the rapid expansion of the telephone network led to a significant increase in the number of patients successfully resuscitated from OHCA.

**Kitano 2022^61^** Objectives: to compare the 1-month survival rate of cardiac arrest witnessed by EMS cardiac arrest witnessed by bystanders and unwitnessed cardiac arrest in traffic trauma victims; further, the time from injury to cardiac arrest was assessed. Methods: This analysis used the Utstein Registry in Japan and included data of patients with traumatic cardiac arrest caused by traffic collisions registered between 2014 and 2019. Results: The 1-month survival rate was 10.9% in the EMS-witnessed cardiac arrest group. The median time from injury to cardiac arrest was 18 min. Conclusion:  1-month survival rate was significantly higher in the EMS-witnessed cardiac arrest group than in the bystander-witnessed and unwitnessed cardiac arrest groups.

**Klosiew. 2017^62^** Objective: To assess incidents of OHCA and prehospital frequency of ROSC after OHCA. Methods: Retrospective observational study. Results: ROSC in 68.88% of cases. Time from ambulance departure to arrival was 5.42 min. We did not find any statistically significant difference between the number of deaths and those parameters (p = 0.723, p = 0.891). However, longer team response time correlated with the highest mortality (p = 0.042, contingency factor = 0.126). In the group where ROSC was achieved, the median time of EMS response was 8.18 min, while among the group of deceased the median was 8.63 min. Conclusions: EMS response time does not affect the frequency of ROSC.

**Lee 2019^63^** Objective: To determine whether short EMS response time was associated with improved neurologic outcome in OHC. Methods: Prospective observational analysis. Results: 2 309 patients. Response time threshold was 11.5 min for prehospital return of spontaneous circulation and 7.5 min for survival to discharge and favorable neurologic outcome. Patients in the 7.5 min response time group showed increased odds of survival to discharge. When response time was decreased by 1 min, all outcomes were improved (survival to discharge, OR: 1.08; 95% CI: 1.04–1.12, p < .001; favorable neurological outcome, OR: 1.14, 95% CI: 1.07–1.21, p < .001). Conclusion: EMS response time threshold associated with improved favorable outcome was 7.5 minutes.

**Lee 2013^64^** Objective: to measure the outcomes of adult nontraumatic OHCAs treated with 5:1 compressions-to-ventilation or continuous chest compressions with ventilation). Methods: retrospective observational cohort study. Results: 515 included. The average ambulance transport time was 6.1 minutes in both phases. The rates of ROSC [35.1% vs. 23.5%; adjusted odds ratio (OR), 1.616; 95% confidence interval (CI), 1.073-2.432] and survival to hospital discharge (10.1% vs. 4.2%; adjusted OR 2.431; 95% CI, 1.15-5.12).. Conclusion: In an emergency department with short ambulance transport times, continuous chest compressions with ventilation showed improved outcomes.

**Leung 2001^65^** Objectives. To evaluate the effectiveness of the local emergency medical services system in resuscitation of OHCA and identify areas for improvement. Methods: prospective descriptive study of adults with non-traumatic OHCA. Results. 320 patients were included. The majority of cardiac arrests occurred at patients’ homes. In 57.5%, the arrest was not witnessed. The bystander CPR rate was 15.6%. The average call to dispatch interval was 1.04 minutes. The average call to CPR interval was 9.82 minutes. The average total prehospital interval was 27.55 minutes. The rate of survival to hospital discharge was 1.25%. Conclusion. The prognosis of out-of-hospital cardiac arrest was dismal. Every link in the chain of survival has to be improved.

**Lim 2020^66^** Objective: To examine differences in patient characteristics, prehospital care, and outcomes in OHCA. Methods: retrospective observational study. Results: 25 895 OHCA patients. Likelihood of survival increased significantly (P<0.001) with arrest in public locations witnessed arrest (AOR 2.14), bystander cardiopulmonary resuscitation, initial shockable rhythm), and bystander defibrillation but decreased with increasing age and response time. Conclusions: Survival differences might be related to different emergency medical services practices.

**Lin 2014^67^** Objectives: to determine the incidence of VT/VF as the first presenting arrhythmia in OHCA patients based on the Taichung Sudden Unexpected Death Registry (THUNDER) in Taiwan. Methods: Retrospective observational study. Results: 2 156 EMS-assessed OHCAs was analyzed. 1 759 OHCA cases constitute the study population. The median EMS response time from call help to arrival was 6.0 min and the AED machine-read shockable rhythm was registered in 7.7% of all study patients. Conclusions: A low incidence of VT/VF in patients with presumed cardiogenic OHCA according to both machine-read and cardiologist-adjudicated VT/VF rhythm.

**Little 2020^68^** Objectives: To understand the impact of COVID-19 on delivery and outcomes of PCI. Methods: Retrospective observational study. Results: There was a 21% reduction in STEMI admissions and longer ambulance response times. Conclusion: These findings suggest that PCI pathways can be maintained during unprecedented healthcare emergencies but confirms the high mortality of STEMI in the context of concomitant COVID-19 infection.

**Liu 2023^69^** Objective: To determine how the pandemic affected the EMS response and the clinical outcomes in OHCA patients in COVID-19 low-incidence cities. Methods: Retrospective observational stud. Results: 567 patients before and 497 during the pandemic were enrolled. Multivariate analysis revealed that the COVID-19 pandemic had no significant influence on ROSC and sustained ROSC but was associated with lower probabilities of survival to discharge. Ages and OHCA locations were also discovered to be independently related. Conclusion: The overall impact of longer EMS rescue times on survival outcomes during the pandemic was not significant.

**Lyon 2013^70^** Objective: to assess the impact of tasking a HEMS service to OHCA and characterize the nature of these calls. Method: Retrospective case review. Results: HEMS was activated 89 times to suspected OHCA, 11% of the total HEMS missions. 25 patients achieved ROSC, 13 (52%) prior to HEMS arrival. The median time from first collapse to HEMS arrival was 31 minutes. The median time from HEMS activation to arrival on scene was 17 minutes. The survival to discharge rate was 6.3%. Conclusion: OHCA represents a significant proportion of HEMS call outs. HEMS are rarely first on-scene and should only be tasked as a first response to OHCA in remote locations.

**Margey 2011^71^** Objective: To describe the incidence and survival from OHCA, and the impact of improved pre-hospital care on survival from OHCA. Methods: Retrospective observational study. Results: 962 patients were included. Survival to hospital discharge improved significantly from 2.6 to 11.3%, P=0.001. Survival to hospital discharge from VF improved from 21.4 to 33%, P= 0.007. Mean EMS response times to the scene of arrest decreased from 9.18 to 8.34 min. Conclusions: Survival to hospital discharge has improved dramatically. Reduction in ambulance response time was associated with an increase in the proportion of victims found in VF rather than asystole and likely accounted for most of the improvement.

**Mathiesen 2018**^72^Objective: To study how such factors influenced OHCA survival in a mixed urban/rural region. Methods: Retrospective observational study. Results: 1 138 patients. Significantly higher probability for survival to hospital admission in the urban group versus the rural group. Bystander CPR was associated with increased patient survival to discharge only in urban areas (survival probability 0.26 with CPR vs. 0.08 without CPR, p < 0.001). EMS response time ≥ 10 min was associated with decreased survival (OR: 0.61, 95% CI 0.45-0.83, p = 0.002), Conclusions: OHCA survival was higher in urban compared to rural areas, and the effect of bystander CPR, EMS response time and EMS physician attendance on survival differ between urban and rural areas.

**Mayer 1979^73^** Objective: To assess if minimizing response time is an important goal in EMS. Methods: retrospective observational study. Results: 525 OHCA patients. The relationship between response time and survival determined. Conclusion: Paramedic response time is confirmed to be related statistically to long and short term survival from ventricular fibrillation.

**Meyer 2001^74^** Objective: To study the outcome of victims of OHCA in asystole. Methods: Retrospective observational study. Results: 778 patients. Response time was a mean of 9.76 min. Resuscitation was commenced on 37% of patients. There was one survivor (0.12%). Conclusion: Adult victims of out-of-hospital cardiac arrest presenting as asystole should not receive treatment.

**Mikiewicz 2023^75^** Introduction: The aim of this study was to analyze the epidemiology and outcomes of patients with OHCA in the Polish Tatra Mountains. Methods: Retrospective analysis of data 2001 to 2021.Results—A total of 74 cases of sudden cardiac arrest were recorded. The mortality rate was 88% (65/74). Return of spontaneous circulation was achieved in 22 (30%) patients. All survivors had a shockable initial rhythm. The majority of survivors (8/9, 89%) had a good or moderate neurological outcome. Conclusions—This study confirms poor survival rate after sudden cardiac arrest in the mountain area. The use of AED, shockable initial rhythm, and shorter time interval to emergency team arrival and ALS initiation are associated with better outcome.

**Mikkelsen 2017^76^** Objective: To describe the workload of a prehospital anesthesiologist-manned MECU and the total population it services in terms of factors associated with mortality. Methods: Retrospective observational study. Results: 32 873 patients 30-day mortality was 5.7%, 90-day mortality was 8.1% while 2-year mortality was 16.4% (Increasing age, male sex, comorbidity and prior admission to hospital but not response time were associated with mortality. Conclusions: Mortality following a MECU contact was high in the first 2 years following the incident. MECU response time assessed as a continuous parameter was not associated with patient outcome.

**Mills 2023^77^** Objective To examine the association between the response times of ambulances and 30-day mortality. Methods: Retrospective observational study. Results: 182 895 patients. Results Unadjusted, short response times were associated with higher 30-day mortality rates. In OHCA, longer response times of up to 10 min correlated with increased 30-day mortality rates. Conclusion: Longer ambulance response times were not associated with increased mortality, except for OHCA.

**Mills 2019^78^** Objectives: To examine the association between time from emergency medical service vehicle dispatch to hospital arrival and mortality. Methods: Retrospective observational study. Results**:**93 167 individuals with highest priority ambulances dispatched, 1 948 (2.1%) were dead before the ambulance arrived and 19 968 (21.4%) were transported to the hospital under highest priority (median total prehospital time from dispatch to hospital arrival 47 min. Among 18 709 with population data, 1-day mortality was 10.9%, Thirty-day mortality was 18.3%. Conclusions: Time from emergency dispatch to hospital arrival mainly was <80 min, there was no overall relation between this prehospital time measure and mortality.

**Mogensen 2015**^79^Objectives: to assess the outcome of attempted pre-hospital cardiac resuscitations in the period from 2004-2007 and compare to previous studies. Methods: Retrospective observational study. Results: 289 cases in cardiac arrest, resuscitation was attempted in 279 and 200 of those were presumed to have a cardiac etiology. Average response time was 6.3 min. 54% survived to hospital admission, 25% survived to discharge. Bystander CPR was provided in 62% of witnessed cases. 60% were witnessed cases of which 31% survived to hospital discharge compared to 8%of non-witnessed cases. Conclusion: 1/4 cardiac arrest patients survives to discharge. Survival was found to be significantly higher if the cardiac arrest was witnessed.

**Morrisey 1996^80^** Objectives: To summarize all 2 550 trauma-related rural ambulance trip reports filed for the period January 1 through December 31, 1991. Methods: Retrospective observational study. Results: There were 13.1 trauma-related ambulance runs per 1,000 population. The mean response time was 8.5 minutes and in 90% of all rural trauma runs the ambulance arrived in 17 minutes or less. Only 51.5% of runs had a rural hospital as a destination, 14.2% went directly to a trauma center, and nearly 20% to another urban hospital. Of the 71 severe trauma cases received by ambulance, rural hospitals transferred out only 13 cases, most of these to the regional trauma center. Of the 47 trauma cases transferred to the trauma center, 33 were not severe. Conclusion: Relevant data has been described.

**Nadolny 2021^81^** Objectives: to assess the usefulness of the Glasgow Coma Scale (GCS) score assessed by EMS team in predicting survival to hospital discharge in patients after out-of-hospital cardiac arrest (OHCA). Methods: retrospective register study. Results: 218 patients with OHCA, who achieved ROSC, were included. ROC analysis revealed GCS = 4 as a cut-off value in predicting survival to discharge. Variables significantly associated with in-hospital survival were young age, short response time, witnessed event, previous myocardial infarction, chest pain before OHCA, initial shockable rhythm, coronary angiography, and GCS > 4. Conclusion: The survival to hospital discharge after OHCA could be predicted by the GCS score on hospital admission.

**Naroo 2012^82^** Objectives: To study correlation of response time with the survival rate of OHCA. Retrospective observational study. Methods: Retrospective observational study. Results: 60 patients. At 30 days of follow up, 4 cases survived. Among those who survived, 2 cases had response time of 01 minute. EMS response time is 7.7 minutes. Conclusion: A lesser response time improves chance of survival for OHCA.

**Navab 2019^83^** Objective: To identify important factors contributing to ROSC and survival in OHCA. Methods: Retrospective observational study. Results: 3 214 patients, 26.3% witnessed, with only 5.1% bystander-initiated CPR. Furthermore, the median ambulance response time 6.0 minutes. Overall, ROSC and survival to discharge success rates were 8.3 and 4.1%, respectively. Bystander CPR was the most effective predicting factor for the success rate of ROSC. Conclusion: The age, ambulance response time, CPR duration, and cardiac disease history were negatively associated with the outcomes of ROSC and SHD, while being witnessed, bystander CPR, ETI, and initial shockable rhythm were positively related to survival.

**Nichol 2016^84^** Objective: To assess whether time from receipt of a call to dispatch of EMS providers was associated with outcomes. Methods: Retrospective observational study. Results: 2 687 patients, activation interval was mean 1.2±0.6min. Response interval was mean 6.1±2.4min. 45.9% achieved ROSC; 17.7% survived to discharge; and 15.9% had favorable neurologic status. Conclusions: Briefer activation interval was independently associated with greater survival.

**Nielsen 2022^85^** Objectives: To examine the diagnostic pattern, level of severity of illness or injuries, and mortality among children for whom a physician-staffed HEMS was dispatched. Methods: Population-based cohort study including patients aged less than 16 years treated by the Danish national HEMS. Results: 651 HEMS missions included pediatric patients aged less than 1 year (9.2%), 1 to 2 years (29.0%), 3 to 7 years (28.3%), and 8 to 15 years (33.5. Twenty-nine patients died either on or within 1 day of the mission, and the cumulative 30-day mortality was 35 of 565. Conclusion: Among hospitalized patients, nearly one-fifth of the patients required immediate intensive care and 6.2% died within 30 days of the mission.

**O’Keefe 2011^86^** Objectives: To evaluate the role of ambulance response times in improving survival for OHCA. Methods: Retrospective observational study. Results: 1 161 patients, 2.6% survived to hospital discharge. If the patient arrested while the paramedics were on scene, survival to hospital discharge was 14%. The most important predictive factors for survival were response time, initial presenting heart rhythm in ventricular fibrillation and whether the arrest was witnessed. Conclusions: The arrival of a crew prior to OHCA means that the chance of surviving the arrest increases sevenfold.

**Park 2017^87^** Objectives: to determine the impact of bystander CPR on clinical outcomes in patients with increasing response time from collapse to EMS response. Methods: A population-based observational study of patients with witnessed OHCA. Results: 15,354 OHCAs were analyzed. Bystander CPR was performed 56%. Survival to hospital discharge occurred in 10,6% and favorable neurological outcome in 6,5%. In an interaction model of bystander CPR, compared to the fastest group, adjusted odds ratios (AORs) (95% CIs) for survival to discharge were 0.89 (0.66-1.20) in the fast group, 0.76 (0.57-1.02) in the late group, and 0.52 (0.37-0.73) in the latest group. Conclusion: The survival from OHCA decreases as the ambulance response time increases.

**Pell 2001^88^** Objectives: To determine the association between ambulance response time and survival from OHCA and to estimate the effect of reducing response times. Methods: Retrospective observational study. Results: 10 554 patients, 6% survived to hospital discharge. Shorter response time was significantly associated with increased probability of receiving defibrillation and survival to discharge among those defibrillated. Conclusions**:**Reducing ambulance response times to 5 minutes could almost double the survival rate for cardiac arrests not witnessed by ambulance crews.

**Pons 2005^89^** Objectives: To evaluate the effect of paramedic response time on survival to hospital discharge in OHCA. Methods: Retrospective observational study. Results: 9 559 patients. A survival benefit was identified for response times <or=4 minutes. No survival benefit was identified when response time was modeled as a continuous variable or when dichotomized at 8 minutes. Conclusions: A survival benefit was identified when the response time was within 4 minutes for patients with intermediate or high risk of mortality.

**Pons 2002^90^** Objective: To assess the effect of exceeding the 8 min RT guideline on patient survival for trauma. Methods: retrospective observational study. Results: 3 490 patients were grouped according to ambulance RT: < or = 8 min (n = 2 450) or > 8 min (n = 1 040). After controlling for other significant predictors, there was no difference in survival after traumatic injury when the 8 min ambulance RT criteria was exceeded. Conclusions: Exceeding the ambulance industry response time criterion of 8 min does not affect patient survival after traumatic injury.

**Puolakka 2023^91^** Objectives: to describe the ECPR programme for OHCA by reporting the number of ECPR protocol activations. Methods: observational cohort study. Results: 73 cases of normothermic OHCA. The mean patient age was 54 years and 91.8% of them were male. The median ambulance response time (IQR) was 9 (7-11) min. Thirteen (35.1%) of them survived to discharge and 29.7% with a cerebral performance category (CPC) 1-2. In those ECPR candidates who did not receive ECMO, 8 (22.2%) received permanent ROSC during transport or immediately after hospital arrival and 6 (16.7%) survived to discharge with a CPC 1-2. Conclusions: Half of the ECPR protocol activations did not lead to ECMO treatment.

**Do 2010^92^** Objectives: To describe the impact of response interval on sustained ROSC. Methods: Retrospective observational study. Results: 2 432 patients. 32.0%) achieved sustained ROSC. The mean response interval was significantly shorter for patients who obtained sustained ROSC (370 seconds) than for patients who did not (394 seconds) (p = 0.015). Conclusion: A significantly shorter response interval was observed in patients who were successfully resuscitated after OHCA.

**Rajan 2016^93^** Objective: to examine the association of bystander CPR with survival as time to advanced treatment increases. Methods: Retrospective observational study. Results: 7 623 patients. With increasing response times, adjusted 30-day survival chances decreased for both patients with bystander CPR and those without. Conclusions: The absolute survival associated with bystander CPR declined rapidly with time. Yet bystander CPR while waiting for an ambulance was associated with a more than doubling of 30-day survival even in case of long ambulance response time. Decreasing ambulance response time by even a few minutes could potentially lead to many additional lives saved every year.

**Renkiewicz 2014^94^** Objective: To develop a model describing the likelihood of shockable presenting rhythm as a function of EMS response time. Methods: Retrospective observational study. Results: 599 patients were included. VF/VT was observed in 26.5%. VF/VT was less likely with increasing EMS response time and age. Bystander CPR was not associated with shockable presenting rhythm, although EMS response time was longer among patients with bystander CPR compared to those without (9.83 vs. 8.83 minutes, p < 0.01). Conclusions: We found that for every one-minute of added ambulance response time, the odds of shockable presenting rhythm declined by 8%.

**Schinnerl 1990^95^** Objectives: To analyze the outcome of OHCA during the first 3 years after installation of a MICU. Methods: Retrospective observational study. Results: 89 patients (32.96%) had a temporary cardiac output; 56 patients (20.74%) were primarily successfully resuscitated; and 12 patients (4.44%) survived without important neurological sequelae. Conclusion: Our study shows that in spite of installing a MICU, the outcome of CPR is poor without supplementary measures.

**Semensato 2011^96^** Objective: To evaluate clinical predictors of survival of patients in OHCA. Methods: Retrospective observational design. Results: 593 patients. 6.0% alive on the 30th day and 3.9% discharged. The response-time and collapse-time intervals until CPR start were significantly shorter in 30-day survivors. Conclusion: It is necessary to reinforce each link of the survival chain to improve pre-hospital care, aiming at improving clinically relevant outcomes.

**Shah 2010^97^** Objectives**:**To compare out-of-hospital cardiac arrest characteristics in white and South Asian populations within Greater London. Methods: Data for OHCAs were extracted from 1 April 2003 to 31 March 2007. Results**:**Of 13 013 OHCAs of presumed cardiac cause, 3161 (24.3%) had ethnicity codes assigned. These comprised 63.1% white and 5.8% South Asian people, with the remainder from other backgrounds. Response time (7.48 min vs 7.46 min), bystander CPR (34.4% vs 29.7%), initial cardiac rhythm (29.5% vs 30.4%) and survival to admission (22.2% vs 22.5%) and discharge (8.7% vs 8.9%) were comparable between the two ethnic groups. Conclusion**:**The quality of care provided was comparable between white and South Asian populations.

**Shepherd 2008^98^** Objective: To assess patient injury severities and outcomes and to determine any time advantage of the aircraft over ground transfer. Methods: Retrospective observational from January 2004 to November 2006. Results: 222 missions were identified from the helicopter log. Air transport was significantly faster beyond 100 km, with a mean difference of 48 min. Conclusion: We could not identify a significant survival benefit attributable to the addition of a doctor, although numbers for this comparison were small.

**Sigursson 2000**^99^Objective: To evaluate the influence ACLS service and of bystanders on survival in OHCA. Methods: Retrospective observational study. Results: 308 patients. The mean response time was 4.6 min. Patients admitted to the intensive or cardiac care was 31% and 17% were discharged from the hospital. Bystanders were present in 68% of cases and it fourfoulded the likelihood of discharge. Conclusions: When OHCA is witnessed, the probability of survival is multiplied.

**Sipria 2016^100^** Objectives: To evaluate survival to discharge from hospital in patients resuscitated from bystander witnessed OHCA. Methods. A prospective observational cohort study of OHCA. Results:3 335 patients, 10.2% were discharged alive in CPC 1, 2. For the group of patients admitted to hospital, the median response time interval in the survival group was 6 min compared with 7 min for patients who died in hospital (p < 0.0001) and median resuscitation time was 11 min compared with 21 min (p < 0.0001), respectively. Compared with the patients under the age of 40 years, the chance to survive was significantly lower among the patients aged between 40-60 years and over 60 years. Conclusions. In Estonia, the survival of patients resuscitated from bystander witnessed OHCA increased.

**Spaite 2008^101^** Objectives: To examine whether prolonging transport to reach a designated facility would be detrimental. Methods: Retrospective observational study. Results: 1 846 OHCA occurred prior to EMS arrival. 17.0% comatose ROSC patients survived. Mean transport interval for the study group was 6.9 min. Logistic regression revealed factors that were independently associated with survival: witnessed arrest, bystander CPR, method of CPR, initial rhythm of ventricular fibrillation, and shorter EMS response time interval. Conclusion: Survival was not significantly impacted by transport interval. This suggests that a modest increase in transport interval from bypassing the closest hospital en route to specialized care is safe and warrants further investigation.

**Stoeckl 2010^102^** Objectives: To assess if response intervals influence survival and outcome in OHCA. Methods: Retrospective observational study. Results: 552 patients included. Medical call processing and dispatch took 02:36 min in the survival group and 02:28 min for non-survivors. EMS response times aggregate to 08:31 min for survivors and 09:04 min for non-survivors (p > 0.01) Conclusion: Medical call processing and dispatch times seems to be long and showed no differences between outcome groups; same could be found for ambulance arrival at patients’ site.

**Stromsoe 2015^103^** Objectives: To describe OHCA in Sweden. Methods: All cases of OHCA (n = 59 926) reported to the Swedish Cardiac Arrest Register were included. Results: Thirty-day survival increased from 4.8 (1992) to 10.7% (2011). Among patients hospitalized with ROSC in 2008-2011, 41% underwent therapeutic hypothermia and 28% underwent PCI. Among 30-day survivors in 2008-2011, 94% had a cerebral performance category score of 1 or 2 at discharge from hospital and the results were even better if patients were found in a shockable rhythm. Conclusion**:**From a long-term perspective, 30-day survival after OHCA in Sweden more than doubled. The increase in survival was most marked among patients found in a shockable rhythm and those hospitalized with ROSC.

**Stromsoe 2011^104^**Objective: To describe the reported incidence of OHCA) and the characteristics and outcome after OHCA in relation to population density. Methods: Retrospective observational study. Results: 9 979 patients. Ambulance response time was longer in less populated areas (p<0.0001). There was no significant association between population density and survival to 1 month after OHCA or incidence (adjusted for age and gender) of OHCA. Conclusion: Bystander CPR, cardiac etiology and longer response times were more frequent in less populated areas.

**Sugiyama 2023**^105^Objective: To identify whether COVID-19 pandemic affects the operational efficiency of EMS and the survival rate of OHCA. Methods: Retrospective observational study. Results: 3 367 patients. The total out-of-service time, occupancy rate, and response time significantly increased during the pandemic period (p < 0.001). 1-month survival significantly. Similarly, 24-h survival (9.9% vs. 12.8%), and favorable neurological outcomes significantly decreased during the pandemic period. In the logistic regression analysis, response time was associated with lower OHCA survival in all outcomes (p < 0.05). Conclusion: The COVID-19 pandemic has been associated with reduced operational efficiency of EMS and decreased OHCA survival rates.

**Syvaoja 2018^106^** Objectives: To examine the impact of OHCA recognition in the EMCC on survival rates. Methods: Retrospective observational study. Results: Achieved ROSC and survival to hospital discharge were 49% and 23%, respectively, if cardiac arrest was recognized by the EMCC and 40% and 16% when it was not. Dispatchers gave CPR instructions in 60% of the recognized OHCA cases. Bystander-performed CPR increased over time and was given in 58% of the recognized OHCA. EMS delays were shorter if OHCA was recognized as opposed to unrecognized. Conclusions: Recognition of OHCA by the EMCC was significantly associated with an increased rate of bystander-performed CPR, reduced EMS response time, and increased OHCA patient ROSC and survival rates.

**TerAvest 2019^107^** Objectives: to investigate the added value of HEMS in the treatment of TCA. Methods**:** a retrospective cohort study of all patients with a pre-hospital TCA. Results:  263 patients with a TCA were attended by HEMS with an average response time of 30 min. 20% regained ROSC at scene. Most patients who had a ROSC had one or more HEMS specific interventions being performed. HEMS also delivered other important interventions to these patients as IV/IO access and endotracheal intubation without drugs. Conclusion: HEMS teams should be involved in the treatment of patients with a TCA, even in non-urban areas with prolonged response times, as they provide knowledge and skills that contribute to regaining and maintaining a sustained ROSC.

**Thompson 2017^108^** Objective: To identify pre-hospital factors associated with patient outcomes for major trauma. Method: Retrospective observational study. Results: 1 033 patients. GCS, Respiration Rate and Age were all significant when associated with the outcome. Conclusion: GCS, respiration rate and age are predictive triggers for transport to Trauma Centre.

**Trenkler 2012^109^** Objectives: to develop regional registry with the goal to test the approach and to benchmark our service. Methods: Retrospective observational study. Results: The mean response time of ambulance was 8.6 min. In 73% the etiology was primary cardiac. The initial rhythm was asystole in 58.2%, VF in 29.1% patients, PEA in 10.5%; in 2.1% the rhythm was not identified. ROSC was obtained in 32,5% patients. One month survival was 6.7%. Conclusions: In preparation to join the EuReCa in this pilot study we have defined the Slovak terminology, trained the personnel to fill the formulary in unified fashion and improved the software.

**Tsai 2017^110^** Objectives: Predicting the outcome of OHCA patients is crucial. Methods**:**Retrospective observational study. Results: Multivariate analysis showed that hospitalization with immediate PCI availability was an independent predictor solely for the outcome of survival until discharge. The presence of a witness while collapsing, EMS response time, and scene time interval were valuable for predicting the neurological outcome. Conclusions: Direct ambulance delivery to intensive heart hospitals that had 24/7 PCI availability was associated with a higher probability of surviving until discharge. Longer response time and scene time interval indicated poorer survival and neurological outcome.

**Wai 2018^111^** Objective: To describe OHCA and evaluate survival. Design: Retrospective observational study. Results: 124 patients. Bystander CPR rate was 15.3%.. Survival was 0.8% and survival to hospital discharge was significantly higher for patients with VF or pulseless VT The median witnessed/recognized collapse to defibrillation time was 14 minutes. The median prehospital time interval from collapse/recognition to arrival at hospital was 33 minutes. Conclusion: The prognosis of out-of-hospital cardiac arrest in Hong Kong was poor.

**Weinlich 2019^112^** Objectives: To analyze HEMS in comparison to EMS, in respect to patient's mortality and morbidity. Methods: Retrospective observational study. Results: 1 646 patients. 7.8% died in the hospital. Unadjusted mortality was significantly lower in the HEMS group compared to EMS. Conclusions: A significant improvement for in-hospital survival for HEMS could be demonstrated.

**Wik 2003^113^** Objective: To determine the effects of CPR before defibrillation on outcome in patients with VF and with response times either up to or longer than 5 minutes. Methods: Randomized controlled trial, CPR before defibrillation vs. standard. Results: There were no differences in ROSC rates between the groups. 89% survived to hospital discharge with no or minor reductions in neurological status with no difference between the groups. Conclusions: Compared with standard care for ventricular fibrillation, CPR first prior to defibrillation offered no advantage in improving outcomes for this entire study population or for patients with ambulance response times shorter than 5 minutes.

**Wissa 2021^114^** Objective: To examine the association between time to amiodarone and survival in OHCA patients. Methods: Retrospective observational study. Results: 502 patients were included. The average time from arrest to amiodarone was 25 min. Time to amiodarone was negatively associated with survival. The optimal time window for amiodarone was within 23 min following arrest. Paramedic response time and time from arrest to intravenous access were independent factors determining whether patients received amiodarone within the optimal time. Conclusions: Earlier amiodarone administration was associated with improved survival.

**Zheng 2023**^115^ Background: OHCA is an important global public health issue, but its epidemiology and outcomes in low-income and middle-income countries remain largely unknown. Methods In the prospective, multicentre, population-based Baseline Investigation of Out-of-hospital Cardiac Arrest (BASIC-OHCA) registry study. The median EMS response time was 12 min (IQR 9–16). At hospital discharge or 30 days, 441 (1·2%) of 38 227 survived, 304 (0·8%) survived up to 6 months, and 269 (0·7%) up to 12 months. At hospital discharge or 30 days, 309 (0·8%) survived with favourable neurological outcomes, 257 (0·7%) had favourable neurological outcomes at 6 months, and 236 (0·6%) at 12 months.

**Legend**: ACLS: Advanced cardiac life support; ALS: Advanced life support; AOR: adjusted odds ratio; BCPR: Basic cardiopulmonary resuscitation; BLS: Basic life support; CAD: cardiac arrest after drowning; CI: confidence interval; COVID-19: Corona virus disease 2019; CPC: Cerebral performance category; CPR: cardio-pulmonary resuscitation; ECMO: Extracorporeal membrane oscillation; ECPR: Extracorporeal cardiopulmonary resuscitation; ED: emergency department; EMCC: Emergency medical communication centre; EMDC: Emergency medical dispatch centre; EMS: Emergency medical service; GCS: Glasgow Coma Scale; GEMS: Ground EMS; HEMS: Helicopter EMS; LOS: length of stay; IQR: interquartile range; ISS; Injury severity score; MECU: Mobile emergency care unit; MICU: Mobile intensive care unit; Mins.: minutes; MVC: motor vehicle crash; OHCA: Out of hospital cardiac arrest; OR: Odds ratio; PEA: pulseless electric activity; PEMS: Paramedic staffed EMS; PCI: Primary percutaneous coronary intervention; p: probability; REBOA: resuscitative endovascular balloon occlusion of the aorta; ROSC: Return of spontaneous circulation; RT: response time; RTI: Response time interval; SD: standard deviation; SHD: Survival to hospital discharge; STEMI: ST-elevation myocardial infarction; TBI: Traumatic brain injury; TCA: Traumatic cardiac arrest; VF: Ventricular fibrillation; VT: Ventricular tachycardia

**Supplemental Figure 1: Sub-group forest plots**

Subgroup analysis 1: Drowning

**
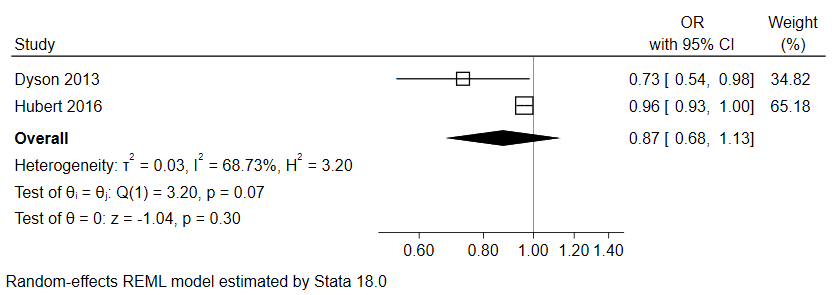
**

Subgroup analysis 2: Traumatic Out-of-Hospital Cardiac Arrest


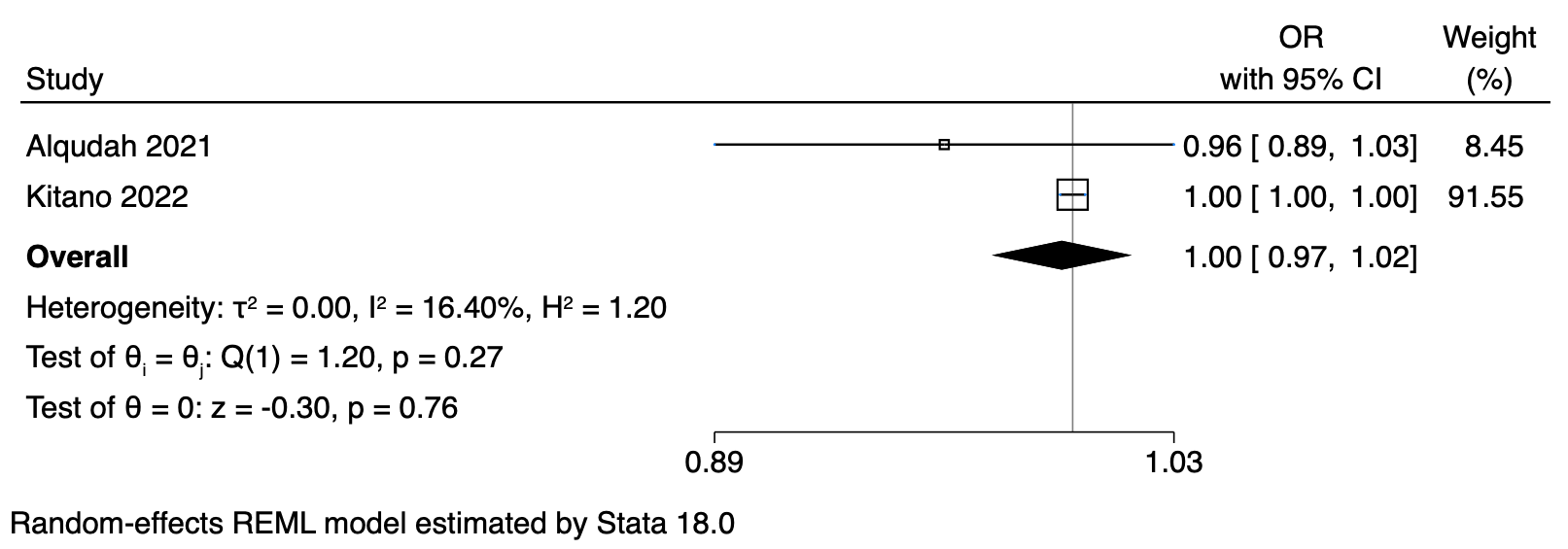


Subgroup analysis 3: Miscellaneous


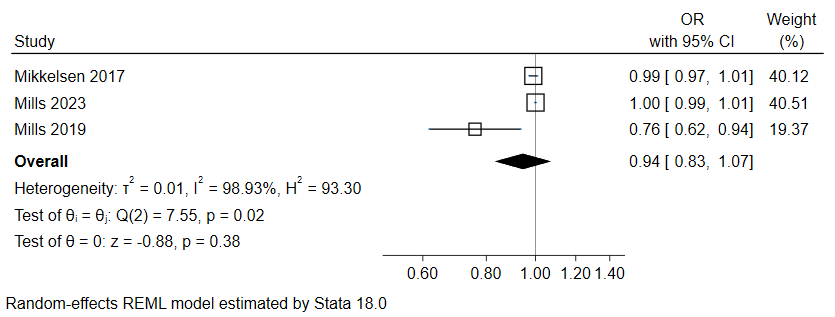


Subgroup analysis 4: Trauma


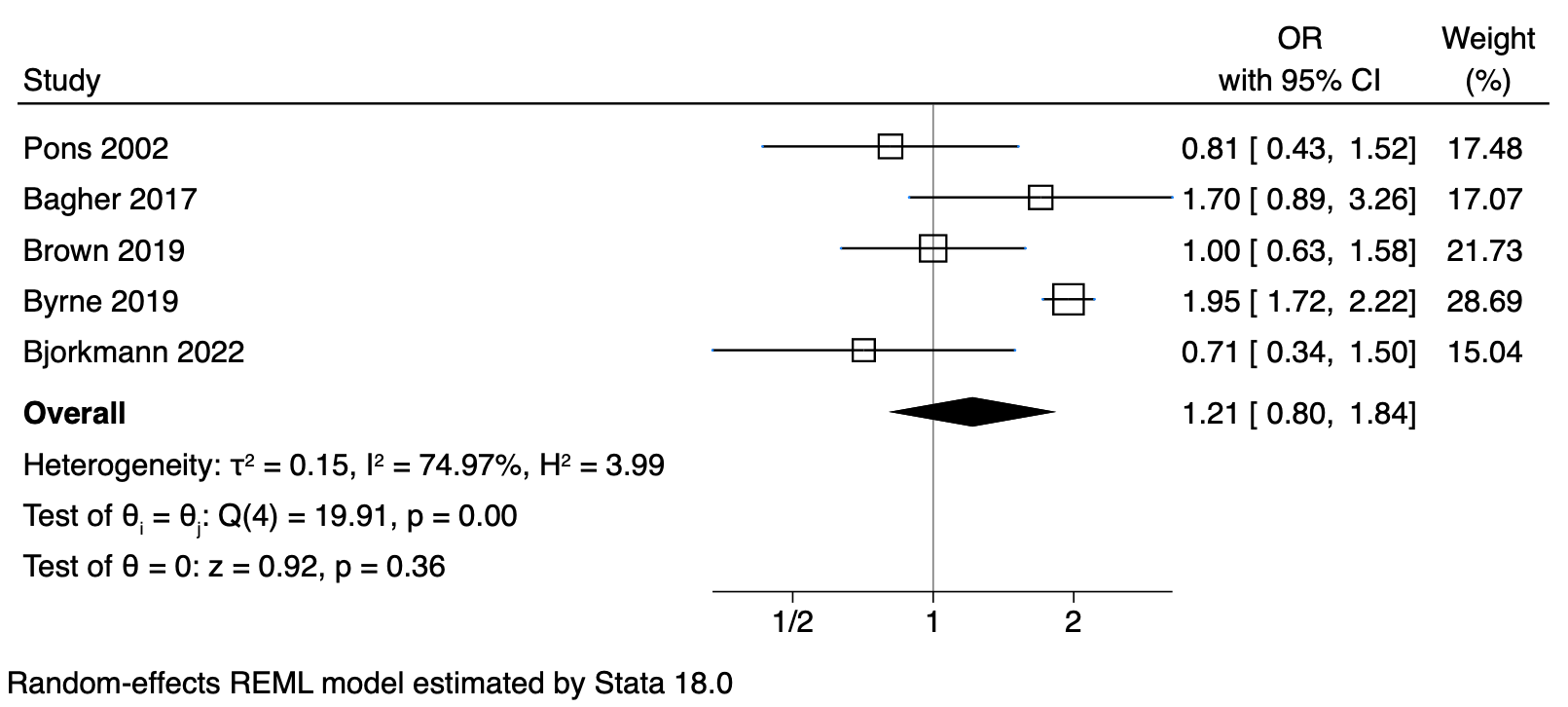


Subgroup analysis 5: Out-of-Hospital Cardiac Arrest

**
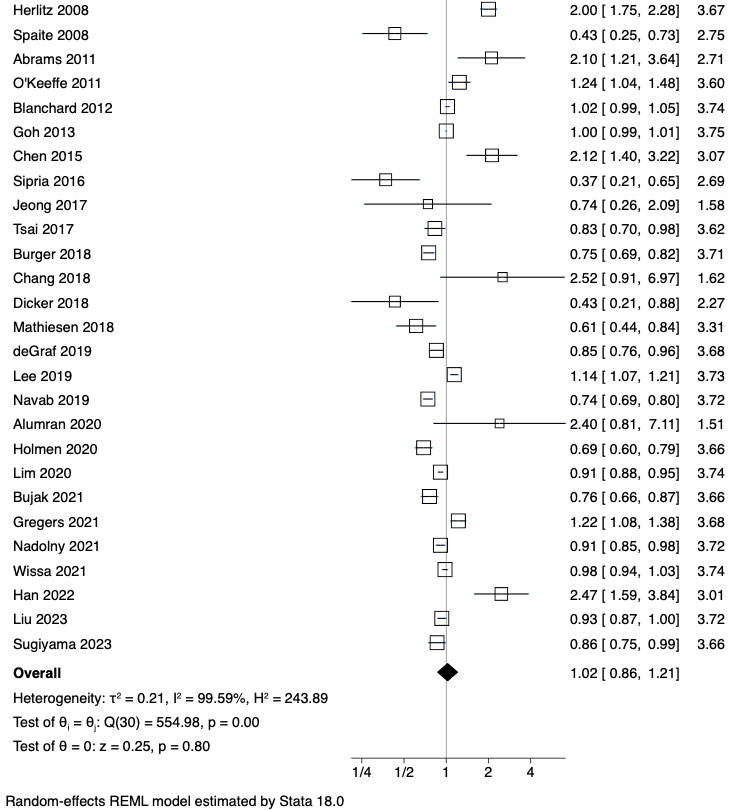
**

# PRISMA 2020 checklist

Completed PRISMA checklist

**Preferred Reporting Items for Systematic reviews and Meta-Analyses (PRISMA) Checklist**

| **Section and Topic** | **Item #** | **Checklist item** | **Location where item is reported** |
| --- | --- | --- | --- |
| **TITLE** | | |  |
| Title | 1 | Identify the report as a systematic review. | p. 1 |
| **ABSTRACT** | | |  |
| Abstract | 2 | See the PRISMA 2020 for Abstracts checklist. | p. 3 |
| **INTRODUCTION** | | |  |
| Rationale | 3 | Describe the rationale for the review in the context of existing knowledge. | p. 4 |
| Objectives | 4 | Provide an explicit statement of the objective(s) or question(s) the review addresses. | p. 5 |
| **METHODS** | | |  |
| Eligibility criteria | 5 | Specify the inclusion and exclusion criteria for the review and how studies were grouped for the syntheses. | p. 6, Figure 1 |
| Information sources | 6 | Specify all databases, registers, websites, organisations, reference lists and other sources searched or consulted to identify studies. Specify the date when each source was last searched or consulted. | p. 5, Supplemental methods p. 2-6 |
| Search strategy | 7 | Present the full search strategies for all databases, registers and websites, including any filters and limits used. | Supplemental methods p. 2-6 |
| Selection process | 8 | Specify the methods used to decide whether a study met the inclusion criteria of the review, including how many reviewers screened each record and each report retrieved, whether they worked independently, and if applicable, details of automation tools used in the process. | p. 5 |
| Data collection process | 9 | Specify the methods used to collect data from reports, including how many reviewers collected data from each report, whether they worked independently, any processes for obtaining or confirming data from study investigators, and if applicable, details of automation tools used in the process. | p. 7 |
| Data items | 10a | List and define all outcomes for which data were sought. Specify whether all results that were compatible with each outcome domain in each study were sought (e.g. for all measures, time points, analyses), and if not, the methods used to decide which results to collect. | p. 6  Supplemental methods p. 7 |
|  | 10b | List and define all other variables for which data were sought (e.g. participant and intervention characteristics, funding sources). Describe any assumptions made about any missing or unclear information. | p. 6, Supplemental methods p. 7 |
| Study risk of bias assessment | 11 | Specify the methods used to assess risk of bias in the included studies, including details of the tool(s) used, how many reviewers assessed each study and whether they worked independently, and if applicable, details of automation tools used in the process. | p. 7-8, Supplemental methods p.8 |
| Effect measures | 12 | Specify for each outcome the effect measure(s) (e.g. risk ratio, mean difference) used in the synthesis or presentation of results. | p. 7 |
| Synthesis methods | 13a | Describe the processes used to decide which studies were eligible for each synthesis (e.g. tabulating the study intervention characteristics and comparing against the planned groups for each synthesis (item #5)). | p. 7 |
|  | 13b | Describe any methods required to prepare the data for presentation or synthesis, such as handling of missing summary statistics, or data conversions. | p. 7-8 |
|  | 13c | Describe any methods used to tabulate or visually display results of individual studies and syntheses. | p. 7 |
|  | 13d | Describe any methods used to synthesize results and provide a rationale for the choice(s). If meta-analysis was performed, describe the model(s), method(s) to identify the presence and extent of statistical heterogeneity, and software package(s) used. | N/A |
|  | 13e | Describe any methods used to explore possible causes of heterogeneity among study results (e.g. subgroup analysis, meta-regression). | N/A |
|  | 13f | Describe any sensitivity analyses conducted to assess robustness of the synthesized results. | N/A |
| Reporting bias assessment | 14 | Describe any methods used to assess risk of bias due to missing results in a synthesis (arising from reporting biases). | N/A |
| Certainty assessment | 15 | Describe any methods used to assess certainty (or confidence) in the body of evidence for an outcome. | p. 7 |
| **RESULTS** | | |  |
| Study selection | 16a | Describe the results of the search and selection process, from the number of records identified in the search to the number of studies included in the review, ideally using a flow diagram. | p. 8, Figure 1 |
|  | 16b | Cite studies that might appear to meet the inclusion criteria, but which were excluded, and explain why they were excluded. | p. 8, Figure 1 |
| Study characteristics | 17 | Cite each included study and present its characteristics. | Supplemental tables 4 – 9, references |
| Risk of bias in studies | 18 | Present assessments of risk of bias for each included study. | p. 10, Supplemental table 3 - 4 |
| Results of individual studies | 19 | For all outcomes, present, for each study: (a) summary statistics for each group (where appropriate) and (b) an effect estimate and its precision (e.g. confidence/credible interval), ideally using structured tables or plots. | p. 8-10, table 3, Suppl. table 5 - 6 |
| Results of syntheses | 20a | For each synthesis, briefly summarise the characteristics and risk of bias among contributing studies. | p. 10-11, table 3-4 |
|  | 20b | Present results of all statistical syntheses conducted. If meta-analysis was done, present for each the summary estimate and its precision (e.g. confidence/credible interval) and measures of statistical heterogeneity. If comparing groups, describe the direction of the effect. | p. 6, Figure 3 |
|  | 20c | Present results of all investigations of possible causes of heterogeneity among study results. | N/A |
|  | 20d | Present results of all sensitivity analyses conducted to assess the robustness of the synthesized results. | N/A |
| Reporting biases | 21 | Present assessments of risk of bias due to missing results (arising from reporting biases) for each synthesis assessed. | N/A* |
| Certainty of evidence | 22 | Present assessments of certainty (or confidence) in the body of evidence for each outcome assessed. | p. 10-11, table 3-4 |
| **DISCUSSION** | | |  |
| Discussion | 23a | Provide a general interpretation of the results in the context of other evidence. | p. 11 |
|  | 23b | Discuss any limitations of the evidence included in the review. | p. 11-13 |
|  | 23c | Discuss any limitations of the review processes used. | p. 13 |
|  | 23d | Discuss implications of the results for practice, policy, and future research. | p. 13 |
| **OTHER INFORMATION** | | |  |
| Registration and protocol | 24a | Provide registration information for the review, including register name and registration number, or state that the review was not registered. | p. 5 |
|  | 24b | Indicate where the review protocol can be accessed, or state that a protocol was not prepared. | p. 5 |
|  | 24c | Describe and explain any amendments to information provided at registration or in the protocol. | p. 6,, supplemental methods p. 5 |
| Support | 25 | Describe sources of financial or non-financial support for the review, and the role of the funders or sponsors in the review. | p. 15 |
| Competing interests | 26 | Declare any competing interests of review authors. | p. 15 |
| Availability of data, code and other materials | 27 | Report which of the following are publicly available and where they can be found: template data collection forms; data extracted from included studies; data used for all analyses; analytic code; any other materials used in the review. | p. 15 |

Abbreviations: page: p, pages: pp, not applicable: N/A

*No indications of missing data in the review

*From:*  Page MJ, McKenzie JE, Bossuyt PM, Boutron I, Hoffmann TC, Mulrow CD, et al. The PRISMA 2020 statement: an updated guideline for reporting systematic reviews. BMJ 2021;372:n71. doi: 10.1136/bmj.n71

For more information, visit: <http://www.prisma-statement.org/>

**References of the included literature**

1. Abrams HC, Moyer PH, Dyer KS. A model of survival from out-of-hospital cardiac arrest using the Boston EMS arrest registry. Resuscitation. 2011 Aug;82(8):999-1003. doi: 10.1016/j.resuscitation.2011.03.023. Epub 2011 Mar 31. PMID: 21546147.

2. Afzali M, Hesselfeldt R, Steinmetz J, Thomsen AB, Rasmussen LS. A helicopter emergency medical service may allow faster access to highly specialised care. Dan Med J. 2013 Jul;60(7):A4647. PMID: 23809963.

3. Ahmoudi A, Smith M, Jones J. Key survival factor for OHCA patients - Time of cardiac arrest to first CPR

Resuscitation / 2022;175(Supplement 1):S64. Netherlands Elsevier Ireland Ltd 2022 /

4. Al-Dury N, Ravn-Fischer A, Hollenberg J, Israelsson J, Nordberg P, Strömsöe A, et al. Identifying the relative importance of predictors of survival in out of hospital cardiac arrest: a machine learning study. Scand J Trauma Resusc Emerg Med. 2020 Jun 25;28(1):60. doi: 10.1186/s13049-020-00742-9. PMID: 32586339; PMCID: PMC7318370.

5. Alqudah Z, Nehme Z, Williams B, Oteir A, Bernard S, Smith K. Impact of temporal changes in the epidemiology and management of traumatic out-of-hospital cardiac arrest on survival outcomes. Resuscitation. 2021 Jan;158:79-87. doi: 10.1016/j.resuscitation.2020.11.026. Epub 2020 Nov 27. PMID: 33253769.

6. Alumran A, Albinali H, Saadah A, Althumairi A. The Effects of Ambulance Response Time on Survival Following Out-of-Hospital Cardiac Arrest. Open Access Emerg Med. 2020 Dec 1;12:421-426. doi: 10.2147/OAEM.S270837. PMID: 33293876; PMCID: PMC7718983.

7. Aziz A, Muhamad, N. (2020). Outcomes of out-of-hospital cardiac arrest in relation to prehospital care services in Hospital Canselor Tuanku Muhriz: A prospective study. Journal of Emergency Medicine, Trauma and Acute Care. 2020. 10.5339/jemtac.2020.11.

8 Ruiz Azpiazu JI, Fernández Del Valle P, Carmen Escriche M, Royo Embid S, Fernández Barreras C, Azeli Y, et al. Incidence, treatment, and factors associated with survival of out-of-hospital cardiac arrest attended by Spanish emergency services: report from the Out-of-Hospital Spanish Cardiac Arrest Registry for 2022. Emergencias. 2024 Apr;36(2):131-139. Spanish, English. doi: 10.55633/s3me/014.2024. PMID: 38597620.

9. Bagher A, Todorova L, Andersson L, Wingren CJ, Ottoson A, Wangefjord S, et al. Analysis of pre-hospital rescue times on mortality in trauma patients in a Scandinavian urban setting. Trauma. 2017;19(1):28-34. doi:10.1177/1460408616649217

10. Baker PW, Conway J, Cotton C, Ashby DT, Smyth J, Woodman RJ, et al. Defibrillation or cardiopulmonary resuscitation first for patients with out-of-hospital cardiac arrests found by paramedics to be in ventricular fibrillation? A randomised control trial. Resuscitation. 2008 Dec;79(3):424-31. doi: 10.1016/j.resuscitation.2008.07.017. Epub 2008 Nov 4. PMID: 18986748.

11. Berge SD, Berg-Utby C, Skogvoll E. Helicopter transport of sick neonates: a 14-year population-based study. Acta Anaesthesiol Scand. 2005 Aug;49(7):999-1003. doi: 10.1111/j.1399-6576.2005.00712.x. PMID: 16045662.

12. Biewener A, Holch M, Muller U, Veitlinger A, Erfurt C, Zwipp H. Impact of preclinical effort and logistics on letality after severe trauma. Unfallchirurg / 2000;103(2):137-143. Germany Springer Verlag (Tiergartenstrasse 17, Heidelberg D-69121, Germany) 2000

13. Björkman J, Setälä P, Pulkkinen I, Raatiniemi L, Nurmi J. Effect of time intervals in critical care provided by helicopter emergency medical services on 30-day survival after trauma. Injury. 2022 May;53(5):1596-1602. doi: 10.1016/j.injury.2022.01.025. Epub 2022 Jan 15. PMID: 35078619.

14. Björnsson HM, Marelsson S, Magnusson V, Sigurdsson G, Thornorgeirsson G. Endurlífgunartilraunir utan sjúkrahúsa á höfudborgarsvaedinu 1999-2002 [Prehospital cardiac life support in the Reykjavík area 1999-2002]. Laeknabladid. 2006 Sep;92(9):591-7. Icelandic. PMID: 17018972.

15. Blackwell TH, Kline JA, Willis JJ, Hicks GM. Lack of association between prehospital response times and patient outcomes. Prehosp Emerg Care. 2009 Oct-Dec;13(4):444-50. doi: 10.1080/10903120902935363. PMID: 19731155.

16. Blaengsdottir GH, Thorgeirsson G. Advanced cardiac life support outside the hospital in Reykjavik and the surrounding area 1987-1990. Laeknabladid. 1994 Oct;80(8):381-6. Icelandic. PMID: 21593533.

17. Blanchard IE, Doig CJ, Hagel BE, Anton AR, Zygun DA, Kortbeek JB, Powell DG, Williamson TS, Fick GH, Innes GD. Emergency medical services response time and mortality in an urban setting. Prehosp Emerg Care. 2012 Jan-Mar;16(1):142-51. doi: 10.3109/10903127.2011.614046. Epub 2011 Oct 25. PMID: 22026820.

18. Bossers SM, Boer C, Bloemers FW, Van Lieshout EMM, Den Hartog D, et al; BRAIN-PROTECT Collaborators. Epidemiology, Prehospital Characteristics and Outcomes of Severe Traumatic Brain Injury in The Netherlands: The BRAIN-PROTECT Study. Prehosp Emerg Care. 2021 Sep-Oct;25(5):644-655. doi: 10.1080/10903127.2020.1824049. Epub 2020 Nov 3. PMID: 32960672.

19. Brede JR, Kramer-Johansen J, Rehn M. A needs assessment of resuscitative endovascular balloon occlusion of the aorta (REBOA) in non-traumatic out-of-hospital cardiac arrest in Norway. BMC Emerg Med. 2020 Apr 21;20(1):28. doi: 10.1186/s12873-020-00324-z. PMID: 32316924; PMCID: PMC7175537.

20. Brison RJ, Davidson JR, Dreyer JF, Jones G, Maloney J, Munkley DP, et al. Cardiac arrest in Ontario: circumstances, community response, role of prehospital defibrillation and predictors of survival. CMAJ. 1992 Jul 15;147(2):191-9. PMID: 1623465; PMCID: PMC1336161.

21. Brown E, Tohira H, Bailey P, Fatovich D, Pereira G, Finn J. Longer Prehospital Time was not Associated with Mortality in Major Trauma: A Retrospective Cohort Study. Prehosp Emerg Care. 2019 Jul-Aug;23(4):527-537. doi: 10.1080/10903127.2018.1551451. Epub 2019 Jan 24. PMID: 30462550.

22. Bujak K, Nadolny K, Ładny JR, Hudzik B, Zyśko D, Trzeciak P, et al. Epidemiology, management, and survival rate of out-of-hospital cardiac arrest in Upper Silesia, Poland: an Utstein-style report. Postepy Kardiol Interwencyjnej. 2021 Dec;17(4):366-375. doi: 10.5114/aic.2021.111926. Epub 2021 Dec 28. PMID: 35126551; PMCID: PMC8802637.

23. Bürger A, Wnent J, Bohn A, Jantzen T, Brenner S, Lefering R, Seewald S, et al. The Effect of Ambulance Response Time on Survival Following Out-of-Hospital Cardiac Arrest. Dtsch Arztebl Int. 2018 Aug 20;115(33-34):541-548. doi: 10.3238/arztebl.2018.0541. PMID: 30189973; PMCID: PMC6156551.

24. Byrne JP, Mann NC, Dai M, Mason SA, Karanicolas P, Rizoli S, et al. Association Between Emergency Medical Service Response Time and Motor Vehicle Crash Mortality in the United States. JAMA Surg. 2019 Apr 1;154(4):286-293. doi: 10.1001/jamasurg.2018.5097. PMID: 30725080; PMCID: PMC6484802.

25. Cardoso RG, Francischini CF, Ribera JM, Vanzetto R, Fraga GP. Helicopter emergency medical rescue for the traumatized: experience in the metropolitan region of Campinas, Brazil. Rev Col Bras Cir. 2014 Jul-Aug;41(4):236-44. English, Portuguese. doi: 10.1590/0100-69912014004003. PMID: 25295983.

26. Chang I, Lee SC, Shin SD, Song KJ, Ro YS, Park JH, et al. Effects of dispatcher-assisted bystander cardiopulmonary resuscitation on neurological recovery in paediatric patients with out-of-hospital cardiac arrest based on the pre-hospital emergency medical service response time interval. Resuscitation. 2018 Sep;130:49-56. doi: 10.1016/j.resuscitation.2018.06.029. Epub 2018 Jun 28. PMID: 29960075.

27. Chen TT, Ma MH, Chen FJ, Hu FC, Lu YC, Chiang WC, et al. The relationship between survival after out-of-hospital cardiac arrest and process measures for emergency medical service ambulance team performance. Resuscitation. 2015 Dec;97:55-60. doi: 10.1016/j.resuscitation.2015.04.035. Epub 2015 Jun 14. PMID: 26083826.

28. Chesters A, Harris T, Hodgetts TJ, Keefe N. Survival to Discharge After Cardiac Arrest Attended by a Doctor-Paramedic Helicopter Emergency Medical Service: An Utstein-style Multiservice Review of 1085 Activations. J Emerg Med. 2015 Oct;49(4):439-47. doi: 10.1016/j.jemermed.2015.05.001. Epub 2015 Jul 11. PMID: 26168871.

29. Claesson A, Svensson L, Silfverstolpe J, Herlitz J. Characteristics and outcome among patients suffering out-of-hospital cardiac arrest due to drowning. Resuscitation. 2008 Mar;76(3):381-7. doi: 10.1016/j.resuscitation.2007.09.003. Epub 2007 Nov 7. PMID: 17997210.

30. Deasy C, Bray J, Smith K, Harriss L, Morrison C, Bernard S, et al. Traumatic out-of-hospital cardiac arrests in Melbourne, Australia. Resuscitation. 2012 Apr;83(4):465-70. doi: 10.1016/j.resuscitation.2011.09.025. Epub 2011 Oct 10. PMID: 21996019.

31. de Graaf C, Beesems SG, Koster RW. Time of on-scene resuscitation in out of-hospital cardiac arrest patients transported without return of spontaneous circulation. Resuscitation. 2019 May;138:235-242. doi: 10.1016/j.resuscitation.2019.03.030. Epub 2019 Mar 27. Erratum in: Resuscitation. 2019 Jul;140:223. PMID: 30928502.

32. Dicker B, Davey P, Smith T, Beck B. Incidence and outcomes of out-of-hospital cardiac arrest: A New Zealand perspective. Emerg Med Australas. 2018 Oct;30(5):662-671. doi: 10.1111/1742-6723.12966. Epub 2018 Mar 23. PMID: 29569842.

33. Dinh M, Singh H, Deans C, Pople G, Bendall J, Sarrami P. Prehospital times and outcomes of patients transported using an ambulance trauma transport protocol: A data linkage analysis from New South Wales Australia. Injury. 2023 Oct;54(10):110988. doi: 10.1016/j.injury.2023.110988. Epub 2023 Aug 7. PMID: 37574381.

34. Dyson K, Morgans A, Bray J, Matthews B, Smith K. Drowning related out-of-hospital cardiac arrests: characteristics and outcomes. Resuscitation. 2013 Aug;84(8):1114-8. doi: 10.1016/j.resuscitation.2013.01.020. Epub 2013 Jan 29. PMID: 23370162.

35. Einarsson O, Jakobsson F, Sigurdsson G. Advanced cardiac life support in the prehospital setting: the Reykjavik experience. J Intern Med. 1989 Feb;225(2):129-35. doi: 10.1111/j.1365-2796.1989.tb00052.x. PMID: 2921594.

36. Eisenburger P, Czappek G, Sterz F, Vergeiner G, Losert H, Holzer M, et al. Cardiac arrest patients in an alpine area during a six year period. Resuscitation. 2001 Oct;51(1):39-46. doi: 10.1016/s0300-9572(01)00387-2. PMID: 11719172.

37. Eng Hock Ong M, Chan YH, Anantharaman V, Lau ST, Lim SH, Seldrup J. Cardiac arrest and resuscitation epidemiology in Singapore (CARE I study). Prehosp Emerg Care. 2003 Oct-Dec;7(4):427-33. doi: 10.1080/312703002120. PMID: 14582091.

38. Fake AL, Swain AH, Larsen PD. Survival from out-of-hospital cardiac arrest in Wellington in relation to socioeconomic status and arrest location. N Z Med J. 2013 Jun 14;126(1376):28-37. PMID: 23822959.

39. Finney O, Stagg H. Rural versus urban out-of-hospital cardiac arrest response, treatment and outcomes in the North East of England from 2018 to 2019. Br Paramed J. 2023 Sep 1;8(2):29-37. doi: 10.29045/14784726.2023.9.8.2.29. PMID: 37674914; PMCID: PMC10477825.

40. Fothergill RT, Smith AL, Wrigley F, Perkins GD. Out-of-Hospital Cardiac Arrest in London during the COVID-19 pandemic. Resusc Plus. 2021 Mar;5:100066. doi: 10.1016/j.resplu.2020.100066. Epub 2020 Dec 21. PMID: 33521706; PMCID: PMC7833716.

41. Fraga-Sastrías JM, Asensio-Lafuente E, Martínez R, Bárcenas IA, Prieto-Sagredo J, Castillo L, et al. Out-of-hospital cardiac arrest: first documented experience in a Mexican urban setting. Prehosp Disaster Med. 2009 Mar-Apr;24(2):121-5. doi: 10.1017/s1049023x0000666x. PMID: 19591305.

42. Gnesin F, Møller AL, Mills EHA, Zylyftari N, Jensen B, Bøggild H, et al. Rapid dispatch for out-of-hospital cardiac arrest is associated with improved survival. Resuscitation. 2021 Jun;163:176-183. doi: 10.1016/j.resuscitation.2021.03.015. Epub 2021 Mar 26. PMID: 33775800.

43. Goh CE, Mooney SJ, Siscovick DS, Lemaitre RN, Hurvitz P, Sotoodehnia N, et al. Medical facilities in the neighborhood and incidence of sudden cardiac arrest. Resuscitation. 2018 Sep;130:118-123. doi: 10.1016/j.resuscitation.2018.07.005. Epub 2018 Jul 6. PMID: 30057353; PMCID: PMC6467836.

44. Goh ES, Liang B, Fook-Chong S, Shahidah N, Soon SS, et al. Effect of location of out-of-hospital cardiac arrest on survival outcomes. Ann Acad Med Singap. 2013 Sep;42(9):437-44. PMID: 24162318.

45. Gregers MCT, Møller SG, Kjoelbye JS, Jakobsen LK, Grabmayr AJ, Kragh AR, et al. Association of Degree of Urbanization and Survival in Out-of-Hospital Cardiac Arrest. J Am Heart Assoc. 2023 May 16;12(10):e8322. doi: 10.1161/JAHA.122.028449. Epub 2023 May 9. PMID: 37158087; PMCID: PMC10227318.

46. Grmec S, Strnad M, Podgorsek D. Comparison of the characteristics and outcome among patients suffering from out-of-hospital primary cardiac arrest and drowning victims in cardiac arrest. Int J Emerg Med. 2009 Apr;2(1):7-12. doi: 10.1007/s12245-009-0084-0. Epub 2009 Feb 14. PMID: 19390911; PMCID: PMC2672980.

47. Gunaga S, Nippert J, Kerr M, Young B, Muller G, Charping A, et al. Comparing Response Times, Intensity of Care and Outcomes between Private versus Municipal Emergency Medical Services Systems Crit Care. 2016 Apr 20;20(Suppl 2):94. doi: 10.1186/s13054-016-1208-6

48. Han K, Seoung MP. Association Between the Arrival Time of Paramedics and the Neurological Outcome at Discharge of Cardiac Arrest Patients. 30 Oct 2022.https://doi.org/10.1161/circ.146.suppl_1.14500Circulation. 2022;146:A1450

49. Hayes C, Murphy BD, Lynch M. Out of hospital cardiac arrest continues to carry a high mortality - Room to improve time to defibrillation - The Dublin West Experience

Fundamental and Clinical Pharmacology / 2010;24(1):3 Blackwell Publishing Ltd 2010 /

50. Henry K, Murphy A, Willis D, Cusack S, Bury G, O'Sullivan I, et al. Out-of-hospital cardiac arrest in Cork, Ireland. Emerg Med J. 2013 Jun;30(6):496-500. doi: 10.1136/emermed-2011-200888. Epub 2012 Jun 15. PMID: 22707474.

51. Herlitz J, Svensson L, Engdahl J, Angquist KA, Silfverstolpe J, Holmberg S. Association between interval between call for ambulance and return of spontaneous circulation and survival in out-of-hospital cardiac arrest. Resuscitation. 2006 Oct;71(1):40-6. doi: 10.1016/j.resuscitation.2006.03.006. Epub 2006 Aug 30. PMID: 16945468.

52. Herlitz J, Svensson L, Engdahl J, Silfverstolpe J. Characteristics and outcome in out-of-hospital cardiac arrest when patients are found in a non-shockable rhythm. Resuscitation. 2008 Jan;76(1):31-6. doi: 10.1016/j.resuscitation.2007.06.027. Epub 2007 Aug 20. PMID: 17709164.

53. Hillis M, Sinclair D, Butler G, Cain E. Prehospital cardiac arrest survival and neurologic recovery. J Emerg Med. 1993 May-Jun;11(3):245-52. doi: 10.1016/0736-4679(93)90041-5. PMID: 8340577.

54. Holmén J, Herlitz J, Ricksten SE, Strömsöe A, Hagberg E, Axelsson C, et al. Shortening Ambulance Response Time Increases Survival in Out-of-Hospital Cardiac Arrest. J Am Heart Assoc. 2020 Nov 3;9(21):e017048. doi: 10.1161/JAHA.120.017048. Epub 2020 Oct 27. PMID: 33107394; PMCID: PMC7763420.

55. Huabbangyang T, Soion T, Promdee A, Kasemchai Nguanjinda RN, Assanai Chamchan RN, Ratree Chaisorn PMD, et al. Factors Associated with Successful Resuscitation during Out-of-Hospital Cardiac Arrest Performed By Surgico Medical Ambulance and Rescue Team (S.M.A.R.T), Division of Emergency Medical Service and Disaster, Faculty of Medicine Vajira Hospital, Navamindradhiraj University. J Med Assoc Thai 2021;104:1-9.doi.org/10.35755/jmedassocthai.2021.09.12759

56. Hubert H, Escutnaire J, Michelet P, Babykina E, El Khoury C, Tazarourte K, et al. Can we identify termination of resuscitation criteria in cardiac arrest due to drowning: results from the French national out-of-hospital cardiac arrest registry. J Eval Clin Pract. 2016 Dec;22(6):924-931. doi: 10.1111/jep.12562. Epub 2016 Jun 13. PMID: 27292052.

57. Jennings PA, Cameron P, Walker T, Bernard S, Smith K. Out-of-hospital cardiac arrest in Victoria: rural and urban outcomes. Med J Aust. 2006 Aug 7;185(3):135-9. doi: 10.5694/j.1326-5377.2006.tb00498.x. PMID: 16893352.

58. Jeong H, Moon H, Lee J, Lee D, Choi J, Jung Y. The effect of ambulance response time in the outcomes of patients with out-of-hospital cardiac arrest. Resuscitation / 2017;118(Supplement 1):e35. Netherlands Elsevier Ireland Ltd 2017 /

59. Kennedy C, Alqudah Z, Stub D, Anderson D, Nehme Z. The effect of the COVID-19 pandemic on the incidence and survival outcomes of EMS-witnessed out-of-hospital cardiac arrest. Resuscitation. 2023 Jun;187:109770. doi: 10.1016/j.resuscitation.2023.109770. Epub 2023 Mar 17. PMID: 36933880; PMCID: PMC10019917.

60. Kentsch M, Schlichting H, Mathes N, Rodemerk U, Ittel TH. Out-of-hospital cardiac arrest in north-east Germany: increased resuscitation efforts and improved survival. Resuscitation. 2000 Feb;43(3):177-83. doi: 10.1016/s0300-9572(99)00138-0. PMID: 10711486.

61. Kitano S, Fujimoto K, Suzuki K, Harada S, Narikawa K, Yamada M, et al. Evaluation of outcomes after EMS-witnessed traumatic out-of-hospital cardiac arrest caused by traffic collisions. Resuscitation. 2022 Feb;171:64-70. doi: 10.1016/j.resuscitation.2021.12.023. Epub 2021 Dec 24. PMID: 34958879.

62. Kłosiewicz T, Skitek-Adamczak I, Zieliński M. Emergency medical system response time does not affect incidence of return of spontaneous circulation after prehospital resuscitation in one million central European agglomeration residents. Kardiol Pol. 2017;75(3):240-246. doi: 10.5603/KP.a2016.0181. Epub 2016 Dec 20. PMID: 27995600.

63. Lee DW, Moon HJ, Heo NH; KoCARC. Association between ambulance response time and neurologic outcome in patients with cardiac arrest. Am J Emerg Med. 2019 Nov;37(11):1999-2003. doi: 10.1016/j.ajem.2019.02.021. Epub 2019 Feb 16. PMID: 30795948.

64. Lee IH, How CK, Lu WH, Tzeng YM, Chen YJ, Chern CH, et al. Improved survival outcome with continuous chest compressions with ventilation compared to 5:1 compressions-to-ventilations mechanical cardiopulmonary resuscitation in out-of-hospital cardiac arrest. J Chin Med Assoc. 2013 Mar;76(3):158-63. doi: 10.1016/j.jcma.2013.01.001. Epub 2013 Feb 5. PMID: 23497969.

65. Leung LP, Wong TW, Tong HK, Lo CB, Kan PG. Out-of-hospital cardiac arrest in Hong Kong. Prehosp Emerg Care. 2001 Jul-Sep;5(3):308-11. doi: 10.1080/10903120190939887. PMID: 11446552.

66. Lim SL, Smith K, Dyson K, Chan SP, Earnest A, Nair R, et al. Incidence and Outcomes of Out-of-Hospital Cardiac Arrest in Singapore and Victoria: A Collaborative Study. J Am Heart Assoc. 2020 Nov 3;9(21):e015981. doi: 10.1161/JAHA.119.015981. Epub 2020 Oct 23. PMID: 33094661; PMCID: PMC7763419.

67. Lin YN, Chang SS, Wang LM, Ueng KC, Tsai CF, Phan CS, et al. Low incidence of ventricular tachycardia/fibrillation as the first presenting rhythm in patients with out-of-hospital cardiac arrest: Evidence from the taichung sudden unexpected death registry (THUNDER) in East Asia. Circulation / 2014;130(SUPPL. 2): Lippincott Williams and Wilkins 2014 /

68. Little CD, Kotecha T, Candilio L, Jabbour RJ, Collins GB, Ahmed A, et al. COVID-19 pandemic and STEMI: pathway activation and outcomes from the pan-London heart attack group. Open Heart. 2020 Oct;7(2):e001432. doi: 10.1136/openhrt-2020-001432. PMID: 33106441; PMCID: PMC7592245.

69. Liu CH, Tsai MJ, Hsu CF, Tsai CH, Su YS, Cai DC. The Influence of the COVID-19 Pandemic on Emergency Medical Services to Out-of-Hospital Cardiac Arrests in a Low-Incidence Urban City: An Observational Epidemiological Analysis. Int J Environ Res Public Health. 2023 Feb 3;20(3):2713. doi: 10.3390/ijerph20032713. PMID: 36768079; PMCID: PMC9915115.

70. Lyon RM, Nelson MJ. Helicopter emergency medical services (HEMS) response to out-of-hospital cardiac arrest. Scand J Trauma Resusc Emerg Med. 2013 Jan 7;21:1. doi: 10.1186/1757-7241-21-1. PMID: 23294807; PMCID: PMC3570349.

71. Margey R, Browne L, Murphy E, O'Reilly M, Mahon N, Blake G, et al. The Dublin cardiac arrest registry: temporal improvement in survival from out-of-hospital cardiac arrest reflects improved pre-hospital emergency care. Europace. 2011 Aug;13(8):1157-65. doi: 10.1093/europace/eur092. Epub 2011 Apr 6. PMID: 21474456.

72. Mathiesen WT, Bjørshol CA, Kvaløy JT, Søreide E. Effects of modifiable prehospital factors on survival after out-of-hospital cardiac arrest in rural versus urban areas. Crit Care. 2018 Apr 18;22(1):99. doi: 10.1186/s13054-018-2017-x. PMID: 29669574; PMCID: PMC5907488.

73. Mayer JD. Paramedic response time and survival from cardiac arrest. Soc Sci Med Med Geogr. 1979 Dec;13D(4):267-71. doi: 10.1016/0160-8002(79)90049-2. PMID: 524134.

74. Meyer AD, Bernard S, Smith KL, McNeil JJ, Cameron PA. Asystolic cardiac arrest in Melbourne, Australia. Emerg Med (Fremantle). 2001 Jun;13(2):186-9. doi: 10.1046/j.1442-2026.2001.00208.x. PMID: 11482856.

75. Mikiewicz M, Polok K, Szczeklik W, Górka A, Kosiński S. Sudden Cardiac Arrests in the Polish Tatra Mountains: A Retrospective Study. Wilderness Environ Med. 2023 Jun;34(2):128-134. doi: 10.1016/j.wem.2022.11.005. Epub 2023 Jan 28. PMID: 36710127.

76. Mikkelsen S, Lossius HM, Toft P, Lassen AT. Characteristics and prognoses of patients treated by an anaesthesiologist-manned prehospital emergency care unit. A retrospective cohort study. BMJ Open. 2017 Feb 22;7(2):e014383. doi: 10.1136/bmjopen-2016-014383. PMID: 28232468; PMCID: PMC5337743.

77. Mills AAM, Mills EHA, Blomberg SNF, Christensen HC, Møller AL, et al. Ambulance response times and 30-day mortality: a Copenhagen (Denmark) registry study. Eur J Emerg Med. 2024 Feb 1;31(1):59-67. doi: 10.1097/MEJ.0000000000001094. Epub 2023 Oct 2. PMID: 37788140.

78. Mills EHA, Aasbjerg K MD, PhD, Hansen SM, Ringgren KB MB, Dahl M MD, PhD, Rasmussen BS, et al. Prehospital time and mortality in patients requiring a highest priority emergency medical response: a Danish registry-based cohort study. BMJ Open. 2019 Nov 21;9(11):e023049. doi: 10.1136/bmjopen-2018-023049. PMID: 31753864; PMCID: PMC6886969.

79. Mogensen BA, Bjornsson HM, Thorgeirsson G, Haraldsson GE, Mogensen B. Árangur endurlífgunartilrauna utan spítala a Reykjavíkursvæðinu árin 2004-2007 [Results of pre-hospital cardiac resuscitation in the Reykjavik area 2004-2007]. Laeknabladid. 2015 Mar;101(3):137-41. Icelandic. doi: 10.17992/lbl.2015.03.18. PMID: 25735673.

80. Morrisey MA, Ohsfeldt RL, Johnson V, Treat R. Trauma patients: an analysis of rural ambulance trip reports. J Trauma. 1996 Oct;41(4):741-6. doi: 10.1097/00005373-199610000-00025. PMID: 8858039.

81. Nadolny K, Bujak K, Obremska M, Zysko D, Sterlinski M, Szarpak L, et al. Glasgow Coma Scale score of more than four on admission predicts in-hospital survival in patients after out-of-hospital cardiac arrest. Am J Emerg Med. 2021 Apr;42:90-94. doi: 10.1016/j.ajem.2021.01.018. Epub 2021 Jan 15. PMID: 33497899.

82. Naroo G, Sakaf O, Hamid A, Yadgir T. Early resuscitation for better outcome, time is muscle

Circulation / 2012;125(19):e802. Lippincott Williams and Wilkins 2012 /

83. Navab E, Esmaeili M, Poorkhorshidi N, Salimi R, Khazaei A, Moghimbeigi A. Predictors of Out of Hospital Cardiac Arrest Outcomes in Pre-Hospital Settings; a Retrospective Cross-sectional Study. Arch Acad Emerg Med. 2019 Jul 10;7(1):36. PMID: 31555766; PMCID: PMC6732204.

84. Nichol G, Cobb LA, Yin L, Maynard C, Olsufka M, Larsen J, et al. Briefer activation time is associated with better outcomes after out-of-hospital cardiac arrest. Resuscitation. 2016 Oct;107:139-44. doi: 10.1016/j.resuscitation.2016.06.040. Epub 2016 Jul 21. PMID: 27452490.

85. Nielsen VML, Bruun NH, Søvsø MB, Kløjgård TA, Lossius HM, Bender L, et al. Pediatric Emergencies in Helicopter Emergency Medical Services: A National Population-Based Cohort Study From Denmark. Ann Emerg Med. 2022 Aug;80(2):143-153. doi: 10.1016/j.annemergmed.2022.03.024. Epub 2022 May 5. PMID: 35527122.

86. O'Keeffe C, Nicholl J, Turner J, Goodacre S. Role of ambulance response times in the survival of patients with out-of-hospital cardiac arrest. Emerg Med J. 2011 Aug;28(8):703-6. doi: 10.1136/emj.2009.086363. Epub 2010 Aug 25. PMID: 20798090.

87. Park GJ, Song KJ, Shin SD, Lee KW, Ahn KO, Lee EJ, et al. Timely bystander CPR improves outcomes despite longer EMS times. Am J Emerg Med. 2017 Aug;35(8):1049-1055. doi: 10.1016/j.ajem.2017.02.033. Epub 2017 Feb 16. PMID: 28237384.

88. Pell JP, Sirel JM, Marsden AK, Ford I, Cobbe SM. Effect of reducing ambulance response times on deaths from out of hospital cardiac arrest: cohort study. BMJ. 2001 Jun 9;322(7299):1385-8. doi: 10.1136/bmj.322.7299.1385. PMID: 11397740; PMCID: PMC32251.

89. Pons PT, Haukoos JS, Bludworth W, Cribley T, Pons KA, Markovchick VJ. Paramedic response time: does it affect patient survival? Acad Emerg Med. 2005 Jul;12(7):594-600. doi: 10.1197/j.aem.2005.02.013. PMID: 15995089.

90. Pons PT, Markovchick VJ. Eight minutes or less: does the ambulance response time guideline impact trauma patient outcome? J Emerg Med. 2002 Jul;23(1):43-8. doi: 10.1016/s0736-4679(02)00460-2. PMID: 12217471.

91. Puolakka T, Salo A, Varpula M, Nurmi J, Skrifvars MB, Wilkman E, et al. Hospital-administered ECPR for out-of-hospital cardiac arrest: an observational cohort study. Emerg Med J. 2023 Nov;40(11):754-760. doi: 10.1136/emermed-2023-213292. Epub 2023 Sep 12. PMID: 37699713.

92. Do HQ, Nielsen SL, Rasmussen LS. Response interval is important for survival until admission after prehospital cardiac arrest. Dan Med Bull. 2010 Dec;57(12):A4203. PMID: 21122459.

93. Rajan S, Wissenberg M, Folke F, Hansen SM, Gerds TA, Kragholm K, et al. Association of Bystander Cardiopulmonary Resuscitation and Survival According to Ambulance Response Times After Out-of-Hospital Cardiac Arrest. Circulation. 2016 Dec 20;134(25):2095-2104. doi: 10.1161/CIRCULATIONAHA.116.024400. Epub 2016 Nov 22. PMID: 27881566.

94. Renkiewicz GK, Hubble MW, Wesley DR, Dorian PA, Losh MJ, Swain R, et al. Probability of a shockable presenting rhythm as a function of EMS response time. Prehosp Emerg Care. 2014 Apr-Jun;18(2):224-30. doi: 10.3109/10903127.2013.851308. Epub 2014 Jan 8. PMID: 24400944.

95. Schinnerl A, Kroesen G, Baubin M, Benzer H. Ergebnis der präklinischen kardiopulmonalen Reanimation in den ersten Betriebsjahren eines NAW-Systems [The results of prehospital cardiopulmonary resuscitation in the initial years of a mobile emergency care system]. Anaesthesist. 1990 Oct;39(10):469-74. German. PMID: 2278364.

96. Semensato G, Zimerman L, Rohde LE. Initial evaluation of the Mobile Emergency Medical Services in the city of Porto Alegre, Brazil. Arq Bras Cardiol. 2011 Mar;96(3):196-204. English, Portuguese, Spanish. doi: 10.1590/s0066-782x2011005000019. Epub 2011 Feb 25. PMID: 21359486.

97. Shah AS, Bhopal R, Gadd S, Donohoe R. Out-of-hospital cardiac arrest in South Asian and white populations in London: database evaluation of characteristics and outcome. Heart. 2010 Jan;96(1):27-9. doi: 10.1136/hrt.2009.170183. Epub 2009 Sep 10. PMID: 19744967.

98. Shepherd MV, Trethewy CE, Kennedy J, Davis L. Helicopter use in rural trauma. Emerg Med Australas. 2008 Dec;20(6):494-9. doi: 10.1111/j.1742-6723.2008.01135.x. PMID: 19125828.

99. Sigurðsson G, Thornorgeirsson G. [Advanced cardiac life support in the prehospital setting in the Reykjavik area 1991-1996.]. Laeknabladid. 2000;86(10):669-73. Icelandic. PMID: 17018957.

100. Sipria A, Kirsimagi U, Popov A, Veber A. Out-of-hospital resuscitation from sudden cardiac arrest in Estonia 1999-2013. Eesti Arst / 2016;95(7):428-436. Estonia OU Celsius Healthcare (E-mail: celsius@celsius.ee) 2016 /

101. Spaite DW, Bobrow BJ, Vadeboncoeur TF, Chikani V, Clark L, Mullins T, et al. The impact of prehospital transport interval on survival in out-of-hospital cardiac arrest: implications for regionalization of post-resuscitation care. Resuscitation. 2008 Oct;79(1):61-6. doi: 10.1016/j.resuscitation.2008.05.006. Epub 2008 Jul 9. PMID: 18617315.

102. Stoeckl M, Sterz F, Weiser C. Dispatch versus ambulance response interval and survival after out-of -hospital cardiac arrest. Resuscitation / 2010;81(2 SUPPL. 1):S87. Elsevier Ireland Ltd 2010

103. Strömsöe A, Svensson L, Axelsson ÅB, Claesson A, Göransson KE, Nordberg P, et al. Improved outcome in Sweden after out-of-hospital cardiac arrest and possible association with improvements in every link in the chain of survival. Eur Heart J. 2015 Apr 7;36(14):863-71. doi: 10.1093/eurheartj/ehu240. Epub 2014 Jun 17. PMID: 25205528.

104. Strömsöe A, Svensson L, Claesson A, Lindkvist J, Lundström A, Herlitz J. Association between population density and reported incidence, characteristics and outcome after out-of-hospital cardiac arrest in Sweden. Resuscitation. 2011 Oct;82(10):1307-13. doi: 10.1016/j.resuscitation.2011.04.025. Epub 2011 May 14. PMID: 21628082.

105. Sugiyama J, Inoue S, Inada M, Miyazaki Y, Nakanishi N, Fujinami Y, et al. Impact of the coronavirus disease 2019 (COVID-19) pandemic on the operational efficiency of emergency medical services and its association with out-of-hospital cardiac arrest survival rates: A population-based cohort study in Kobe, Japan. Acute Med Surg. 2023 Jun 25;10(1):e00865. doi: 10.1002/ams2.865. PMID: 37366417; PMCID: PMC10290879.

106. Syväoja S, Salo A, Uusaro A, Jäntti H, Kuisma M. Witnessed out-of-hospital cardiac arrest- effects of emergency dispatch recognition. Acta Anaesthesiol Scand. 2018 Apr;62(4):558-567. doi: 10.1111/aas.13051. Epub 2017 Dec 19. PMID: 29266165.

107. Ter Avest E, Griggs J, Prentice C, Jeyanathan J, Lyon RM. Out-of-hospital cardiac arrest following trauma: What does a helicopter emergency medical service offer? Resuscitation. 2019 Feb;135:73-79. doi: 10.1016/j.resuscitation.2018.12.019. Epub 2018 Dec 28. PMID: 30597132.

108. Thompson L, Hill M, Davies C, Shaw G, Kiernan MD. Identifying pre-hospital factors associated with outcome for major trauma patients in a regional trauma network: an exploratory study. Scand J Trauma Resusc Emerg Med. 2017 Aug 23;25(1):83. doi: 10.1186/s13049-017-0419-4. PMID: 28835283; PMCID: PMC5569481.

109. Trenkler S, Kilianova A, Paulikova M, Karas J. Resuscitation of out-of-hospital cardiac arrest in Falck Zachranna - Start of Slovak registry. Resuscitation / 2012;83(SUPPL. 1):e43. Elsevier Ireland Ltd 2012 /

110. Tsai SL, Chaou CH, Huang CH, Tzeng IS, Kuo CW, Weng YM, et al. Features of hospital and emergency medical service in out-of-hospital cardiac arrest patients with shockable rhythm. Am J Emerg Med. 2017 Sep;35(9):1222-1227. doi: 10.1016/j.ajem.2017.03.032. Epub 2017 Mar 18. PMID: 28341188.

111. Wai AKC, Cameron P, Cheung CK, Mak P, Rainer TH. Out-of-hospital cardiac arrest in a teaching hospital in Hong Kong: Descriptive study using the Utstein style. Hong Kong Journal of Emergency Medicine / 2005;12(3):148-155. Australia Medcom Limited 2005.

112. Weinlich M, Martus P, Blau MB, Wyen H, Walcher F, Piatek S, Schüttrumpf JP. Competitive advantage gained from the use of helicopter emergency medical services (HEMS) for trauma patients: Evaluation of 1724 patients. Injury. 2019 May;50(5):1028-1035. doi: 10.1016/j.injury.2018.12.018. Epub 2018 Dec 16. PMID: 30591228.

113. Wik L, Hansen TB, Fylling F, Steen T, Vaagenes P, Auestad BH, et al. Delaying defibrillation to give basic cardiopulmonary resuscitation to patients with out-of-hospital ventricular fibrillation: a randomized trial. JAMA. 2003 Mar 19;289(11):1389-95. doi: 10.1001/jama.289.11.1389. PMID: 12636461.

114. Wissa J, Schultz BV, Wilson D, Rashford S, Bosley E, Doan TN. Time to amiodarone administration and survival outcomes in refractory ventricular fibrillation. Emerg Med Australas. 2021 Dec;33(6):1088-1094. doi: 10.1111/1742-6723.13841. Epub 2021 Aug 11. PMID: 34382325.

115. Zheng J, Lv C, Zheng W, Zhang G, Tan H, Ma Y, et al. Incidence, process of care, and outcomes of out-of-hospital cardiac arrest in China: a prospective study of the BASIC-OHCA registry. Lancet Public Health. 2023 Dec;8(12):e923-e932. doi: 10.1016/S2468-2667(23)00173-1. Epub 2023 Sep 16. PMID: 37722403.
