# Supplementary material for: Association of ambulance and helicopter response times with patient survival: A systematic literature review and meta-analysis
Source: PLoS One. 2025 Nov 17;20(11):e0335665. doi: 10.1371/journal.pone.0335665 (PMC12622838; doi:10.1371/journal.pone.0335665)
Supplement: S4 Table — (DOCX) [file pone.0335665.s005.docx]

**REPOSITORY TABLE 4. Data extracted from the primary research sources**

| **Reference** | **Patient centered outcome 1** | **Patient centered**  **outcome 2** | **EMS centered measure 1** | **EMS centered measuree 2** | **Study period** | **Data and quality appraisal instruments** | **Date of extraction** | **Extractors** | **Eligible** |
| --- | --- | --- | --- | --- | --- | --- | --- | --- | --- |
| **Abrams 2011^1^** | Survival to discharge | ROSC | Response time |  | 01.01.04-31.12.07 | X | 06.02.24 | MSN, PMH | Y |
| **Afzali 2013^2^** | 30-day survival | ICU admission | Response time |  | 01.05.10-30.04.11 | X | 06.02.24 | MSN, PMH | Y |
| **Ahmoudi 2022^3^** | Survival to discharge | ROSC | Response time | Collapse-to-CPR time | N/R | X | 06.02.24 | MSN, PMH | Y |
| **Al-Dury 2020^4^** | 30-day survival |  | Response time | Collapse-to-CPR time | 01.01.08-31.12.16 | X | 06.02.24 | MSN, PMH | Y |
| **Alqudah 2021^5^** | Survival to discharge | GOSE | Response time |  | 01.01.01-31.12.18 | X | 06.02.24 | MSN, PMH | Y |
| **Alumran 2020^6^** | Survival to discharge |  | Response time |  | 01.01.17-31.10.18 | X | 06.02.24 | MSN, PMH | Y |
| **Aziz 2020^7^** | Survival to discharge | CPC | Response time |  | 01.09.18-31.05.19 | X | 06.02.24 | MSN, PMH | Y |
| **Azpiazu 2024^8^**  **Bagher 2017^9^** | Survival to discharge  90-day survival | NISS | Response time  Response time | On-scene time | 01.01.22-31.12.22  01.01.11-31.12.13 | X | 30.10.24  06.02.24 | MSN, PMH  MSN, PMH | Y |
| **Baker 2008^10^** | Survival to discharge | CPC | Response time | 3 min. CPR first vs. defib. | 01.07.05-31.07.07 | X | 06.02.24 | MSN, PMH | Y |
| **Berge 2005^11^** | 24H-survival | 1-year survival | Response time | Total transport time | 01.01.88-31.12.01 | X | 06.02.24 | MSN, PMH | Y |
| **Biewener 2000^12^** | 24H-survival | 7D-survival | Response time |  | 01.01.93-31.12.94 | X | 07.02.24 | MSN, PMH | Y |
| **Bjorkmann 2022^13^** | 30-day survival |  | Response time | On-scene time | 01.01.12-31.12.18 | X | 07.02.24 | MSN, PMH | Y |
| **Bjornsson 2006^14^** | Survival to discharge |  | Response time |  | 01.01.99-31.12.02 | X | 07.02.24 | MSN, PMH | Y |
| **Blackwell 2009^15^** | Survival to discharge |  | Response time |  | 01.01.04-31.12.04 | X | 07.02.24 | MSN, PMH | Y |
| **Blaengsdottir 1994^16^** | Survival to discharge |  | Response time |  | 01.01.89-31.12.90 | X | 07.02.24 | MSN, PMH | Y |
| **Blanchard 2012^17^** | Survival to discharge |  | Response time | Transport interval | 01.01.06-31.12.06 | X | 07.02.24 | MSN, PMH | Y |
| **Bossers 2021^18^** | 30-day survival | GOS | Response time |  | 01.02.12-31.12.17 | X | 07.02.24 | MSN, PMH | Y |
| **Brede 2020^19^** | 30-day survival | ROSC | Response time | CPR duration | 01.01.16-31.12.18 | X | 07.02.24 | MSN, PMH | Y |
| **Brison 1992^20^** | Survival to discharge | Functional outcome* | Response time |  | 01.03.86-31.07.89 | X | 07.02.24 | MSN, PMH | Y |
| **Brown 2019^21^** | 30-day survival | LOS | Response time | On-scene time | 01.01.13-31.12.16 | X | 07.02.24 | MSN, PMH | Y |
| **Bujak 2021^22^** | Survival to discharge | LOS | Response time | Defibrillation time | 01.01.18-31.12.18 | X | 07.02.24 | MSN, PMH | Y |
| **Burger 2018^23^** | Survival to discharge | ROSC | Response time |  | 01.01.16-31.12.16 | X | 07.02.24 | MSN, PMH | Y |
| **Byrne 2019^24^** | 30-day survival |  | Response time | On-scene time | 01.01.13-31.12.15 | X | 07.02.24 | MSN, PMH | Y |
| **Cardoso 2014^25^** | 30-day survival | 24H-survival | Response time | On-scene time | 01.01.10-31.12.12 | X | 08.02.24 | MSN, PMH | Y |
| **Chang 2018^26^** | Survival to discharge | CPC | Response time |  | 01.01.12-31.12.16 | X | 08.02.24 | MSN, PMH | Y |
| **Chen 2015^27^** | Survival to discharge |  | Response time |  | 01.01.06-31.12.09 | X | 08.02.24 | MSN, PMH | Y |
| **Chesters 2015^28^** | Survival to discharge | CPC | Call-to-arrival time* |  | 10.12.10-30.06.13 | X | 08.02.24 | MSN, PMH | Y |
| **Claesson 2008^29^** | 30-day survival |  | Response time |  | 01.01.90-31.12.05 | X | 08.02.24 | MSN, PMH | Y |
| **deGraaf 2019^30^** | 30-day survival |  | Response time | On-scene time | 01.01.12-31.12.16 | X | 08.02.24 | MSN, PMH | Y |
| **Deasy 2012^31^** | Survival to discharge |  | Response time |  | 01.01.00-31.12.09 | X | 08.02.24 | MSN, PMH | Y |
| **Dicker 2018^32^**  **Dinh 2023^33^** | 30-day survival  30-day survival |  | Response time  Response time |  | 01.10.13-30.09.15  01.01.19-31.12.20 | X | 08.02.24  30.10.24 | MSN, PMH  MSN, PMH | Y  y |
| **Dyson 2013^34^** | Survival to discharge |  | Response time | Presenting rhythm | 01.10.99-31.12.11 | X | 08.02.24 | MSN, PMH | Y |
| **Einarsson 1989^35^** | Survival to discharge |  | Response time |  | 01.01.82-31.12.86 | X | 08.02.24 | MSN, PMH | Y |
| **Eisenburger 2001^36^** | Survival to discharge | 24H-survival | Response time |  | 01.01.91-31.01.98 | X | 08.02.24 | MSN, PMH | Y |
| **Ong 2003^37^** | Survival to discharge |  | Response time | Collapse-to-call time | 01.10.01-30.04.02 | X | 08.02.24 | SMN, PMH | Y |
| **Fake 2013^38^** | Survival to discharge | ROSC | Response time |  | 01.07.07-30.06.10 | X | 09,02.24 | MSN, PMH | Y |
| **Finney 2023^39^** | ROSC |  | Response time | Presenting rhythm | 01.04.18-31.03.19 | X | 09.02.24 | MSN, PMH | Y |
| **Fothergill 2021^40^** | 30-day survival | ROSC | Response time | On-scene time | 01.03.19-30.04.20 | X | 09.02.24 | MSN, PMH | Y |
| **Fraga-Sastrias 2009^41^** | Survival to discharge | ROSC | Response time | Collapse-to-call time | 01.06.06-31.05.07 | X | 09.02.24 | MSN, PMH | Y |
| **Gnesin 2021^42^** | 30-day survival | ROSC | Response time | Time to dispatch | 01.01.16-31.12.18 | X | 09.02.24 | MSN, PMH | Y |
| **Goh 2018^43^** | Survival to discharge |  | Response time |  | 01.01.90-31.12.10 | X | 09.02.24 | MSN, PMH | Y |
| **Goh 2013^44^** | Survival to discharge |  | Response time | Presenting rhythm | 01.10.01-30.09.04 | X | 09.02.24 | MSN, PMH | Y |
| **Gregers 2021^45^** | 30-day survival | ROSC | Response time |  | 01.01.16-31.12.19 | X | 09.02.24 | MSN, PMH | Y |
| **Grmec 2009^46^** | Survival to discharge | ROSC | Response time |  | 01.02.98-31.01.07 | X | 09.02.24 | MSN, PMH | Y |
| **Gunaga 2020^47^** | Survival to discharge | ICU admission | Response time | Total prehospital time | 01.01.17-31.03.17 | X | 09.02.24 | MSN, PMH | Y |
| **Han 2022^48^** | Survival to discharge | Neurological outcome* | Response time | Total transport time | 01.01.17-31.12.18 | X | 09.02.24 | MSN, PMH | Y |
| **Hayes 2010^49^** | Survival to discharge |  | Response time | Arrest to BLS time | 01.01.07-31.12.07 | X | 09.02.24 | MSN, PMH | Y |
| **Henry 2013^50^** | Survival to discharge | ROSC | Response time |  | 01.09.07-31.08.08 | X | 09.02.24 | MSN, PMH | Y |
| **Herlitz 2006^51^** | 30-day survival | ROSC | Call-to-arrival time* |  | 01.01.90-31.12.05 | X | 09.02.24 | MSN, PMH | Y |
| **Herlitz 2008^52^** | 30-day survival | ROSC | Response time | Call-to arrival time* | 01.01.90-31.12.05 | X | 09.0.24 | MSN, PMH | y |
| **Hillis 1993^53^** | Survival to discharge | Sickness Impact Profile | Response time |  | 01.01.87-31.10.89 | X | 09.02.24 | MSN, PMH | Y |
| **Holmen 2020^54^** | 30-day survival |  | Response time |  | 01.01.08-31.12.17 | X | 09.02.24 | MSN, PMH | Y |
| **Huabbangyang 2022^55^** | ROSC |  | Response time |  | 01.05.19-30.04.20 | X | 09.02.24 | MSN, PMH | Y |
| **Hubert 2016^56^** | Survival to discharge | CPC | Response time |  | 01.07.11-30.11.14 | X | 12.02.24 | MSN, PMH | Y |
| **Jennings 2006^57^** | Survival to discharge |  | Response time |  | 01.01.22-31.12.23 | X | 12.02.24 | MSN, PMH | Y |
| **Jeong 2017^58^** | Survival to discharge |  | Response time |  | 01.01.12-31.12.16 | X | 12.02.24 | MSN, PMH | Y |
| **Kennedy 2023^59^** | Survival to discharge |  | Response time |  | 01.03.20-31.12.21 | X | 12.02.24 | MSN, PMH | Y |
| **Kentsch 2000^60^** | Survival to discharge | Neurological outcome* | Response time |  | 01.01.84-31.12.88 and 01.01.91-31.12.97 | X | 12.02.24 | MSN, PMH | Y |
| **Kitano 2022^61^** | 30-day survival | ROSC | Response time | On-scene time | 01.01.14-31.12.19 | X | 12.02.24 | MSN, PMH | Y |
| **Klosiewicz 2017^62^** | ROSC |  | Response time | Call-to arrival time* | 01.01.15-31.12.15 | X | 12.02.24 | MSN, PMH | Y |
| **Lee 2019^63^** | Survival to discharge | Favorable neurological outcome | Response time |  | 01.10.15-31.12.16 | X | 12.02.24 | MSN, PMH | Y |
| **Lee 2013^64^** | Survival to discharge | CPC | Response time | Total transport time | 01.01.08-31.05.11 | X | 12.02.24 | MSN, PMH | Y |
| **Leung 2001^65^** | Survival to discharge |  | Response time |  | 15.03.99-15.10.99 | X | 12.02.24 | MSN, PMH | Y |
| **Lim 2020^66^** | Survival to discharge | ROSC | Response time | On-scene time | 01.01.11-31.12.16 | X | 12.02.24 | MSN, PMH | Y |
| **Lin 2014^67^** | Survival to discharge |  | Response time |  | 01.05.13-31.05.13 | X | 12.02.24 | MSN, PMH | Y |
| **Little 2020^68^** | Survival to discharge | ICU admission | Response time | Call-to-door time | 01.03.19-30.04.19 and 01.03.20-30.04.20 | X | 12.02.24 | MSN, PMH | Y |
| **Liu 2023^69^** | Survival to discharge | ROSC | Response time | Total prehospital time | 01.01.18-31.12.21 | X | 12.02.24 | MSN, PMH | Y |
| **Lyon 2013^70^** | Survival to discharge |  | Response time | Call-to-arrival time* | 01.09.10-01.09.11 | X | 12.02.24 | MSN, PMH | Y |
| **Margey 2011^71^** | Survival to discharge |  | Response time | On-scene time | 01.01.03-31.12.08 | X | 12.02.24 | MSN, PMH | Y |
| **Mathiesen 2018^72^** | Survival to discharge |  | Response time | Physician presence | 01.01.06-31.12.15 | X | 12.02.24 | MSN, PMH | Y |
| **Mayer 1979^73^** | Survival to discharge |  | Response time |  | 01.01.77-31.08.78 | X | 15.02.24 | MSN, PMH | Y |
| **Meyer 2001^74^**  **Mikiewicz 2023^75^** | Survival to discharge  Survival to discharge |  | Response time  Call-to-arrival time |  | 01.01.97-31.12.97  01.01.01-  31.12.21 | X | 15.02.24  30.10.24 | MSN, PMH  MSN, PMH | Y |
| **Mikkelsen 2017^76^** | 30-day survival | 90-day survival | Response time |  | 01.05.06-31.12.14 | X | 15.02.24 | MSN, PMH | Y |
| **Mills 2023^77^** | 30-day survival |  | Response time |  | 01.01.14-31.12.18 | X | 15.02.24 | MSN, PMH | y |
| **Mills 2019^78^** | 30-day survival | 24H-survival | Response time | Total prehospital time | 04.04.06-01.12.12 | X | 15.02.24 | MSN, PMH | Y |
| **Mogensen 2015^79^** | Survival to discharge |  | Response time |  | 01.01.04-31.12.07 | X | 15.02.24 | MSN, PMH | Y |
| **Morrisey 1996^80^** | 30-day survival |  | Response time | On-scene time | 01.01.91-31.12.91 | X | 15.02.24 | MSN, PMH | Y |
| **Nadolny 2021^81^** | Survival to discharge |  | Response time |  | 01.01.18-31.12.18 | X | 15,02,24 | MSN, PMH | Y |
| **Naroo 2012^82^** | 30-day survival |  | Response time |  | 01.04.11-30.07.11 | X | 15.02.24 | MSN, PMH | Y |
| **Navab 2019^83^** | Survival to discharge |  | Response time |  | 01.04.16-28.02.17 | X | 15.02.24 | MSN, PMH | Y |
| **Nichol 2016^84^** | Survival to discharge | Favorable neurological outcome | Response time | Call-to-arrival time* | 01.01.99-31.12.12 | X | 15.02.24 | MSN, PMH | Y |
| **Nielsen 2022^85^** | 30-day survival | 24H-survival | Response time |  | 01.10.14-30.09.18 | X | 15.02.24 | MSN, PMH | Y |
| **O'Keefe 2011^86^** | Survival to discharge | Favorable neurological outcome | Response time | Presenting rhythm | 01.01.96-31.12.00 | X | 16.02.24 | MSN, PMH | Y |
| **Park 2017^87^** | Survival to discharge | CPC | Response time |  | 01.01.12-31.12.14 | X | 16.02.24 | MSN, PMH | Y |
| **Pell 2001^88^** | Survival to discharge |  | Response time |  | 01.05.91-01.03.98 | X | 16.02.24 | MSN, PMH | Y |
| **Pons 2005^89^** | Survival to discharge |  | Response time | Total prehospital time | 01.01.98-31.12.98 | X | 16.02.24 | MSN, PMH | Y |
| **Pons 2002^90^** | Survival to discharge |  | Response time | Call-to arrival time* | 01.01.94-31.12.98 | X | 16.02.24 | MSN, PMH | Y |
| **Puolakka 2023^91^** | Survival to discharge | CPC | Response time |  | 01.01.16-31.12.21 | X | 16.02.24 | MSN, PMH | Y |
| **Do 2010^92^** | ROSC |  | Response time |  | 01.01.02-31.12.08 | X | 16.02.24 | MSN, PMH | Y |
| **Rajan 2016^93^** | 30-day survival |  | Response time |  | 01.01.05-31.12.11 | X | 16.02.24 | MSN, PMH | Y |
| **Renkiewicz 2014^94^** | ROSC |  | Response time | Presenting rhythm | 01.01.12-30.06.12 | X | 16.02.24 | MSN, PMH | Y |
| **Schinnerl 1990^95^** | Survival to discharge | Neurological outcome* | Response time |  | 18.11.85-17.11.88 | X | 16.02.24 | MSN, PMH | Y |
| **Semensato 2011^96^** | 30-day survival | CPC | Response time | Arrest to BLS time | 26.01.08-21.10.08 | X | 16,02,24 | MSN, PMH | Y |
| **Shah 2010^97^** | Survival to discharge |  | Response time |  | 01.04.03-31.03.07 | X | 16.02.24 | MSN, PMH | Y |
| **Shepherd 2008^98^** | Death* |  | Response time | On-scene time | 01.01.04-30.11.06 | X | 16.02.24 | MSN, PMH | Y |
| **Sigursson 2000^99^** | Survival to discharge |  | Response time |  | 01.01.91-31.12.96 | X | 16.02.24 | MSN, PMH | Y |
| **Sipria 2016^100^** | Survival to discharge | CPC | Response time | Resuscitation time | 01.01.99-31.12.13 | X | 16.02.24 | MSN, PMH | Y |
| **Spaite 2008^101^** | Survival to discharge |  | Response time | Total prehospital time | 01.10.04-31.12.06 | X | 16.02.24 | MSN, PMH | Y |
| **Stoeckl 2010^102^** | Survival to discharge | Neurological outcome* | Response time | Dispatch interval | 01.02.09-31.01.10 | X | 16.02.24 | MSN, PMH | Y |
| **Stromsoe 2011^103^** | 30-day survival |  | Population density |  | 01.01.08-31.12.09 | X | 16.02.24 | MSN, PMH | Y |
| **Stromsoe 2015^104^** | 30-day survival | CPC | Response time | Collapse-to-call time | 01.01.92-31.12.11 | X | 16.02.24 | MSN, PMH | y |
| **Sugiyama 2023^105^** | 30-day survival | Favorable neurological outcome | Response time | On-scene time | 01.03.20-31.09.22 | X | 16.02.24 | MSN, PMH | Y |
| **Syvaoja 2018^106^** | Survival to discharge | ROSC | Response time |  | 01.01.97-31.12.13 | X | 17.02.24 | MSN, PMH | Y |
| **TerAvest 2019^107^** | Survival to discharge | ROSC | Call-to-arrival time* |  | 01.01.13-01.01.18 | X | 17.02.24 | MSN, PMH | Y |
| **Thompson 2017^108^** | Survival to discharge | GCS | Transport interval | Call-to-arrival time* | 01.04.12-30.09.12 | X | 17.02.24 | MSN, PMH | Y |
| **Trenkler 2012^109^** | 30-day survival | ROSC | Response time |  | 01.01.11-31.12.11 | X | 17.02.24 | MSN, PMH | Y |
| **Tsai 2017^110^** | Survival to discharge |  | Response time | On-scene time | 01.01.12-31.12.15 | X | 17.02.24 | MSN, PMH | Y |
| **Wai 2005^111^** | Survival to discharge |  | Collapse-to-hospital time | Collapse-to-defibrillation time | 01.07.02-31.12.02 | X | 17.02.24 | MSN, PMH | Y |
| **Weinlich 2019^112^** | Survival to discharge | GOS | Response time | On-scene time | 01.01.09-31.12.13 | X | 17.02.24 | MSN, PMH | Y |
| **Wik 2003^113^** | Survival to discharge | CPC | Response time |  | 01.06.98-31.05.01 | X | 17.02.24 | MSN, PMH | Y |
| **Wissa 2021^114^**  **Zheng 2023^115^** | Survival to discharge  Survival to discharge |  | Response time  Response time |  | 01.01.15-31.12.19  01.08.19-  31.12.20 | X  x | 17.02.24  30.10.24 | MSN, PMH  MSN, PMH | Y  y |

**Legend:** BLS: Basic life support; GCS: Glasgow coma scale; CPC: Cerebral performance category; CPR: Cardiopulmonary resuscitation; Defib.: defibrillation; EMS: Emergency medical services; GOS: Glasgow outcome scale; GOSE: Extended Glasgow outcome scale; ICU: Intensive care unit; msn; Martine Siw Nielsen; LOS: Length of stay; NISS: New Injury Severity Scale; N/R: Not registered; PMH: Peter Martin Hansen; ROSC: Return of spontaneous circulation; X: Data extracted to data extraction and quality appraisal instruments; Y: Yes for eligible; 7D: 7-days; 24H: 24-hours
