# Supplementary material for: Association of ambulance and helicopter response times with patient survival: A systematic literature review and meta-analysis
Source: PLoS One. 2025 Nov 17;20(11):e0335665. doi: 10.1371/journal.pone.0335665 (PMC12622838; doi:10.1371/journal.pone.0335665)
Supplement: S5 Table — (DOCX) [file pone.0335665.s006.docx]

**REPOSITORY TABLE 5. Data or supporting information obtained from another source**

| **Reference** | **Author contacted** | **Contact date** | **Response date** |
| --- | --- | --- | --- |
| Björkman J, Setälä P, Pulkkinen I, Raatiniemi L, Nurmi J. Effect of time intervals in critical care provided by helicopter emergency medical services on 30-day survival after trauma. Injury. 2022 May;53(5):1596-1602. doi: 10.1016/j.injury.2022.01.025. Epub 2022 Jan 15. PMID: 35078619. | Johannes Bjorkmann (FIN) | 23 January 2024 | 24 January 2024 |
| Gregers MCT, Møller SG, Kjoelbye JS, Jakobsen LK, Grabmayr AJ, Kragh AR, et al. Association of Degree of Urbanization and Survival in Out-of-Hospital Cardiac Arrest. J Am Heart Assoc. 2023 May 16;12(10):e8322. doi: 10.1161/JAHA.122.028449. Epub 2023 May 9. PMID: 37158087; PMCID: PMC10227318. | Mads Chr. Tofte Gregers (DEN) | 29 January 2024 | 29 January 2024 |
| Kłosiewicz T, Skitek-Adamczak I, Zieliński M. Emergency medical system response time does not affect incidence of return of spontaneous circulation after prehospital resuscitation in one million central European agglomeration residents. Kardiol Pol. 2017;75(3):240-246. doi: 10.5603/KP.a2016.0181. Epub 2016 Dec 20. PMID: 27995600. | Tomasz Klosiewicz (POL) | 29 September 2023 | 8 February 2024 |
